# The DNA damage response to radiological imaging: from ROS and γH2AX foci induction to gene expression responses in vivo

**DOI:** 10.1007/s00411-023-01033-4

**Published:** 2023-06-19

**Authors:** Milagrosa López-Riego, Magdalena Płódowska, Milena Lis-Zajęcka, Kamila Jeziorska, Sylwia Tetela, Aneta Węgierek-Ciuk, Daniel Sobota, Janusz Braziewicz, Lovisa Lundholm, Halina Lisowska, Andrzej Wojcik

**Affiliations:** 1 Centre for Radiation Protection Research, Department of Molecular Biosciences, The Wenner-Gren Institute, Stockholm University, Stockholm, Sweden; 2 Department of Medical Biology, Institute of Biology, Jan Kochanowski University, Kielce, Poland; 3 Department of Medical Physics, Institute of Biology, Jan Kochanowski University, Kielce, Poland; 4 Department of Nuclear Medicine With Positron Emission Tomography (PET) Unit, Holy Cross Cancer Centre, Kielce, Poland

**Keywords:** Gene expression, γH2AX foci, ROS, Lymphocytes, Blood, Diagnostic imaging patients

## Abstract

Candidate ionising radiation exposure biomarkers must be validated in humans exposed in vivo. Blood from patients undergoing positron emission tomography–computed tomography scan (PET-CT) and skeletal scintigraphy (scintigraphy) was drawn before (0 h) and after (2 h) the procedure for correlation analyses of the response of selected biomarkers with radiation dose and other available patient information. *FDXR*, *CDKN1A*, *BBC3*, *GADD45A*, *XPC,* and *MDM2* expression was determined by qRT-PCR, DNA damage (γH2AX) by flow cytometry, and reactive oxygen species (ROS) levels by flow cytometry using the 2′, 7′—dichlorofluorescein diacetate test in peripheral blood mononuclear cells (PBMC). For ROS experiments, 0- and 2-h samples were additionally exposed to UVA to determine whether diagnostic irradiation conditioned the response to further oxidative insult. With some exceptions, radiological imaging induced weak γH2AX foci, ROS and gene expression fold changes, the latter with good coherence across genes within a patient. Diagnostic imaging did not influence oxidative stress in PBMC successively exposed to UVA. Correlation analyses with patient characteristics led to low correlation coefficient values. γH2AX fold change, which correlated positively with gene expression, presented a weak positive correlation with injected activity, indicating a radiation-induced subtle increase in DNA damage and subsequent activation of the DNA damage response pathway. The exposure discrimination potential of these biomarkers in the absence of control samples as frequently demanded in radiological emergencies, was assessed using raw data. These results suggest that the variability of the response in heterogeneous populations might complicate identifying individuals exposed to low radiation doses.

**Supplementary Information:**

The online version contains supplementary material available at 10.1007/s00411-023-01033-4.

## Introduction

Following genotoxic stress induced by the direct action of ionising radiation (IR) and, indirectly, by reactive oxygen species (ROS), the DNA damage response (DDR) is activated to preserve the integrity of the genome. Oxidative stress from water radiolysis is further amplified by ROS-producing cellular systems such as mitochondria (Reisz et al. 2014; Szumiel 2015). Early on in the DDR cascade, serine 139 of histone H2AX (γH2AX) is phosphorylated, which signals the presence of DNA double-strand breaks (DSBs) (Rogakou et al. 1998), one of the most deleterious DNA lesions (Schipler and Iliakis 2013). Downstream, a complex network of pro-survival or pro-death genes, usually p53-controlled (Hu et al. 2022), interact to determine the cellular fate (Christmann and Kaina 2013; Roos et al. 2016) both after environmentally-relevant (Amundson et al. 2000; Sokolov and Neumann 2015) and high doses (Beer et al. 2014; El-Saghire et al. 2013). Exploiting these molecular changes and the cytogenetic end-products that may follow IR exposure, a range of IR biomarkers have been proposed for use in biodosimetry (Swartz et al. 2010) or epidemiological studies (Hall et al. 2017; Pernot et al. 2012). The validation of these IR biomarkers requires appropriate human models.

Numerous validation efforts have been published for the γH2AX assay (Ainsbury et al. 2014; Barnard et al. 2015; Rothkamm et al. 2013) and gene expression (Abend et al. 2021, 2023, 2016; Badie et al. 2013; Biolatti et al. 2021; Manning et al. 2017) as IR biomarkers sensitive to doses in the mGy range (Schule et al. 2023). γH2AX was tested as a biomarker of DNA damage and repair in human population studies related to diagnostic procedures (Brand et al. 2012; Halm et al. 2014; Kuefner et al. 2009; Lobrich et al. 2005; Pathe et al. 2011; Rothkamm et al. 2007; Vandevoorde et al. 2015a), chemotherapy (Halicka et al. 2009; Karp et al. 2007; Sak et al. 2009) and/or radiotherapy (Sak et al. 2007; Zahnreich et al. 2015; Zwicker et al. 2011), as previously reviewed (Valdiglesias et al. 2013). The application of gene expression profiles in epidemiological studies has so far been limited by their transient nature (Hall et al. 2017). Nevertheless, transcription studies provide a valuable source of information for our understanding of cellular response to low doses (Sokolov and Neumann 2015), a dose range where stochastic effects are poorly defined due to larger uncertainties of epidemiological data (Kreuzer et al. 2018). Being able to reflect radiation exposure over a wide range of doses (Amundson et al. 2001; Amundson and Fornace 2001, 2003; Manning et al. 2013), transcriptomic biomarkers can also support dose reconstruction (Ghandhi et al. 2019a), triage (Port et al. 2019) and clinical outcome prediction (Port et al. 2016) in the event of radiological emergency. Fast readout, within hours to days, the possibility of high-throughput analysis (Ostheim et al. 2022), ease of sampling and high sensitivity early after exposure (Paul et al. 2011) add substantial value to their use in biodosimetry. In addition, the development of a transcriptomic dosimeter could help in estimating internal doses in radionuclide therapy and internal contamination, which currently relies on whole-body counting, biokinetic models as well as bioassays on urine or faecal samples (Edmondson et al. 2016).

Due to ethical considerations and given the restricted availability of suitable human samples, transcriptomic IR biomarker characterization usually entails mice (Ghandhi et al. 2019b), non-human primate models (Park et al. 2017; Port et al. 2018), and, to a large degree, human ex vivo exposed samples (Abend et al. 2021, 2016; Badie et al. 2013; Cruz-Garcia et al. 2018; Kaatsch et al. 2021, 2020; Kabacik et al. 2011; Knops et al. 2012; Manning et al. 2017; Nosel et al. 2013; Paul and Amundson 2011; Schule et al. 2023). Although these are relevant models to the human in vivo response (Lucas et al. 2014; O'Brien et al. 2018; Paul et al. 2011) and help to understand the potential impact of cofounding factors, e.g. inflammation (Mukherjee et al. 2019) or DNA repair capacity (Rudqvist et al. 2018), the problems of interspecies differences (Lucas et al. 2014; Satyamitra et al. 2022), blood cell deterioration, the absence of tissue signalling (Ghandhi et al. 2019a) and cellular microenvironment (Filiano et al. 2011) in culture are acknowledged. Consequently, candidate IR transcriptomic biomarkers must be validated in humans exposed in vivo (Paul et al. 2011).

The number of gene expression studies using in vivo IR-exposed human blood samples from either occupational (Fachin et al. 2009; Morandi et al. 2009; Sakamoto-Hojo et al. 2003), environmental (of natural or accidental origin) (Albanese et al. 2007; Jain and Das 2017), diagnostic or therapeutic exposures (Abend et al. 2016; Amundson et al. 2004; Campbell et al. 2017; Cruz-Garcia et al. 2022, 2018, 2020; Dressman et al. 2007; Edmondson et al. 2016; Evans et al. 2022; Filiano et al. 2011; Lucas et al. 2014; Meadows et al. 2008; O'Brien et al. 2018; Paul et al. 2011; Port et al. 2018; Riecke et al. 2012) is limited, but the available results clearly demonstrate the usefulness of transcript signatures as biomarkers of radiation exposure. Differential gene expression profiles are detected in human peripheral blood mononuclear cells (PBMCs) isolated from X- and γ-radiation-exposed health care workers exposed to doses < 25 mSv (Morandi et al. 2009). Also, an overrepresentation of DDR and p53-related genes is found among differentially expressed genes (DEGs) in PBMC of individuals living in high natural background radiation areas (Jain and Das 2017). For instance, *CDKN1A* is 1.5-fold upregulated in individuals exposed to > 15 mGy/year and *MDM2* is 1.5-fold upregulated in those exposed to 5–15 mGy/year (Jain and Das 2017). Differential expression of p53 target genes is also observed in samples from patients exposed, primarily, to external radiation (Abend et al. 2016; Amundson et al. 2004; Cruz-Garcia et al. 2022, 2018, 2020; Dressman et al. 2007; Filiano et al. 2011; Lucas et al. 2014; Meadows et al. 2008; O'Brien et al. 2018; Paul et al. 2011; Port et al. 2018; Riecke et al. 2012), with some exceptions (Campbell et al. 2017; Edmondson et al. 2016; Evans et al. 2022; Lee et al. 2015). Fold changes of ca 16, 7, and 3 are observed on average for *FDXR, CDKN1A*, and *BBC3*, respectively, in patients exposed to 1.25 Gy total body irradiation (TBI) (Paul et al. 2011). *FDXR* upregulation was shown to increase with the dose from diagnostic CT scans to TBI and radiotherapy (O'Brien et al. 2018).

In this study, we sought to correlate the radiation dose with the expression of *FDXR*, *CDKN1A*, *BBC3*, *GADD45A*, *XPC* and *MDM2* along with levels of γH2AX and ROS in PBMC of patients undergoing positron emission tomography–computed tomography scan (PET-CT) and skeletal scintigraphy (scintigraphy). Blood was collected before and 2 h after the diagnostic intervention so that the individual background and radiation-induced signal levels could be compared. Additionally, correlation analyses with available individual information such as age, sex, or records of previous radiotherapy or chemotherapy treatment were performed to assess the impact of these factors on the studied biomarkers of exposure.

## Materials and methods

### Donor information and blood sample collection

Blood samples were obtained at the Department of Nuclear Medicine with the Positron Emission Tomography Unit of the Holy Cross Cancer Centre in Kielce (Poland) from patients undergoing diagnostic PET-CT (*n* = 17) and scintigraphy (*n* = 17). Sampling was carried out randomly during the period of March-June 2022 mostly on Monday and Tuesday, in the morning hours. On one day, between two and three patients were sampled that underwent the diagnostic procedure consecutively. Blood was collected by venipuncture in EDTA tubes (BD Vacutainer) and into PAXgene® Blood RNA tubes (BD Biosciences) before (0 h) and after (2 h) the diagnostic procedure. Each blood sample was coded with a letter corresponding to the procedure (P for PET-CT and S for skeleton scintigraphy), a number ranging from 1 to 17 and the blood collection time point (0 and 2). For PET-CT patients, the effective dose from the injected activity is shown in Table 1 along with the total effective dose, in brackets, which includes the effective dose from CT scanning. The effective dose from CT was assumed to correspond to 6.8 mSv for males and 7.9 mSv for females, based on the literature (Kaushik et al. 2015). Scintigraphy patients did not undergo CT examination during the procedure. Blood was collected separately for gene expression analysis and the other endpoints. For gene analysis and activity measurements 2.5 ml blood was directly collected into PAXgene® Blood RNA tubes (see below for details), for the other endpoints, into EDTA tubes. Samples were stored at room temperature and were transported to the Jan Kochanowski University (Kielce) between 30 and 60 min after the 2 h blood sampling. Transport to the university took 15–20 min. After arrival, radioactivity was measured in the PAXgene® tubes at room temperature. The tubes were then gradually frozen according to the guidelines of the manufacturer (− 20 °C followed by − 80 °C), shipped in two batches on dry ice to Stockholm University (September 2022 and December 2022) and stored there at − 80 °C until further processing. EDTA samples were processed for the endpoints described below within 60 min of their arrival.

**Table 1:** Cohort information

Treatment		Patient code	Sex (M/F)	Age (years)	Body mass (Kg)	Diagnosis	RT record (year)	CHT record (year)	Injected activity (MBq)	E (ET) dose (mSv)	Blood analysis activity (mBq)	Percent left (%)
Scintigraphy	^99m^Tc-MDP	S-01	F	51	84	Scintigraphy	2021	2021	714	4.71	12.60	1.76
		S-02	M	67	98	Scintigraphy			745	4.92	10.50	1.41
		S-03	F	71	62	Scintigraphy			718	4.74	NA	
		S-04	M	67	89	Scintigraphy			740	4.88	14.00	1.89
		S-05	M	69	83	Scintigraphy			771	5.09	4.70	0.61
		S-06	M	80	65	Scintigraphy			720	4.75	14.90	2.07
		S-07	M	75	120	Scintigraphy		2021	746	4.92	12.00	1.61
		S-08	M	62	83	Scintigraphy			729	4.81	9.44	1.29
		S-09	F	43	81	Scintigraphy	2020	2021	726	4.79	16.20	2.23
		S-10	M	69	85	Scintigraphy			706	4.66	12.00	1.70
		S-11	M	73	110	Scintigraphy			721	4.76	12.60	1.75
		S-12	M	53	100	Scintigraphy	2022	2022	738	4.87	5.79	0.78
		S-13	M	75	81	Scintigraphy			755	4.98	11.70	1.55
		S-14	M	67	90	Scintigraphy			718	4.74	13.30	1.85
		S-15	M	68	107	Scintigraphy	2009		731	4.82	12.50	1.71
		S-16	M	73	86	Scintigraphy			NA	NA	NA	
		S-17	M	78	NA	Scintigraphy			712	4.70	10.30	1.45
PET	^18^F-FDG	P-01	M	72	75	Lung C, SCC	2014		252	6.80 (13.6)	5.60	2.22
		P-02	F	76	74	Melanoma			217	5.86 (13.76)	6.66	3.07
		P-03	F	47	57	Sarcoma	2019	2019	202	5.45 (13.35)	4.39	2.17
		P-05	M	53	88	Thymus C	2021	2021	287	7.75 (14.55)	6.90	2.40
		P-09	M	49	83	H&N C	2020, 2021		290	7.83 (14.63)	5.52	1.90
		P-10	M	75	85	Lung C, SCC			284	7.67 (14.47)	6.93	2.44
		P-11	M	74	94	Bronchus C			307	8.29 (15.09)	4.26	1.39
		P-12	M	69	97	D38.1			313	8.45 (15.25)	1.06	0.34
		P-13	M	41	90	Hodgkin L		2021	287	7.75 (14.55)	4.97	1.73
		P-14	M	70	57	D38.1			193	5.21 (12.01)	5.64	2.92
		P-15	M	48	101	Pancreas C			339	9.15 (15.95)	4.17	1.23
	^18^F-PSMA	P-06	M	70	107	Prostate C			362	7.96 (14.76)	6.38	1.76
		P-07	M	71	86	Prostate C			359	7.90 (14.7)	4.93	1.37
		P-08	M	72	87	Prostate C			362	7.96 (14.76)	8.67	2.40
		P-16	M	79	69	Prostate C			243	5.35 (12.15)	15.10	6.21
		P-17	M	65	84	Prostate C			258	5.68 (12.48)	2.86	1.11
	^68^Ga-DOTATATE	P-04	M	71	83	C75.9			155	3.98 (10.78)	0.26	0.17

17 PET-CT (P) patients treated with ^18^F-FDG, ^18^F-PSMA and ^68^ Ga-DOTATATE and 17 skeletal scintigraphy (S) patients treated with ^99m^Tc-MDP were included in this study. Each individual was coded with a letter attending to the corresponding diagnostic procedure (P/S) and a number 1–17. Information regarding: sex, age, body mass, diagnosis, year of previous radiotherapy (RT) or chemotherapy (CHT) treatment, if applicable, and injected activity was recorded. SCC: squamous cell carcinoma. H&N: head and neck. D38.1: Neoplasm of uncertain behaviour of trachea, bronchus and lung. C75.9: Malignant neoplasm of endocrine gland, unspecified. Effective dose (E dose, mSv/mBq) from injected activity was calculated using the following conversion factors: 0.0066 (for ^99m^Tc-MDP), 0.027 (^18^F-FDG), 0.022 (^18^F-PSMA) and 0.0257 (^68^ Ga-DOTATATE). The total effective dose (ET) for PET-CT patients, shown in brackets, corresponds to E from the injected activity plus the effective dose from CT scanning: 6.8 mSv for males and 7.9 mSv for females, based on Kaushik et al. (2015). The total effective dose for scintigraphy patients was that from the injected activity because they did not undergo CT scanning. The activity left in blood at 2-h post-treatment was measured with an HPGe detector in Bq and converted to mBq, corresponding to numerical values shown in the blood analysis activity column. The percent of injected activity left was calculated based on the injected activity and the activity left in blood at 2 h

For analysis of γH2AX foci and ROS, peripheral blood mononuclear cells (PBMC) from ca 5 ml of each blood sample were isolated by gradient centrifugation. To this end, blood was diluted 1:1 in phosphate-buffered saline (PBS) and overlaid on LymphoprepTM (Serumwerk Bernburg AG for Alere Technologies AS, Oslo, Norway) and centrifuged at 400×*g* for 30 min. The layer containing lymphocytes was removed and washed three times with phosphate-buffered saline (PBS). Cells were counted and 1/3 was used for analysis of γH2AX and 2/3 for analysis of ROS as described in detail below.

**Fig. 1:**
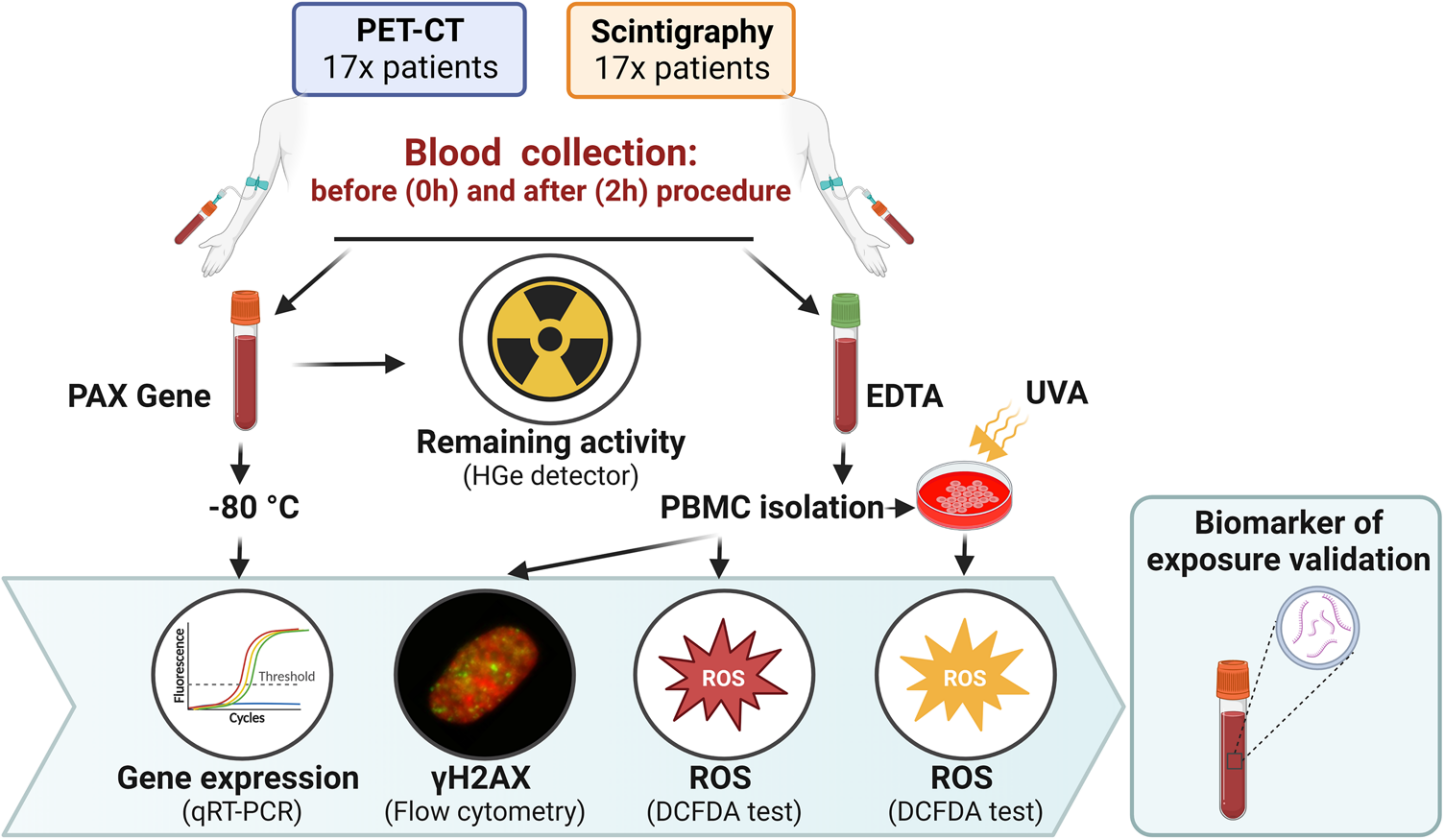
Experimental setup. Blood was drawn from 17 PET-CT patients and 17 scintigraphy patients before (0 h) and after (2 h) the corresponding diagnostic procedure with the aim of validating biomarkers of ionising radiation exposure. Gene expression analyses were performed by qRT-PCR on stabilised RNA from whole blood to determine the level of expression of six radiation-responsive genes: *FDXR*, *CDKN1A*, *MDM2*, *GADD45A*, *BBC3* and *XPC*. The level of γH2AX and ROS were assessed by flow cytometry, using the 2′, 7′—dichlorofluorescein diacetate (DCFDA) test for the latter. For ROS, blood samples were additionally tested after exposure to UVA at the two time points. The activity of isotopes in 2 h blood samples was measured by a germanium detector. Created with BioRender.com

The general experimental setup is graphically shown in Fig. 1. Available information regarding the individuals included in this study such as sex, age, body mass or records of previous radiotherapy (RT) or chemotherapy (CHT) is provided in Table 1. The cohort included 29 males and 5 females, with ages ranging from 41 to 80, with an average age of 66. For scintigraphy imaging, the ^99m^Tc- methylene diphosphonate (^99m^Tc-MDP) was used. PET patients were diagnosed with squamous cell carcinoma (SCC), melanoma, sarcoma, thymus cancer, head and neck cancer (HNC), lung cancer, bronchus cancer, neoplasm of uncertain behaviour of trachea, bronchus and lung (D38.1), Hodgkin lymphoma, pancreas cancer, prostate cancer or malignant neoplasm of the endocrine gland (C75.9). They received either ^18^F-Fluorodeoxyglucose (^18^F-FDG), [^18^F]-labelled prostate-specific membrane antigen (^18^F-PSMA) or ^68^Ga-DOTA-Phe1 Tyr3-octreotate (^68^Ga-DOTATATE). The study was approved by the ethical committee of the Regional Medical Chamber in Kielce.

### Gene expression analysis

RNA extraction was performed using the PAXgene Blood RNA Kit (PreAnalytiX), following the manufacturer´s instructions. cDNA was synthesised using the High-Capacity cDNA Reverse Transcription Kit (Thermo Fisher Scientific) from 95 ng RNA, to maximise the RNA input in the reaction based on the lowest RNA concentration sample. For real-time PCR, duplicate reactions of primers, cDNA and 5 × HOT FIREPol® EvaGreen® qPCR Supermix (Solis BioDyne) were setup and run on a LightCycler® 480. The cycling conditions were: 95 °C (15 min), 40 cycles of 95 °C (15 s), 60 °C (20 s) and 72 °C (20 s). The 2^−ΔΔCt^ method was used for the calculation of relative expression, using the housekeeping *18S* gene for normalisation. Primer specificity was confirmed using melting curve analysis. Forward (for) and reverse (rev) primers (5′–3′) used were described earlier for genes of interest (Cheng et al. 2018) and housekeeping 18S (Lundholm et al. 2014). These were: *GADD45a_*for (actgcgtgctggtgacgaat), *GADD45a_*rev (gttgacttaaggcaggatccttcca), *BBC3*_for (tacgagcggcggagacaaga), *BBC3*_rev (gcaggagtcccatgatgagattgtac), *MDM2*_for (tatcaggcaggggagagtgataca), *MDM2*_rev (ccaacatctgttgcaatgtgatggaa), *XPC*_for (gcttggagaagtaccctacaagatggt), *XPC*_rev (ggctttccgagcacggttaga), *FDXR*_for (tggatgtgccaggcctctac), *FDXR*_rev (tgaggaagctgtcagtcatggtt), *CDKN1A*_for (cctggagactctcagggtcgaaa), *CDKN1A*_rev (gcgtttggagtggtagaaatctgtca), *18S*_for (gcttaatttgactcaacacggga), *18S*_rev (agctatcaatctgtcaatcctgtcc). Gene expression results here represent an average of the response of all leukocytes, including peripheral blood lymphocytes and granulocytes, lysed in the PAXGene system.

### γH2AX analysis

Isolated PBMC were washed once in PBS and fixed for 10 min in Cytofix Fixation Buffer (Becton Dickinson, Cat. No. 554655). Cells were washed again in PBS, 90% methanol (Chempur, Poland, chilled at − 20 °C) which was added drop by drop and left for permeabilization for 5 min. Cells were washed in Perm/Wash Buffer (Becton Dickinson, Cat. No. 554723), incubated with Alexa Fluor 647 Mouse anti-H2AX pS139 (Becton Dickinson, Cat. No. 560447) for 60 min and washed with Perm/Wash. Cells were resuspended in 300 µl Stain Buffer (FBS, Becton Dickinson, cat. no. 554656) and the level of γ-H2AX fluorescence was measured with an LSR II flow cytometer (Becton Dickinson USA). Alexa Fluor 647 was excited by the red laser (627–640 nm) and detected using an optical filter centred near 520 nm (e.g. a 660/20 nm bandpass filter). The BD FACS DiVa (version 6.0, Becton Dickinson) was used for data acquisition and analysis. 20,000 events were stored. Per sampling time and patient, the median focus intensity was calculated and used for analyses.

### ROS analysis

Oxidative stress induced by UVA was quantified with the help of the 2′,7′—dichlorofluorescein diacetate (DCFDA) test (Sigma Aldrich, D6883). PBMC incubated for 15 min in the stain buffer at 37 °C), then DCF was added for 30 min. Cells were split into two Petri dishes. One was irradiated with UVA on ice (see below) and the other was sham-exposed. Next, the cells were transferred to cytometer tubes and the level of fluorescence was measured with an LSR II flow cytometer (Becton Dickinson, USA). A computer system BD FACS DiVa (version 6.0, Becton Dickinson) was used for data acquisition and analysis. Data for 20,000 events were stored. Per sampling time and patient, the median signal intensity was calculated and used for analyses.

UV irradiation was carried out with a 2G11 55 W DULUX L BL lamp, OSRAM, Germany, operating in the UVA range (315–400 nm). The irradiation time was 20 min and the UV dose was 0.3 kJ/cm^2^. Dosimetry was carried out with a CHY 732 320–400 nm UVA metre, CHY FIREMATE Co., LTD, UK. The dose was selected based on unpublished results from student projects where it was found to induce a strong signal.

### Activity measurements and effective dose calculations

The activity of blood in each PAXgene® tube was measured using a nitrogen cooled high purity germanium (HPGe) detector (model GX3020-b12075) placed in a shielded container and connected to a Genie™ 2000 Spectroscopy Software, Canberra Industries, Inc, USA. Prior to measuring the activity of blood samples, the spectrometer was pre-calibrated with calibration sources containing ^99m^Tc and ^18^F isotopes of known activity. The activity of the blood samples was measured approximately 2–4 h after sampling 2 h samples and converted to the sampling time based on the half-life of the specific radioisotope.

The injected radionuclide activities were documented for each patient and converted to effective doses using the isotope-dependent conversion factors (mSv/mBq): 0.0066 for ^99m^Tc-MDP (presented by Batista da Silva et al. in Congresso Brasiliano de Metrologia das Radiacoes Ionizantes, Rio de Janeiro, 28.11.2018) 0.027 for ^18^F-FDG (ICRP 1988), 0.022 for ^18^F-PSMA (Giesel et al. 2017) and 0.0257 for ^68^Ga-DOTATATE (Walker et al. 2013).

### Statistical analyses

Results from 0 h collection times were compared to 2 h using paired *t* tests or one-way ANOVA with multiple comparison corrections. Results from scintigraphy and PET patients were compared using unpaired *t* tests. *p* values are provided together with Cohen’s effect size *d* values, in accordance with the claim that scientific conclusions should not be solely based on significance tests (Amrhein et al. 2019). The following criteria were applied for effect sizes: *d* < 0.5: small effect; *d* = 0.5–0.8: medium effect; *d* = 0.8–1.3: large effect; *d* > 1.3: very large effect (Cohen 1988). *t* tests (paired and unpaired), one-way ANOVA, linear regression analyses (*Y* = slope*X + *Y*-intercept), and correlation analyses to obtain Pearson r- values were performed using GraphPad Prism 9.4.1. Detailed results of the analyses are provided in supplemental tables as specified in the text and figure legends.

## Results

### PET-CT and scintigraphy induce weak fold changes in gene expression, γH2AX foci and ROS relative to unexposed samples

Gene expression of a panel of six radiation-responsive genes was analysed in PBMC by qPCR before (0 h) and 2 h after PET-CT and scintigraphy. The fold change of each gene at 2 h relative to the control (0 h) was calculated for each of the 17 patients per group. PBMC were also analysed for DNA damage by the γH2AX focus test by flow cytometry and for ROS levels by the DCFDA test using flow cytometry. For ROS analysis, aliquots of PBMC collected at 0 h and 2 h were exposed to UVA to determine the possible impact of PET-CT and scintigraphy on the response of cells to oxidative stress induced by a strong oxidative insult. The activity of radionuclides in the blood collected at 2 h was measured with a germanium detector (Fig. 1).

**Fig. 2:**
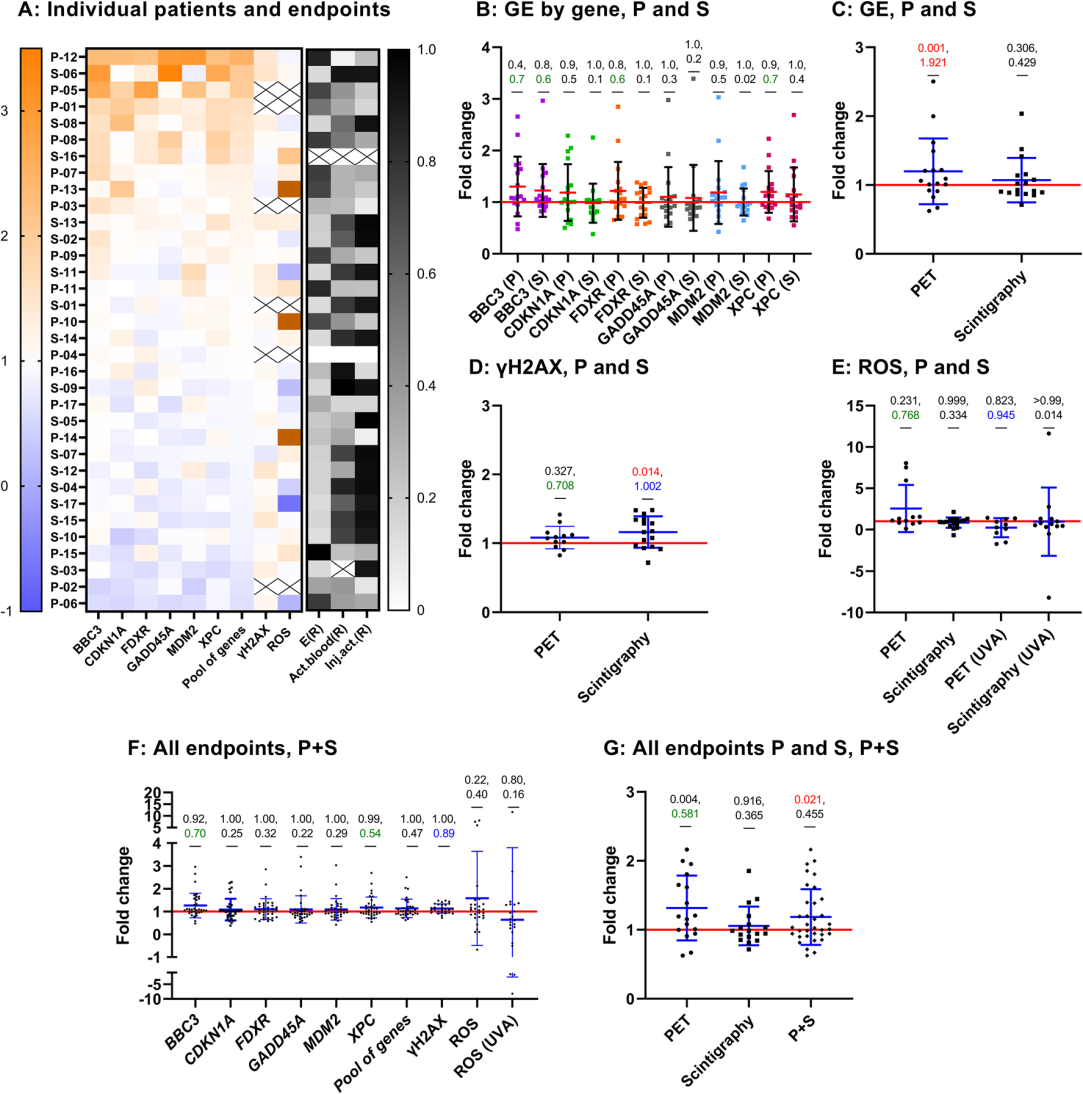
Fold changes for PET-CT (PET, P) and scintigraphy (S) patients. **A** heatmap of fold changes for all endpoints in each patient, with individuals ranked by highest to lowest overall responsiveness in gene expression. Donor-matched effective dose (E, mSv), activity (act.) left in blood at 2 h (mBq), and injected activity (inj. act., MBq) are shown in the right heatmap panels relative (R) to a scale 0–1, whereby 1 corresponds to the maximum and 0 to the minimum value observed for each of these three parameters. Black crosses indicate unavailable data. ROS fold changes > 3.5 are shown in brown. B-G represent scatter dot plots for gene expression (GE) fold changes of each gene (**B**) or the average of the pool of genes (**C**); γH2AX fold change (**D**); ROS fold change after the diagnostic procedure only, or after additional UVA exposure (**E**); fold change of each endpoint for the pool of all patients (**F**); average fold change of all endpoints pooled per donor (excluding UVA results) (**G**). ROS (UVA): 2 h samples exposed to UVA relative to 0 h samples exposed to UVA. Each symbol represents one patient. A red horizontal line represents a fold change equivalent to control values (*Y* = 1). *p* values (top) shown in red if *p* < 0.05 and effect size *d* values (bottom) shown in green (medium, > 0.5–0.8), blue (large, > 0.8–1.3) or red (very large, ≥ 1.3), Supplemental Table 1. Mean and standard deviation are shown by red and black bars, respectively (**B**), or by blue bars (**C**–**G**)

To graphically visualise summarised results of all assays, a heat map was constructed where the results of all assays are presented as fold changes of data from 2 h over 0 h. Patients were ranked from highest to lowest with respect to fold gene expression (Fig. 2A). Per patient, the six analysed genes responded fairly similarly as can be judged by the relatively uniform horizontal colour patterns: orange at the top rows and blue at bottom rows. No obvious relationship can be seen at this projection between gene expression, γH2AX, ROS and activity measurements. High effective dose values (black and dark grey boxes) clustered in the top 17 rows, suggesting a positive relationship with gene expression. More results presented as fold changes are shown in Fig. 2 and described in greater detail below.

Figure 2B shows the individual and mean results per gene and patient group. None of the genes was significantly upregulated at 2 h, but some showed a medium effect size such as *BBC3* in PET-CT patients (*p* = 0.41, *d* = 0.74) and scintigraphy patients (*p* = 0.75, *d* = 0.63), *FDXR* (*p* = 0.78, *d* = 0.55) and *XPC* (*p* = 0.87, *d* = 0.69) in PET-CT patients only. Unpaired t test was used to test whether the PET-CT and scintigraphy patients differed in the gene expression response. For none of the genes there was a statistically significant difference, Supplemental Table 1. Effect sizes were small, with the exception of *FDXR*_,_ for which a medium effect size (*d* = 0.52) was detected. In an attempt to test if the diagnostic radiation exposure had an impact on overall gene expression per patient, the average fold changes of all 6 genes were calculated. The results are shown in Fig. 2C. The mean fold change of all genes per patient was somewhat higher and more spread out in the group of PET-CT (1.20 ± 0.15) as compared to scintigraphy (1.07 ± 0.24) patients. The increase in fold change was significant (*p* = 0.001) and of large size (*d* = 1.92) in PET-CT patients but not significant (*p* = 0.31) and small (*d* = 0.43) for scintigraphy patients. The difference in gene fold change between both patient groups was not significant (*p* = 0.37) but medium (*d* = 0.65). Individual gene expression results are shown in Supplemental Fig. 1 for PET-CT and in Supplemental Fig. 2 for scintigraphy patients.

Figure 2D shows the results of the γH2AX test. In contrast to gene expression, a higher level of response was detected in scintigraphy then in PET-CT patients: the fold change in the former patient group was significant (*p* = 0.014) and of large size (*d* = 1.00), while in the latter group, it was not significant (*p* = 0.33) and of medium size (*d* = 0.708). Individual γH2AX test results for both patient groups are shown in Supplemental Fig. 3. The results of ROS analyses are presented in Fig. 2E. Except for 3 PET-CT patients, ROS fold changes in samples not additionally exposed to UVA were not distinguishable from 1 meaning that the diagnostic radiation exposure did not induce detectable oxidative stress. Exposure of 2 h samples to UVA radiation did not induce significant ROS level changes relative to 0 h samples exposed to UVA in any of the groups. However, a large effect, with a downregulation pattern, was seen in PET-CT patients (*p* = 0.82, *d* = 0.95), but not in scintigraphy patients (*p* > 0.99, *d* = 0.01). This indicated that the diagnostic exposure of patients did not change the response of PBMC to additional oxidative stress induced by UVA irradiation. Complete numerical values of statistical tests are given in Supplemental Table 1.

To increase the statistical power of the analysis, fold changes from the two patient groups were pooled (Fig. 2F). The result of gene expression and γH2AX was non-significant and of small size (except *BBC3* and *XPC*, for which the effect sizes were medium, and γH2AX, for which the effect size was large, see Supplemental Table 1 for *p* and *d* values). Highly scattered results were obtained for ROS without UVA with 2 donors showing fold changes of around 8. As expected, based on the results shown in Fig. 2E, additional UVA exposure did not lead to significant effects (*p* = 0.8, *d* = 0.16). Moreover, with the aim of seeing whether the combination of fold changes from all endpoints improved the power to detect radiation exposure, fold changes were pooled separately for PET-CT and scintigraphy patients and for both groups of patients together. The results are shown in Fig. 2G. Interestingly, the pooling of fold changes per patient group resulted in a somewhat higher and more spread-out values in the PET-CT as compared to the scintigraphy cohort. Finally, the pooling of all patients resulted in a significant but small mean fold change, with 1 out of 34 patients showing a combined fold change above 2, and 9—above 1.3. A twofold change relative to control is considered a conservative threshold which controls for false positive results (Riecke et al. 2012), but a fold change threshold of 1.3 is also considered biologically relevant (Jain and Das 2017).

### Correlation of gene expression, γH2AX foci and ROS changes with patient characteristics

**Fig. 3:**
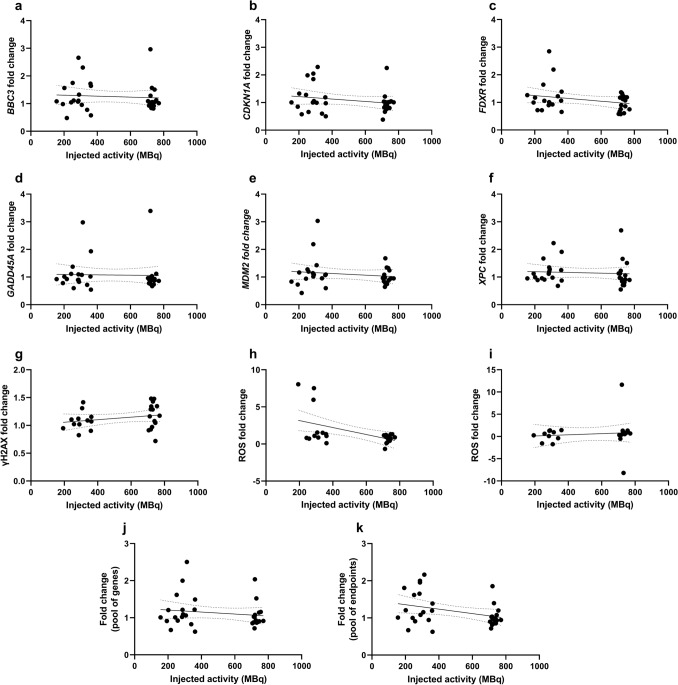
Correlation of injected activity (MBq) with fold change results from each endpoint considering the pool of all patients. Gene expression fold changes for *BBC3* (**A**), *CDKN1A* (**B**), *FDXR* (**C**), *GADD45A* (**D**), *MDM2* (**E**), and *XPC* (**F**). **G** γH2AX fold change. **H**–**I** ROS fold changes in blood samples 2 h after PET (P) or scintigraphy (S) procedure as compared to control samples at 0 h (H) or ROS fold changes in blood samples 2 h after PET (P) or scintigraphy (S) procedure and additional UVA exposure as compared to control samples at 0 h exposed to UVA (I). **J** Average fold change per patient for the pool of genes. **K** Average fold change per patient for the pool of endpoints (excluding ROS UVA). Each symbol represents one individual. Linear regressions (Supplemental Table 2) are represented with a black solid bar and 95% confidence intervals are represented with dotted black bands

**Fig. 4:**
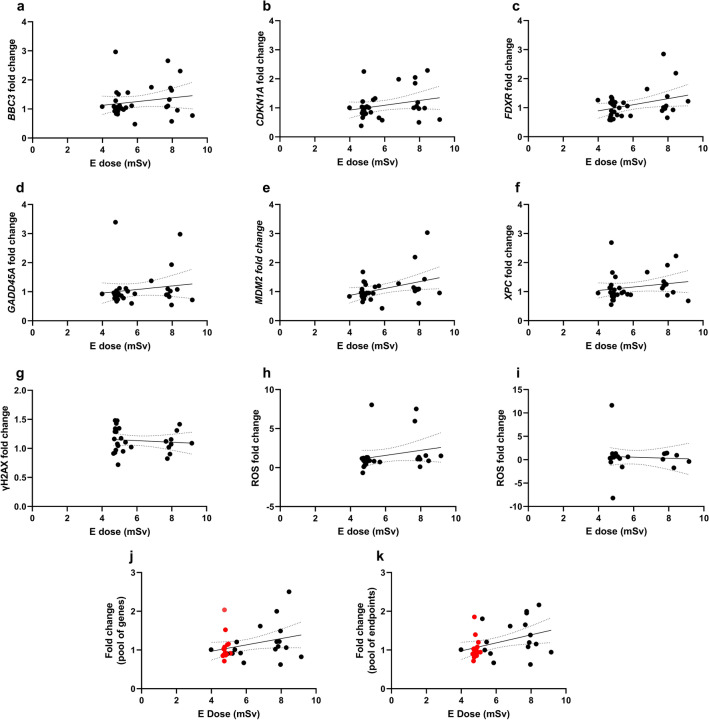
Correlation of effective (E) dose (mSv) from injected activities with fold change results from each endpoint considering the pool of all patients. Gene expression fold changes for *BBC3* (**A**), *CDKN1A* (**B**), *FDXR* (**C**), *GADD45A* (**D**), *MDM2* (**E**), and *XPC* (**F**). **G** γH2AX fold change. **H**–**I** ROS fold changes in blood samples 2 h after PET (P) or scintigraphy (S) procedure as compared to control samples at 0 h (H) or ROS fold changes in blood samples 2 h after PET (P) or scintigraphy (S) procedure and additional UVA exposure as compared to control samples at 0 h exposed to UVA (I). **J** Average fold change per patient for the pool of genes. **K** Average fold change per patient for the pool of endpoints (excluding ROS UVA). Each symbol represents one individual. Scintigraphy patients are shown in red in panels **J** and **K**. Linear regressions (Supplemental Table 3) are represented with a black solid bar and 95% confidence intervals are represented with dotted black bands

**Fig. 5:**
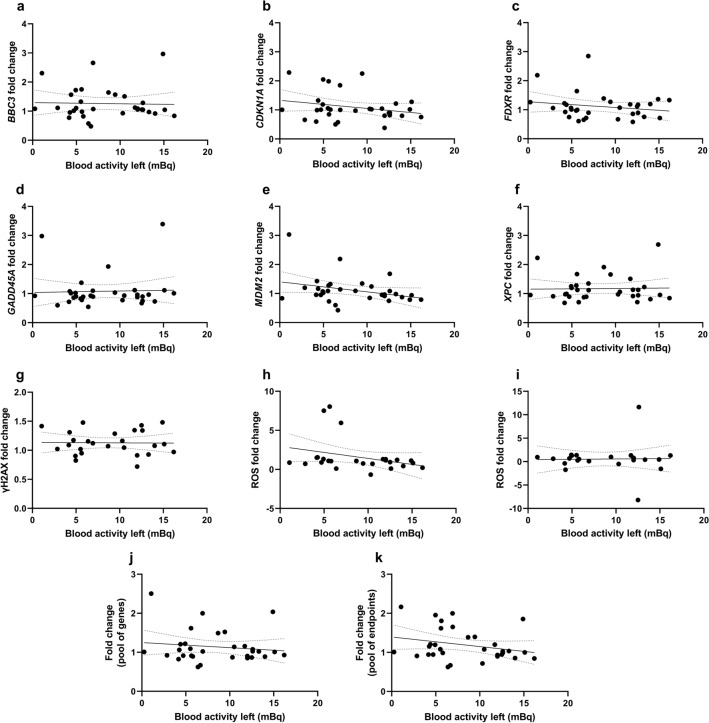
Correlation of blood activity left (mBq) with fold change results from each endpoint considering the pool of all patients. Gene expression fold changes for *BBC3* (**A**), *CDKN1A* (**B**), *FDXR* (**C**), *GADD45A* (**D**), *MDM2* (**E**), and *XPC* (**F**). **G** γH2AX fold change. **H**–**I** ROS fold changes in blood samples 2 h after PET (P) or scintigraphy (S) procedure as compared to control samples at 0 h (H) or ROS fold changes in blood samples 2 h after PET (P) or scintigraphy (S) procedure and additional UVA exposure as compared to control samples at 0 h exposed to UVA (I). **J** Average fold change per patient for the pool of genes. **K** Average fold change per patient for the pool of endpoints (excluding ROS UVA). Each symbol represents one individual. Linear regressions (Supplemental Table 4) are represented with a black solid bar and 95% confidence intervals are represented with dotted black bands

Fold changes observed for the pool of patients for each of the endpoints were correlated to injected activity (Fig. 3), calculated effective dose (Fig. 4), blood activity left in blood at 2 h post-procedure (Fig. 5) and percent of injected activity left at 2 h (Supplemental Fig. 5). In these figures, panels A-K show the corresponding correlations for *BBC3*, *CDKN1A*, *FDXR*, *GADD45A*, *MDM2*, *XPC*, γH2AX, ROS (after PET or scintigraphy only or after additional UVA exposure), pool of genes and pool of endpoints, respectively. Additional correlation analyses were performed to determine how the different fold changes correlated between endpoints (Supplemental Fig. 6). Numerical values regarding linear regressions of these correlations, including equations, 95% confidence intervals, *R*^2^ and Pearson *r* values are provided in Supplemental Tables 2, 3, 4, 5, 6. Overall, there was a lack of steep slopes with low *R*^2^ and *r* values.γH2AX fold change presented a weak positive correlation with injected activity (Fig. 3G and Supplemental Table 2). Conversely, ROS fold change (Fig. 3H) as well as the fold change for pool of all endpoints (Fig. 3K) seemed to correlate inversely with injected activity, yet interpretations should be cautious given the scatter of data. There was a weak trend of positive correlation of effective dose with gene expression, ROS, and the pool of endpoints fold changes (Fig. 4, Supplemental Table 3). Correlation patterns with activity left in blood at 2 h post-procedure (Fig. 5) or percent of injected activity left at 2 h (Supplemental Fig. 5) were weak, and in some cases, driven by few individuals such as the apparent negative correlation observed for ROS with activity left in blood at 2 h (Fig. 5H, Supplemental Table 4).

Supplemental Fig. 6 shows correlation analyses of fold changes in γH2AX *vs.* either gene expression or ROS, as well as correlations of fold changes in ROS *vs.* either gene expression or ROS plus additional UVA exposure. Numerical values for these correlations are provided in Supplemental Table 6. Results indicated that γH2AX correlated positively with gene expression (Supplemental Fig. 6A–F) and with a weak pattern of negative correlation with ROS fold changes (Supplemental Fig. 6M). Furthermore, there were no clear correlations between ROS and gene expression fold changes, with, maybe, the exception of *CDKN1A* (Supplemental Fig. 6H), yet with a weak positive correlation. Also, there was no clear correlation between the ROS levels after the diagnostic procedure and additional UVA exposure-induced ROS in 2 h samples (Supplemental Fig. 6N).

Further analyses were performed to assess the correlation of the observed fold changes with age (Supplemental Fig. 7), body mass (Supplemental Fig. 8), sex (Supplemental Fig. 9), and previous radiotherapy (Supplemental Fig. 10) or chemotherapy treatment (Supplemental Fig. 11). The numerical results are provided in Supplemental Tables 7, 8, 9, 10, 11 and demonstrate weak correlations with the observed fold changes. However, the advanced age of a large proportion of patients, the limited number of females included in the study, and few patients with records of previous radiotherapy or chemotherapy regimen make the interpretation of these results difficult.

### The potential of gene expression, γH2AX foci and ROS as biomarkers of low-dose exposure in the absence of a control

**Fig. 6:**
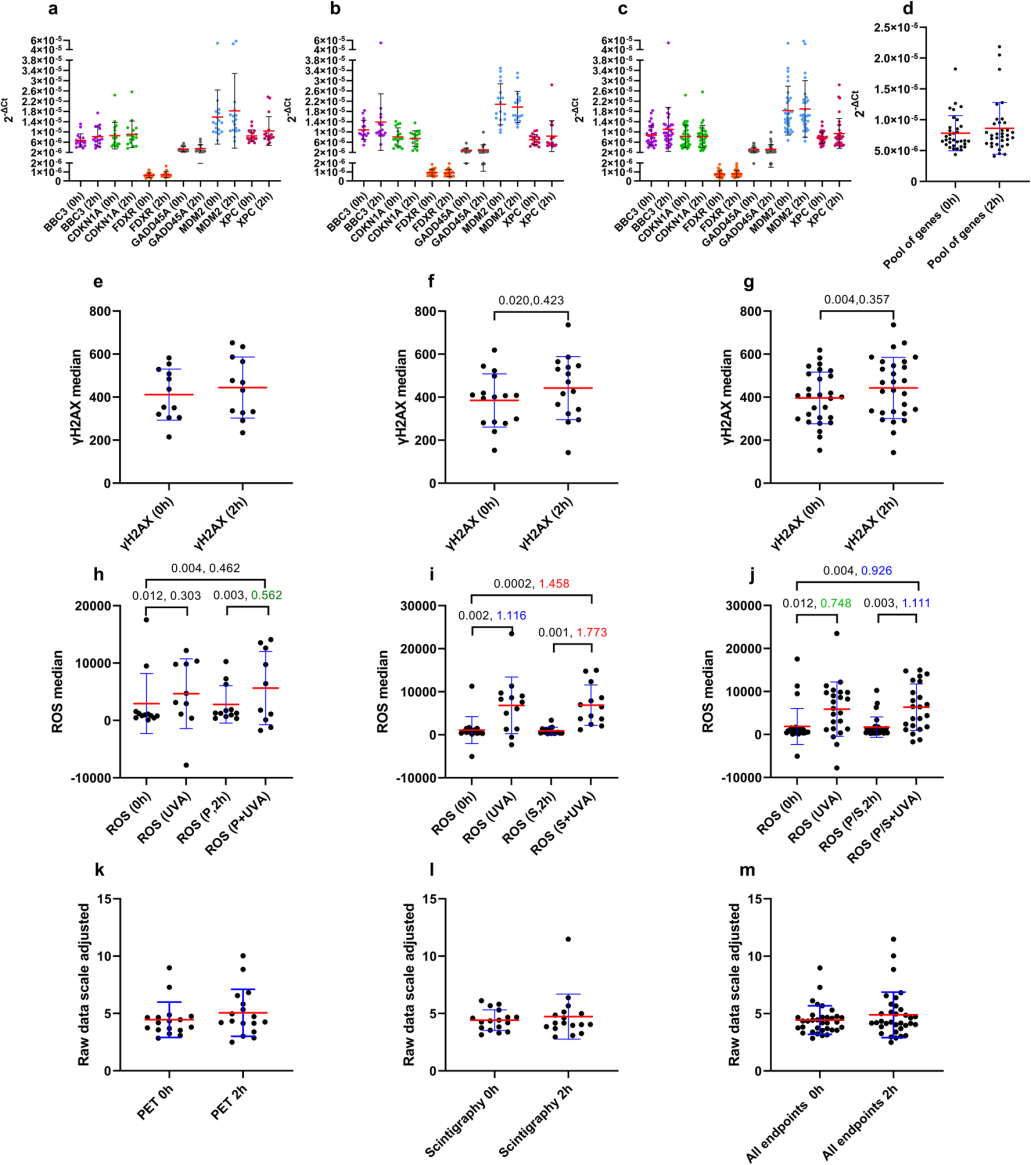
Raw data (2^−ΔCt^) at 0 and 2 h for PET (P) and scintigraphy (S) patients. Scatter dot plots representing 2^−ΔCt^ gene expression for individual genes in PET patients (**A**), scintigraphy patients (**B**), pool of patients (**C**) and the pool of genes for all patients (**D**). Mean and standard deviations are represented by red and black bars, respectively (**A**–**C**), or by red and blue bars, respectively (**D**–**I**). Median γH2AX fluorescence intensity for PET patients (**E**), scintigraphy (**F**), or the pool of patients (**G**). Median ROS levels for PET patients (**H**), scintigraphy (**I**), or the pool of patients (**J**). ROS UVA: ROS after UVA exposure in 0 h samples. ROS P/S + UVA: ROS after UVA exposure in 2 h samples. Scatter dot plot for the average raw results of all endpoints pooled (excluding UVA results) at 0 and 2 h for PET patients (**K**), scintigraphy patients (**L**) or the pool of patients (**M**), whereby the following factors were applied to raw data to get them to the same range (adjusted scale): *BBC3* (× 500,000); *CDKN1A* (× 1,000,000); *FDXR* (× 1,000,000); *GADD45A* (× 1,000,000); *MDM2*: (× 300,000); *XPC* (× 1,000,000); γH2AX (÷ 100); ROS (÷ 1500). p values (left) are shown if p < 0.05 and effect size d values (right) are shown in green (medium, > 0.5–0.8), blue (large, > 0.8–1.3) or red (very large, ≥ 1.3), Supplemental Table 12. Each symbol represents one patient

Because in the event of a radiological emergency control samples are rarely available, and consequently, it is not possible to normalise data to an unexposed biological material, the response of each endpoint was further assessed based on raw data without normalisation to individual samples before the diagnostic procedure (Fig. 6). These were: 2^−ΔCt^ values for gene expression, median γH2AX and median ROS values. To compare results at 2 h vs. 0 h, one-way ANOVA with Šidák correction for multiple comparisons was used for gene expression and ROS since, for these endpoints, more than two factors were analysed (Supplemental Table 12). For γH2AX, paired t-test was used. The 2^−ΔCt^ values of blood samples at 2 h did not differ statistically from those at 0 h for either PET or scintigraphy patients, Fig. 6A and B, respectively, and Supplemental Table 12. To increase the statistical power by considering all patients, which could be used to identify cohorts of people exposed to low-dose radiation in a radiological emergency, PET-CT and scintigraphy patients were pooled. Gene expression at 2 h samples was compared to that at 0 h based on raw 2^−ΔCt^ values for all patients pooled (Fig. 6C). ANOVA results, provided in Supplemental Table 12, indicated that the expression of none of the genes was significantly different from control samples at 2 h. Moreover, 2^−ΔCt^ values of the pool of genes were not statistically different between these two timepoints based on paired t-test (Fig. 6D). The median γH2AX intensity differed significantly at 0- and 2 h in scintigraphy patients alone (*p* = 0.02, 0.42), Fig. 6F, as well as the pool of all scintigraphy and PET patients (*p* = 0.004, 0.36), Fig. 6G, but not in PET patients alone (Fig. 6E). ROS levels were only significantly different from control values in samples exposed to UVA (Fig. 6H and I). Both groups of patients, separately or pooled (Fig. 6J), showed different ROS levels in UVA-exposed samples at 0 h as compared to 0 h controls, 2 h samples as compared to 0 h controls, and 2 h samples as compared to 2 h controls, Supplemental Table 12. Importantly, when endpoints were pooled either for PET patients only (Fig. 6K), scintigraphy patients only (Fig. 6L) or the pool of all patients (Fig. 6M), exposed samples could not be discriminated from unexposed samples, Supplemental Table 12.

Correlation analyses were then performed with raw data at 0- or 2 h as justified below. Raw data at 0 h was correlated with patients’ age (Supplemental Fig. 12), body mass (Supplemental Fig. 13) and sex (Supplemental Fig. 14), as these should, ideally, not determine the response of the analysed endpoints. Consequently, the absence of correlation would be desirable as it would indicate a high inclusiveness value of the corresponding biomarker. Also, raw data at 0 h would most likely not correlate with previous records of RT (Supplemental Fig. 15) or CHT (Supplemental Fig. 16) provided that these did not occur shortly before. No clear correlations were observed (Supplemental Figs. 12, 13, 14, 15, 16) with the exception of a weak inverse correlation of ROS levels after UVA exposure with age (Supplemental Fig. 12I). We further tested how the different endpoints correlated based on raw data at 0 h as these could indicate the activity level of a potential background DNA damage response (Supplemental Fig. 17). With the exception of a weak positive correlation of *CDKN1A* with γH2AX (Supplemental Fig. 17B), no clear correlations were identified in these analyses.

A strong correlation of raw data at 2 h with injected activity (Supplemental Fig. 18), effective dose (Supplemental Fig. 19), blood activity remaining at 2 h (Supplemental Fig. 20) and the percent of injected activity remaining in blood at 2 h (Supplemental Fig. 21) would indicate the potential of these biomarkers to discriminate individuals exposed to low doses, even in the absence of an unexposed control. These results indicated, however, only a weak pattern of increased 2^−ΔCt^ values, i.e. higher expression, with higher effective doses for *CDKN1A* (Supplemental Fig. 19B) and *XPC* (Supplemental Fig. 19F), but with large data scatter. Driven by a few individuals, a weak positive correlation was found for ROS levels with effective dose (Supplemental Fig. 19H) and for *BBC3* expression with activity in blood at 2 h (Supplemental Fig. 20A). Finally, correlation analyses between endpoints for raw data at 2 h (Supplemental Fig. 22), which would ideally represent the coordinated induction of the DNA damage response at different levels after exposure to these low doses, resulted in generally flat slopes, and low R^2^ and r values, with the exception of a positive slope observed for *CDKN1A *vs. γH2AX (Supplemental Fig. 22B).

## Discussion

DNA damage, ROS levels and gene expression changes induced by PET-CT or scintigraphy exposure were determined using blood collected before and after the diagnostic procedures and correlated to patients’ data to further characterise these endpoints as biomarkers of IR exposure. Although correlation analyses revealed generally mild slopes and low r values, γH2AX fold change presented a weak positive correlation with injected activity, indicating that exposure from these diagnostic procedures induced a subtle increase in DNA damage (Fig. 3). To note, each individual was sampled before and after the procedure, acting as his/her own control, being a strength of this study. Consistent with the overall weak fold changes at the level of γH2AX, as well as ROS, the expression changes in the panel of radiation-responsive genes tested at 2 h post-procedure were also generally low relative to control samples (Fig. 2). Some patients did show consistent upregulation of several endpoints, e.g. patient P-12, S-06 and P-05, while others showed downregulation, e.g. patients P-06 and P-02.

The reason for the variability in the response between patients who received comparable injected activities or effective doses remains unclear. P-12 and S-06, who were among those with the highest γH2AX levels (DNA damage data were not available for P-05) showed, together with P-05, the highest gene expression upregulation. The effective doses received by P-12 and P-05 were in the high range. S-06, whose effective dose was lower than P-12 or P-05, had, nevertheless, received a high injected activity and showed high remaining activity at 2 h (Fig. 2A and Table 1). Conversely, P-06 and P-02 showed gene expression downregulation and reduced ROS levels (if available) relative to control, while their effective doses were also high, yet had intermediate injected activity in relative terms (Fig. 2A). For internal exposures, the biokinetics and radionuclide decay have a strong impact on gene expression in white blood cells, which indeed correlates best with the kinetics of dose decay (exponential decay of activity) rather than with total absorbed dose (Edmondson et al. 2016). This, however, did not seem to explain the observed differences between P-12, S-06 and P-05 with P-06 and P-02 patients considering the comparable amount of activity lost during the 2 h gap since administration.

It should be mentioned that the analysed endpoints were correlated with effective doses from injected radionuclide activities in both groups of patients. However, the E in PET-CT patients included the dose from CT scanning which, however, was constant for all patients with a small difference between males (6.8 mSv) and females (7.9), as assessed by Kauschik et al. (2015) for PET-CT investigations relevant to our study. Omitting E from CT was justified by the fact that we were primarily interested in detecting signal differences between individual patients and not patient groups. Moreover, an important analysis was the clearance of activity in the blood for which E from CT scanning is irrelevant. As it can be observed in Fig. 2 A, patients were not clustered according to the procedure indicating the major driver of signal variability was the dose from injected radionuclides. This conclusion is supported by results shown in Fig. 4J and K, where a similar range of signals is seen in S and P patients. Including E from CT scanning would shift the total dose of PET-CT patients to the right without impacting the conclusion.

The different responses observed in patient samples could also be related to diverse pathophysiological stages (Whitney et al. 2003), different individual radiosensitivity (Badie et al. 2008) and/or variable activation of the DDR following low doses (Lee et al. 2015). A panel of genes predictive for radiation toxicity has been previously described (Rieger et al. 2004), although it does not include the genes analysed here. P-05 had previously undergone radiotherapy, but information regarding tissue reaction was not available. Up- or downregulation patterns have been observed earlier for different individuals undergoing interventional imaging procedures for the *CDKN1A*, *FDXR*, *GADD45A* and *MDM2* genes (Visweswaran et al. 2019), and in patients undergoing SPECT myocardial perfusion imaging (MPI) for the *MDM2* and *BBC3* genes (Lee et al. 2015). Furthermore, the relative induction of *CDKN1A* and *GADD45A* ranged between ca two- and- sevenfold and between one- and sevenfold, respectively, in patients diagnosed with different malignancies 6 h after the first 1.5 Gy fraction of TBI (Amundson et al. 2004). Some degree of variation in *FDXR* expression was observed in healthy human donors even at 0 h (O'Brien et al. 2018), and in lymphoblastoid cells from different individuals 12 h post 10 Gy exposure, for *CDKN1* and *GADD45A* in addition to *FDXR* (Jen and Cheung 2003). It seems that there is a lower interindividual variation for *FDXR*, which is expressed at a low level endogenously (Manning et al. 2013), than for *CDKN1A* (Abend et al. 2016). However, large interindividual variability in some *FDXR* variants appears in response to TBI exposure, with CVs between 19.6 and 46 depending on the variant (Cruz-Garcia et al. 2020). Although not within the scope of this study, it would be interesting to monitor these patients for tissue response provided upcoming radiotherapy treatment. Another interesting follow-up would be the analysis of genetic polymorphisms, as variations in the *trans* regulators of radiation-induced expression genes seem major determinants of the phenotype (Smirnov et al. 2009).

To statistically compare the changes observed at 2 h to those at 0 h, PET and scintigraphy patients were pooled, first per group, and then altogether. This approach was justified not only from the perspective of gaining statistical power, but importantly, because radiological emergencies may involve a wide range of doses, radiation qualities, and individuals with unique profiles. IR exposure biomarkers should, ideally, discriminate exposed and unexposed individuals in a heterogenous population independently, or with a moderate- to- low impact, of disease or infection status (O'Brien et al. 2018; Paul et al. 2011), prior exposure to chemotherapy (Lucas et al. 2014), sex (Cruz-Garcia et al. 2018, 2020; Lucas et al. 2014; O'Brien et al. 2018) and anti-oxidant levels (O'Brien et al. 2018). Lifestyle factors such as alcohol use, impact both radiation-induced and basal γH2AX levels, which may also be confounded by age and/or ethnicity (Sharma et al. 2015). A non-significant trend of increased H2AX, p53 and ATM phosphorylation was observed in lymphocytes from 20 to 25-year-old individuals compared to a 40–55 age group following a 25 mGy dose (Lee et al. 2015). Age and sex contribute, however, less than 20–30% to the total inter-individual variance in gene expression, which is considered negligible, as their impact on fold changes still falls within the two-fold equivalent to control values (Agbenyegah et al. 2018). At high doses, provided the use of optimal gene expression biomarkers, nor smoking (Paul and Amundson 2011), inflammation status (Budworth et al. 2012; Mukherjee et al. 2019) or sex (Cruz-Garcia et al. 2018) compromise the use of transcription biomarkers for triage purposes. In our study, correlations of γH2AX, gene expression, and ROS fold changes with age (Supplemental Fig. 7) were unclear, partly due to the bias towards older individuals. Moreover, no clear correlations were observed between raw data at 0 h and body mass (Supplemental Fig. 13), sex (Supplemental Fig. 14), previous record of RT (Supplemental Fig. 15) or CHT (Supplemental Fig. 16) indicating a high inclusiveness value of the studied biomarkers, albeit with the limitation of small sample size for some variables. Besides, with the exception of a weak positive correlation of *CDKN1A* with γH2AX at 0 h (Supplemental Fig. 17B), no clear correlations between endpoints were identified at 0 h which would suggest an active DNA damage response in these patients before the diagnostic examinations (Supplemental Fig. 17). To note, these biomarkers may be induced by several other exogenous and/or endogenous stressors such as UV, hypoxia or cellular replication. Despite the potential influence of confounding factors in their response and their low specificity, γH2AX, redox levels and gene expression hold much promise as biomarkers of early response to ionising radiation (Hall et al. 2017).

Some words should be mentioned with respect to uncertainties associated with the results. The first uncertainty is related to sample fixation time after injection as well as to the stability of the studied markers. Small fluctuations in fixation time due to variations in transport and processing time, as well as waiting time-between consecutive patients, could contribute to the observed interindividual variability. The impact of time for both gene expression and oxidative stress, for which changes can be detected days after exposure, is likely less significant than for γH2AX, which appears to have much faster kinetics and less stability (Hall et al. 2017). One important limitation to the use of the γH2AX assay as a biomarker of radiation exposure is precisely the short stability of the signal, which usually persists only for some minutes- to- hours (Hall et al. 2017). γH2AX foci have been shown to peak at 15–30 min and to decrease to baseline levels by 120 min (Lee et al. 2015), although sample incubation at 4 °C after exposure help to reduce signal decay (Vandevoorde et al. 2015b). As described in the materials and methods section, the maximum total processing time was approximately 140 min. It is plausible, that the γH2AX mean fluorescence intensity would have been higher had onsite sample processing and earlier fixation been logistically feasible. Moreover, the well-known time-dependency of gene expression changes (Macaeva et al. 2019), demands optimal time point selection for the assessment of the response to IR at the transcription level. For example, the *FDXR* gene has been shown to peak at 8 h in radiotherapy patients (with a mean fold change of 2.7), and to become significantly upregulated relative to control at 24 h (mean fold change of 1.8) (Cruz-Garcia et al. 2022). Similarly, *CDKN1A* was shown to increase significantly at 24 h post first radiotherapy fraction, with a mean fold change of 1.6, while neither *BBC3*, *MDM2* or *GADD45A* were significantly upregulated over a 24 h time period post low doses to the blood (Cruz-Garcia et al. 2022). *CDKN1A*, *FDXR*, *GADD45A*, *XPC*, and *MDM2* were significantly upregulated (maximum fold change of ca 8.5 for *FDXR*) at 72 h post-^131^I- metaiodobenzylguanidine (^131^I- mIBG) exposure (at doses in the range 2–3 Gy and calculated doses to the blood of 0.45–1.97 Gy) in neuroblastoma paediatric patients as shown in two related studies (Edmondson et al. 2016; Evans et al. 2022). These genes were also upregulated (sixfold upregulation for *FDXR*) in ^131^I- mIBG-irradiated patients at 96 h post-exposure, although with a general pattern of downregulation as compared to the 72 h timepoint (Edmondson et al. 2016). At 15 days after exposure, the fold changes of *CDKN1A* and *GADD45A* were not different from control, while *MDM2* and *XPC* became downregulated (ca 0.7-fold), and *FDXR* remained upregulated (ca 1.5-fold), indicating that the DDR may be active long after exposure (Evans et al. 2022). Thus, it is also plausible that a different magnitude of the response could have been detected had other time points (and/or gene-sets) been chosen. ROS produced as a consequence of water radiolysis have a lifetime of nanoseconds to seconds (Dikalov and Harrison 2014), so radiation-induced ROS levels passed this time point, as detected hours post-exposure, is most likely driven by redox homeostasis alterations in the cell (Shimura et al. 2016), but further research is needed to elucidate the effects of mitochondrial ROS at low doses (Kawamura et al. 2018). Considering the dynamic nature of γH2AX foci (Lee et al. 2015) and of gene expression changes (Cruz-Garcia et al. 2022; Edmondson et al. 2016; Evans et al. 2022; Kabacik et al. 2015; Macaeva et al. 2019), it would have been interesting to study additional time points after exposure, which was, unfortunately, not possible due to sampling constraints for practical reasons in the clinic. Nevertheless, the available 2 h timepoint was justified for gene expression analyses based on previously reported in vivo data (Lee et al. 2015; O'Brien et al. 2018).

The second uncertainty is related to the calculated effective dose E. E is a concept used in radiological protection and it describes the tissue-weighted sum of equivalent doses in all specified tissues and organs of the body (Harrison et al. 2023; Martin 2011). The radiation weighting factors used for calculating equivalent doses are based on averaged relative biological effectiveness factors of a given radiation type. The tissue weighting factors are calculated based on results from epidemiological studies on radiation-induced cancer and represent average values from people of different ages and sex. Hence, E does not provide a measure that is specific to the characteristics of an exposed individual and there are large uncertainties in the accuracy for numerical estimations of the risk of cancer derived from E for a reference person, especially in nuclear medicine (Martin 2011). A more detailed discussion of these uncertainties is beyond the scope of the study, but they should not be overlooked. We calculated *E* values for the patients based on models developed for the used radionuclides because they take into account the biodistributions of the radionuclides. The aim was to see if the correlation with the measured levels of biomarkers was better than when based solely on the administered radiation activity. This was not the case which demonstrates the complexity associated with detecting low radiation doses by biological markers. Coherent with the higher activity administered to the scintigraphy patients (mean injected activity of 731 ± 17.5 MBq) than to the PET-CT patients (277 ± 61.7 MBq), and the twice as high mean activity left in blood at 2 h in the scintigraphy patients, scintigraphy induced a statistically significant upregulation of γH2AX levels as compared to control (Fig. 2D). This was not detected for PET-CT, despite a medium effect size. The statistical significance of γH2AX fold change at 2 h was lost when scintigraphy and PET groups were pooled (Fig. 2F). However, the induction of γH2AX still correlated positively with gene expression fold changes (Supplemental Fig. 6A–F). In line with this finding, up-regulation of γH2AX (Halm et al. 2014; Rothkamm et al. 2007; Vandevoorde et al. 2015a) and *FDXR* expression (O'Brien et al. 2018) were detected after low computed tomography doses. Also, those SPECT MPI patients with increased γH2AX after the procedure showed significant upregulation of DDR-related genes such as *Tp53* and *MDM2* (Lee et al. 2015). Using blood samples exposed ex vivo with a CT scanner, both radiation-induced foci (RIF) and *FDXR* expression increased linearly with dose at comparable unit rates, with a significant increment relative to control at 11.3 mGy up to 49.7 mGy for a threefold RIF, and at 22.6 mGy up to 49.7 mGy for a fourfold *FDXR* expression (Schule et al. 2023).

Consistent with a generally low level of induced DNA damage, only weak trends of gene expression upregulation relative to control were observed. These included *BBC3*, *XPC* and *GADD45A* genes in both groups and *CDKN1A*, *FDXR* and *MDM2* in PET-CT patients only (Fig. 2B). *BBC3* showed a medium effect size (with large p-value) in scintigraphy and PET patients, which also applied to *FDXR* and *XPC* in PET patients only. *FDXR* is one of the most IR-responsive genes in PBMC (Cheng et al. 2019; Manning et al. 2013) and among those with the best dose discrimination power (Lacombe et al. 2018). *BBC3, XPC* and *CDKN1A* are also identified as top predictor genes of radiation response in humans (Dressman et al. 2007). The upregulation of these genes could indicate an initial cell cycle arrest through *CDKN1A* (Brugarolas et al. 1995) and *GADD45A* (Wang et al. 1999) (albeit PBMC are not dividing); a pro-apoptotic response in heavily damaged cells, led by *BBC3* (Chipuk et al. 2005; Jeffers et al. 2003) and *FDXR* (Hwang et al. 2001; Liu and Chen 2002; Zhang et al. 2017) and the activation of DNA damage repair through *XPC* (Adimoolam and Ford 2002; Sugasawa et al. 1998). For both groups together, *BBC3* and *XPC* expression at 2 h remained with a medium effect size (Fig. 2F).

The weak gene expression changes observed on average here are in agreement with previously reported small changes in *FDXR* expression (1.3–1.7-fold change) in 6 out of 8 patients at 2 h after CT exposure with estimated doses to the blood of 3.9–20.9 mGy (O'Brien et al. 2018). *CDKN1A* showed a ca 27-fold increase while *GADD45A* remained close to control levels 6 h after 1.5 Gy delivered by TBI treatment in a non-Hodgkin’s lymphoma patient (Amundson et al. 2004). *CDKN1A* and *GADD45A* showed a ≤ twofold upregulation relative to control 2 h after CT scan with estimated doses of 10–43 mGy and after 6 mGy ^18^F-FDG injection followed by a 0.2 cGy (2 mGy) CT scan (Riecke et al. 2012). *FDXR* was, however, significantly upregulated 24 h after TBI, and continuously during fractionated treatment for several malignancies (O'Brien et al. 2018). Following the first fraction with a 0.038–0.169 Gy dose to the blood, the mean fold changes in six radiotherapy patients are 1.45, for *FDXR*; 1.67, for *CDKN1A*; 1.08, for *BBC3*; 1.09, for *MDM2*; and 1.02, for *GADD45* (Cruz-Garcia et al. 2022). When considering the overall gene expression response by the pool of genes, PET patients showed a statistically significant upregulation, but not scintigraphy patients, Fig. 2C. This observation was interesting in light of the already discussed higher activity injected in the scintigraphy group and might reflect dose-dependent kinetics of transcription.

In agreement with gene expression results, only the PET group showed a medium effect size for ROS upregulation, which was not statistically significant (Fig. 2E). Additional exposure of samples to UVA radiation did not induce a significant increase of ROS in scintigraphy patients (*p* > 0.99, *d* = 0.01) nor in PET-CT patients, who showed, nevertheless a large effect size with a downregulation pattern (*p* = 0.82, *d* = 0.95). This suggests that the diagnostic exposure of patients did not change the impact of UVA irradiation in PBMC. It should be noted, however, that low doses of radiation were reported to induce oxidative stress leading to oxidised nucleotides in the cellular cytoplasm (Haghdoost et al. 2006; Sangsuwan and Haghdoost 2008). A weak trend of positive correlation of ROS fold change with effective dose was found (Fig. 4H), but correlations of this endpoint with injected activity (Fig. 3H), activity remaining in blood at 2 h (Fig. 5H) or percent of injected activity remaining at 2 h (Supplemental Fig. 5H) were weak or unclear due to large data scatter. That ROS fold changes, gene expression and the pool of endpoints presented a weak but positive correlation with effective dose (Fig. 4), manifested the relevance of considering isotope-specific conversion factors to account for different biodistributions, despite the fact that beta-emitters deposit most of their emitted energy locally, i.e. within the blood, tumour or target organs (Edmondson et al. 2016).

A secondary, yet relevant aspect of our study was the assessment of gene expression, γH2AX foci and ROS as biomarkers of in vivo low-dose exposure even in the absence of a control sample, such as following a radiological emergency. In such situations, it has been suggested that cycle threshold (ΔCT) values may serve as exposure indicators provided that RNA amount and quality input are precisely controlled (Edmondson et al. 2016). This strategy has been successfully applied ex vivo (Brzoska and Kruszewski 2015; Paul and Amundson 2008) and in vivo (Abend et al. 2016). Non-irradiated and irradiated samples in the range of 1.25 Gy (one fraction) to 3.75 Gy (delivered in three fractions) were discriminated with high accuracy in patients receiving total body irradiation (Dressman et al. 2007; Filiano et al. 2011; Lucas et al. 2014; Meadows et al. 2008; Paul et al. 2011). Moreover, blood samples of prostate cancer patients receiving intensity-modulated radiation therapy (IMRT) were discriminated from preexposure control samples based on *FDXR* expression at 24 h after equivalent blood doses as low as 0.09–0.017 Gy (Abend et al. 2016). Exposed and unexposed samples were also discriminated after internal exposures using a panel of genes including *FDXR* and *CDKN1A* (Edmondson et al. 2016; Evans et al. 2022). The aim here was to test whether 0- and 2 h samples could be discriminated based on raw data (Fig. 6).

Exposed samples were not significantly different from unexposed samples when using the pool of all endpoints for PET (Fig. 6K) and scintigraphy patients alone (Fig. 6L) or all patients pooled (Fig. 6M). ROS levels only deviated from control values in UVA-exposed samples (Fig. 6H and I) and none of the individual genes (Fig. 6C), nor the pool of genes (Fig. 6D), had a significantly different expression from control samples at 2 h when considering all patients pooled. However, the median γH2AX intensity at 2 h differed significantly from that at 0 h in scintigraphy patients alone (Fig. 6F) and the pool of all patients (Fig. 6G). γH2AX foci analysed by immunofluorescence microscopy revealed a significantly enhanced frequency in PBMC after low doses of X-radiation delivered during neuro-interventional procedures (Visweswaran et al. 2020), not significant for the increase in γH2AX mean fluorescence intensity in post-diagnostic (observed in 64.5% of patients) and post-therapeutic (50% of patients) neuro-interventional procedures as compared to pre-exposure controls (Visweswaran et al. 2019). Moreover, the percentage of γH2AX positive cells at 30 min post-SPECT was not significant as compared to baseline levels in a different study (Lee et al. 2015). The γH2AX relative fluorescence intensity was found to correlate poorly with the entrance surface dose values, i.e. absorbed dose by the skin in a particular region or organ, measured with a thermoluminescence dosimeter in patients undergoing neuro-interventional diagnostic (*p* = 0.199, *R*^2^ = 0.0563) and therapeutic (*p* = 0.617, *R*^2^ = 0.015) procedures from 9- to 225 mGy (Visweswaran et al. 2019). Besides, the expression of *CDKN1A* (0.55-fold change), *MDM2* (0.57-fold) and *FDXR* (0.84-fold), and *GADD45A* (1.1-fold) did not differ statistically from control samples 24 h after low doses of neuro-interventional radiological procedures (Visweswaran et al. 2019). In line with this, correlation analyses between the tested endpoints for raw data at 2 h (Supplemental Fig. 22) did not reveal a clear induction of the DNA damage response after exposure to the tested low doses when considering raw data without normalisation.

The shape of the dose–response curve for cellular effects after low doses and low dose rates is largely uncertain. While the radiation protection system is quantitatively valuable, implicit assumptions in risk estimation associated with low doses and protracted IR exposures would benefit from stronger evidence through further experimental data (Kreuzer et al. 2018; Shore et al. 2017). Biomarkers of IR exposure help to understand the molecular and cytogenetic effects of low doses, to be considered in epidemiological studies (Hall et al. 2017; Pernot et al. 2012) or biodosimetry applications (Swartz et al. 2010). This demands, however, appropriate validation of biomarkers by using biological samples exposed in vivo, whose availability is usually limited for obvious reasons. We further characterised γH2AX, ROS levels and transcriptomic changes as biomarkers of IR exposure in vivo using blood from patients undergoing PET-CT and skeletal scintigraphy. γH2AX fold changes correlated weakly, but positively, with injected activity, indicating a radiation-induced increase in DNA damage with dose. γH2AX upregulation also correlated positively with mild changes in the transcription of known radiation-responsive genes, suggesting a coherent activation of the DDR pathway following diagnostic imaging-induced genotoxic stress. For reasons to be determined, some patients showed consistently stronger up- or down-regulation of several endpoints after comparable injected activities or effective doses, which could relate to differences in radiosensitivity and/or DDR activation after low doses (Lee et al. 2015). This cohort included 29 males and 5 females, with ages ranging from 41 to 80 and an average age of 66. Given the relatively small population, with a bias towards older and male individuals, it would be highly desirable to expand this study by increasing the number of patients, and, if possible, conducting genetic and radiosensitivity analyses as well as tissue response monitoring, if applicable.

## Supplementary Information

Below is the link to the electronic supplementary material.

Supplementary file1 (DOCX 4343 KB)
