# Supplementary material for: The DNA damage response to radiological imaging: from ROS and γH2AX foci induction to gene expression responses in vivo
Source: Radiat Environ Biophys. 2023 Jun 19;62(3):371–93. doi: 10.1007/s00411-023-01033-4 (PMC10356679; doi:10.1007/s00411-023-01033-4)
Supplement: Supplementary file 1 — Supplementary file1 (DOCX 4343 KB) [file 411_2023_1033_MOESM1_ESM.docx]

**Supplemental Figures and Tables**

**The DNA damage response to radiological imaging: from ROS and γH2AX foci induction to gene expression responses *in vivo***

Milagrosa López-Riego^1,*^, Magdalena Płódowska^2^, Milena Lis-Zajęcka^2^, Kamila Jezierska^2^, Sylwia Tetela^2^, Aneta Węgierek-Ciuk^2^, Daniel Sobota^3^, Janusz Braziewicz^3,4^, Lovisa Lundholm^1^, Halina Lisowska^2^, Andrzej Wojcik^1,2^

1. Centre for Radiation Protection Research, Department of Molecular Biosciences, The Wenner-Gren Institute, Stockholm University, Sweden
2. Department of Medical Biology, Institute of Biology, Jan Kochanowski University, Kielce, Poland
3. Department of Medical Physics, Institute of Biology, Jan Kochanowski University, Kielce, Poland
4. Department of Nuclear Medicine with Positron Emission Tomography (PET) Unit, Holy Cross Cancer Centre, Kielce, Poland

*: author for correspondence

E-mail: [milagrosa.lopezriego@su.se](mailto:milagrosa.lopezriego@su.se)

**
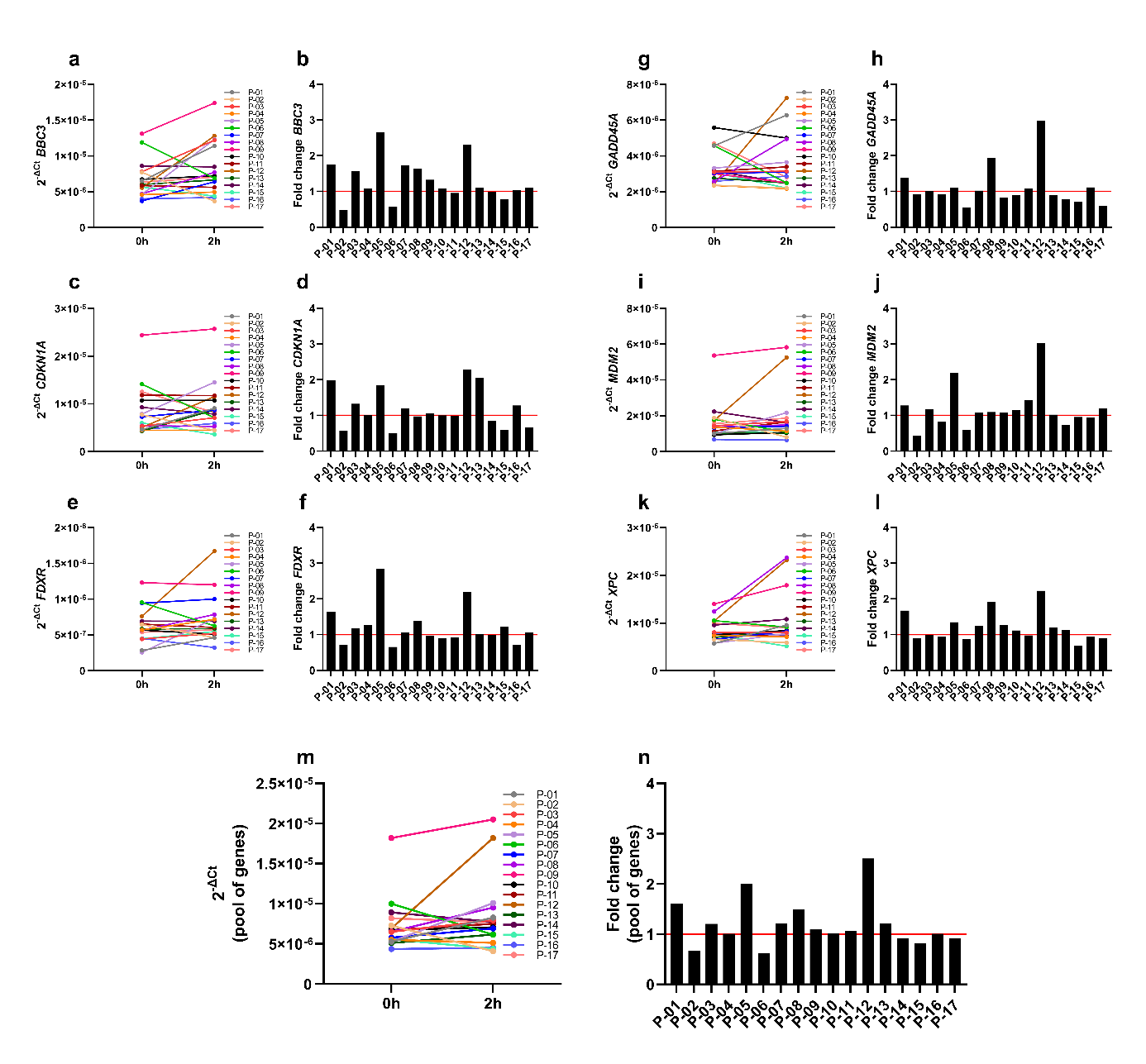
**

**Supplemental Fig. 1 Gene expression raw data (2^-ΔCt^) at 0 h and 2 h and corresponding fold changes in PET-CT blood samples.** Fold change: relative mRNA in 2 h-blood samples to control (0 h) after normalisation to housekeeping gene. Pool of genes: average of fold changes of the 6-panel of genes (*BBC3*, *CDKN1A*, *FDXR*, *GADD45A*, *MDM2*, and *XPC*) per patient. Each colour (panels A, C, E, G, I, K, and M) and bar (panels B, D, F, H, J, L, and N) represents one PET (P) patient. A red horizontal line at Y=1 represents a fold change equivalent to control values in panels B, D, F, H, J, L, and N


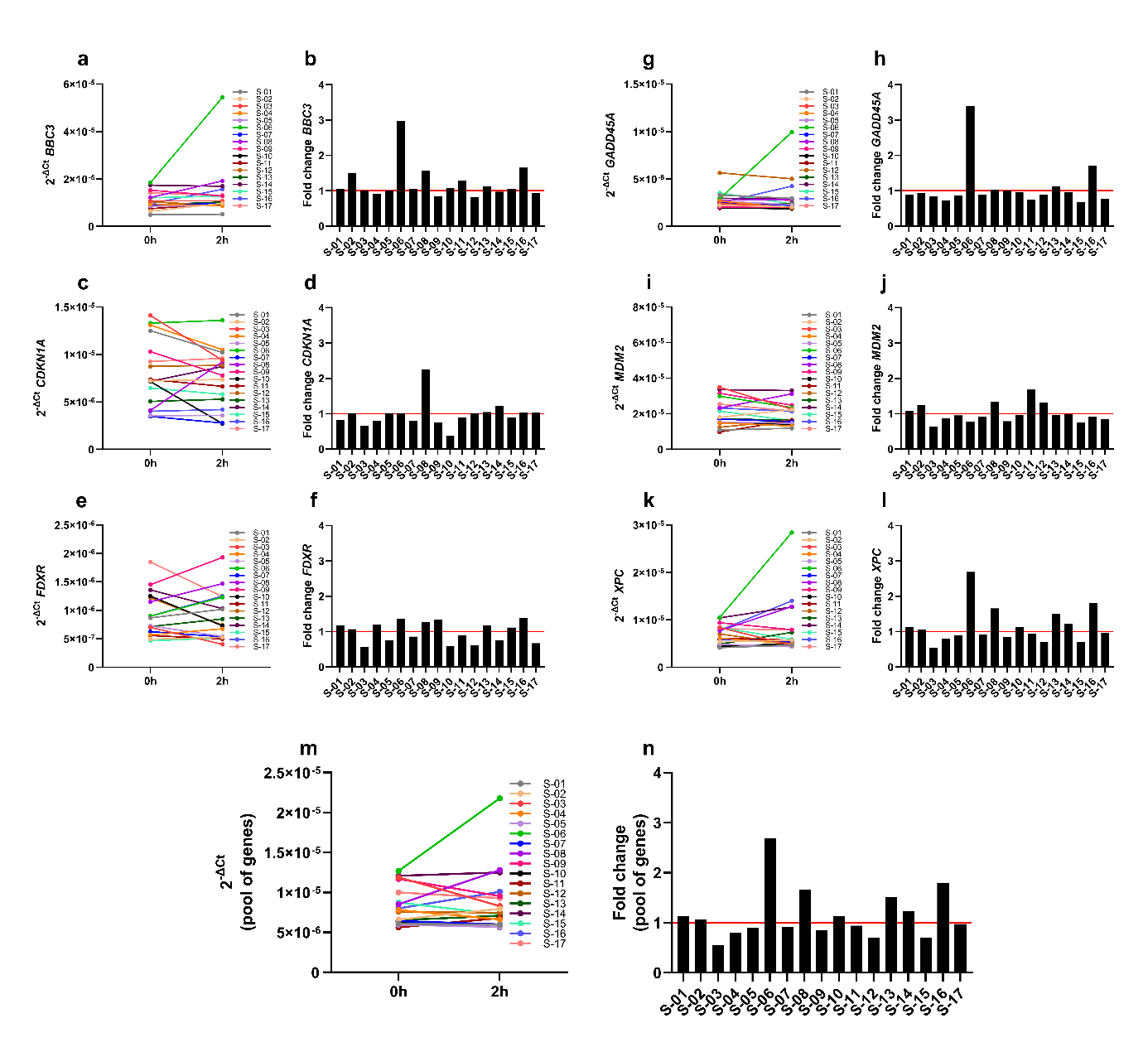


**Supplemental Fig. 2 Gene expression raw data (2^-ΔCt^) at 0 h and 2 h and corresponding fold changes in scintigraphy blood samples.** Fold change: relative mRNA in 2 h-blood samples to control (0 h) after normalisation to housekeeping gene. Pool of genes: average of fold changes of the 6-panel of genes (*BBC3*, *CDKN1A*, *FDXR*, *GADD45A*, *MDM2*, and *XPC*) per patient. Each colour (panels A, C, E, G, I, K, and M) and bar (panels B, D, F, H, J, L, and N) represents one patient scintigraphy (S) patient. A red horizontal line at Y=1 represents a fold change equivalent to control values in panels B, D, F, H, J, L, and N


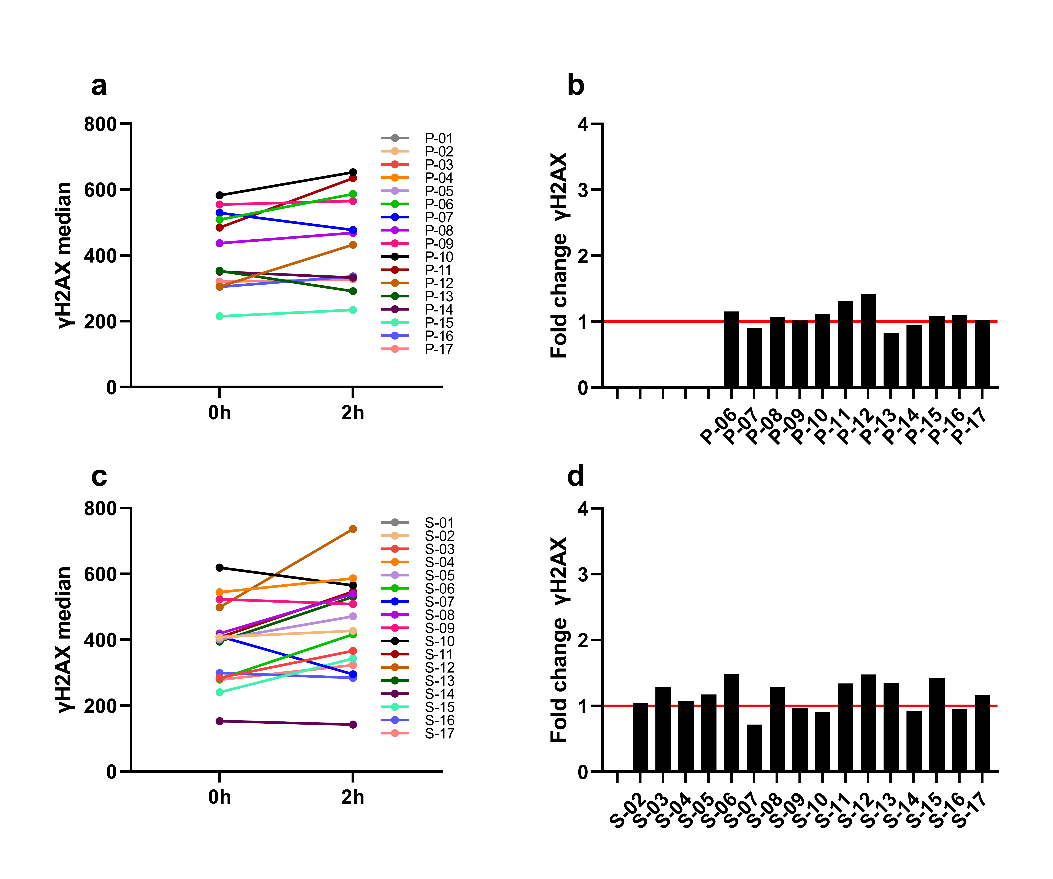


**Supplemental Fig. 3 Raw median γH2AX signal at 0 h and 2 h and corresponding fold change in PET-CT and scintigraphy blood samples relative to control.** A and B: PET (P) patients. C and D: scintigraphy (S) patients. Each colour (panels A and C) and bar (panels B and D) represents one patient. A red horizontal line at Y=1 represents a fold change equivalent to control values in panels B and D


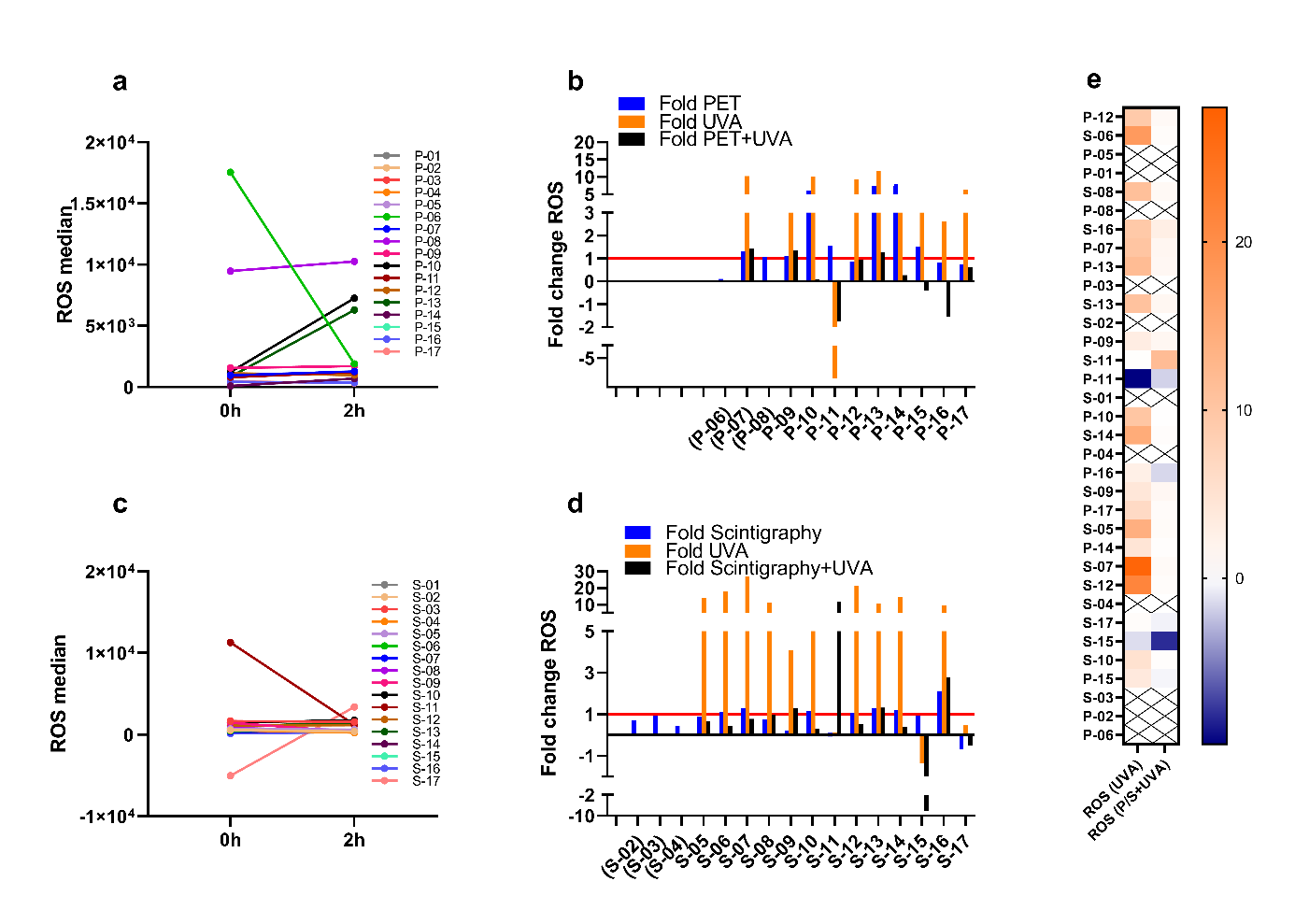


**Supplemental Fig. 4 Raw ROS median values at 0 h and 2 h based on the 2', 7' – Dichlorofluorescin diacetate (DCFDA) test and corresponding fold change of median oxidative stress in PET and scintigraphy blood samples relative to control.** A and B: PET (P) patients. C and D: scintigraphy (S) patients. Each colour (panels A and C) and bar (panels B and D) represents one patient. A red horizontal line at Y=1 represents a fold change equivalent to control values in panels B and D. Patients for which some of the results are missing (as shown in panel E) are indicated in brackets in panels B and D. Panel E: heatmap of Fold UVA and fold PET or Scintigraphy + UVA (P/S +UVA) observed for all P and S patients according to the scale shown on the right. Fold UVA: 0 h-blood samples exposed to UVA normalised to 0 h-blood samples controls. Fold PET (B) or scintigraphy (D): results of blood samples at 2 h after procedure normalised to samples at 0 h. Fold PET (B) or scintigraphy (panel D) + UVA: 2 h-blood samples exposed to UVA normalised to 0 h-blood samples exposed to UVA


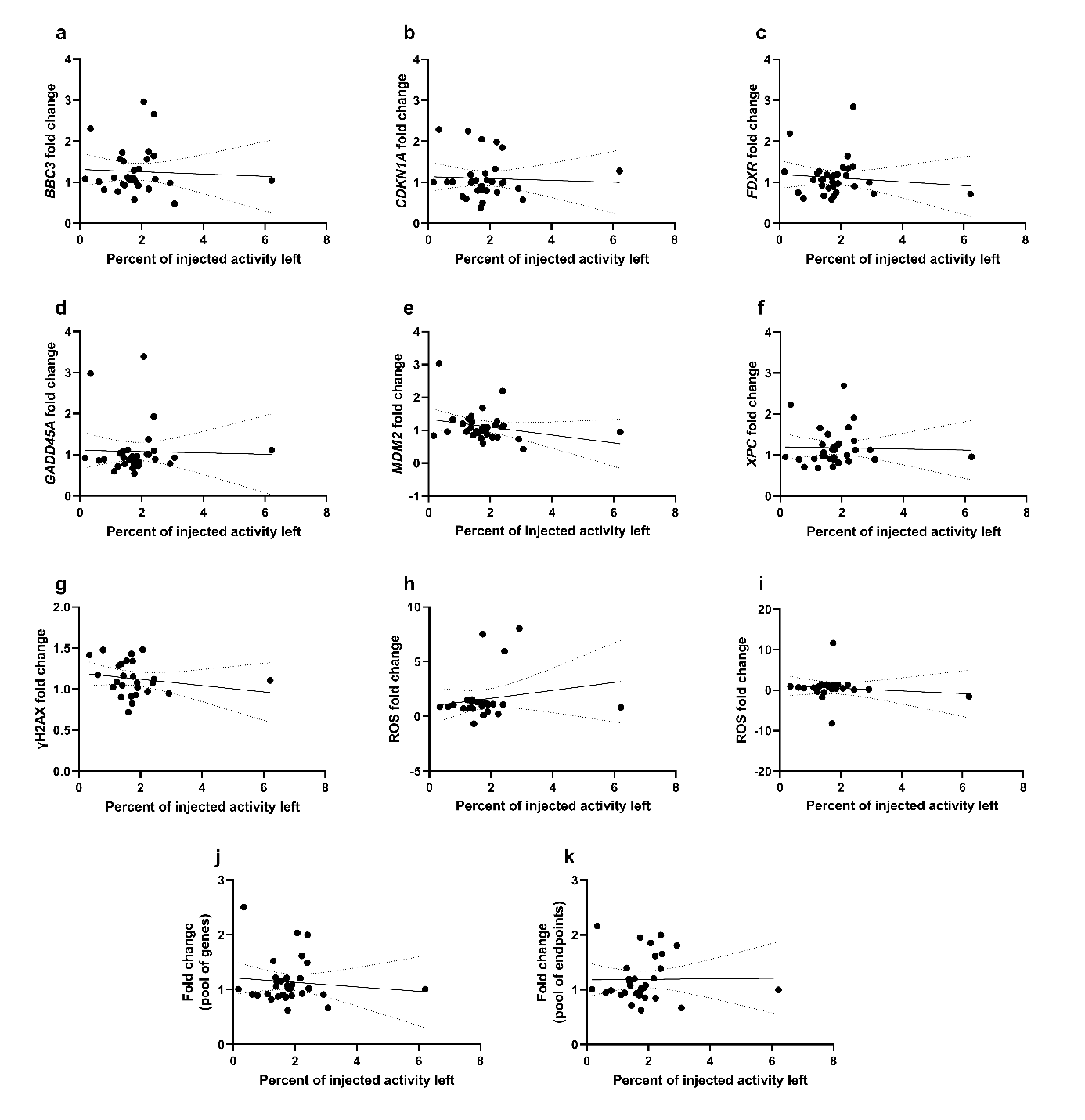


**Supplemental Fig. 5 Correlation of percent of activity left with fold change results from each endpoint considering the pool of all patients.** A-F: gene expression fold changes for *BBC3* (A), *CDKN1A* (B), *FDXR* (C), *GADD45A* (D), *MDM2* (E), and *XPC* (F). G: γH2AX fold change. H-I: ROS fold changes in blood samples 2 h after PET (P) or scintigraphy (S) procedure as compared to control samples at 0 h (panel H) or ROS fold changes in blood samples 2 h after PET (P) or scintigraphy (S) procedure and additional UVA exposure as compared to control samples at 0 h exposed to UVA (panel I). J: average fold change per patient for the pool of genes. K: average fold change per patient for the pool of endpoints (excluding ROS UVA). Each symbol represents one individual. Linear regressions (Supplemental Table 5) are represented with a black solid bar and 95% confidence interval are represented with dotted black bands

**
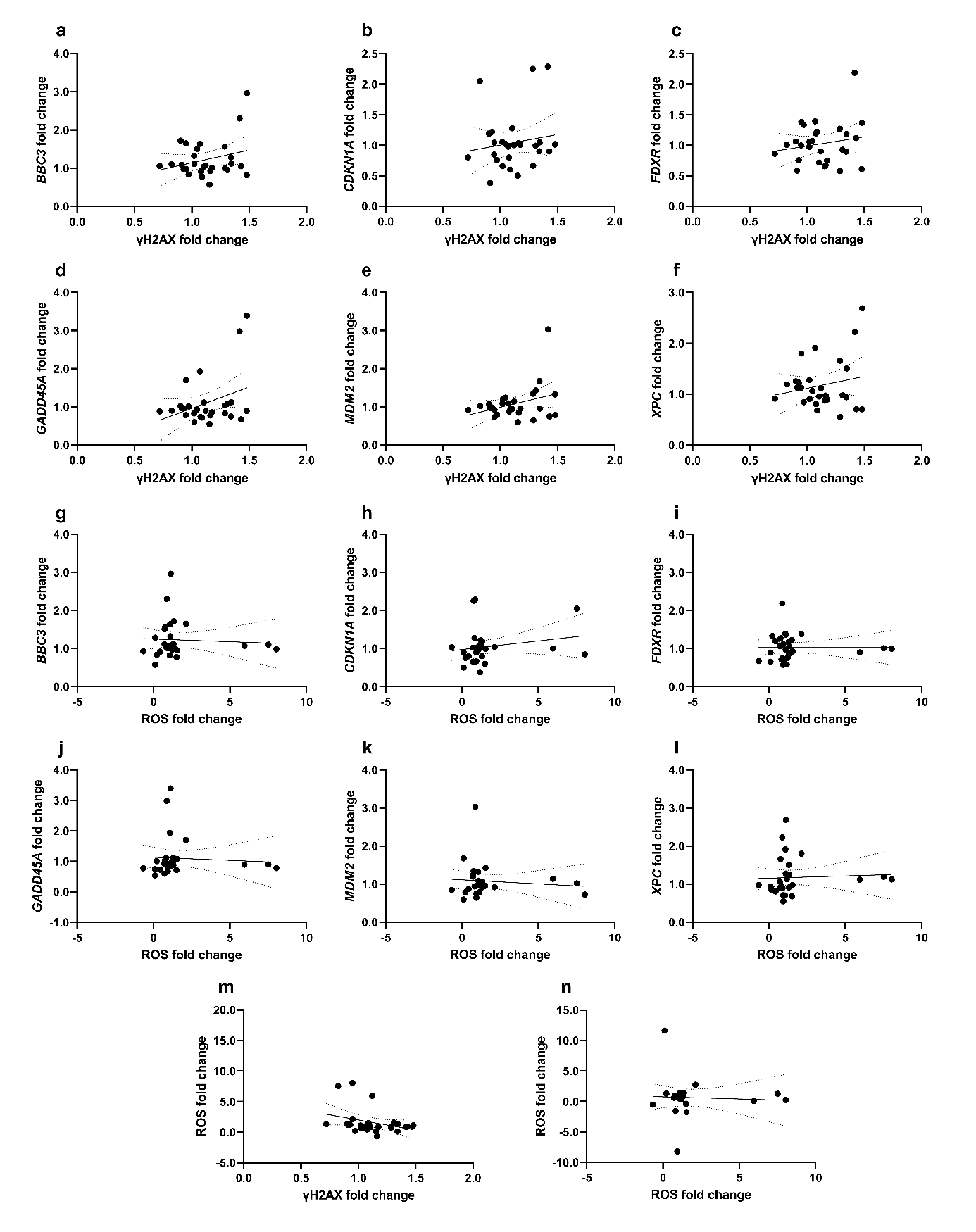
Supplemental Fig. 6 Correlations between endpoints for fold change values.** Gene expression fold change correlation with γH2AX fold change (panels A-F) or with ROS fold change (panels G-L) and ROS fold change correlation with γH2AX fold change (panel M) or with ROS after additional UVA exposure (panel N). Each symbol represents one individual. Linear regressions (Supplemental Table 6) are represented with a black solid bar and 95% confidence interval are represented with dotted black bands


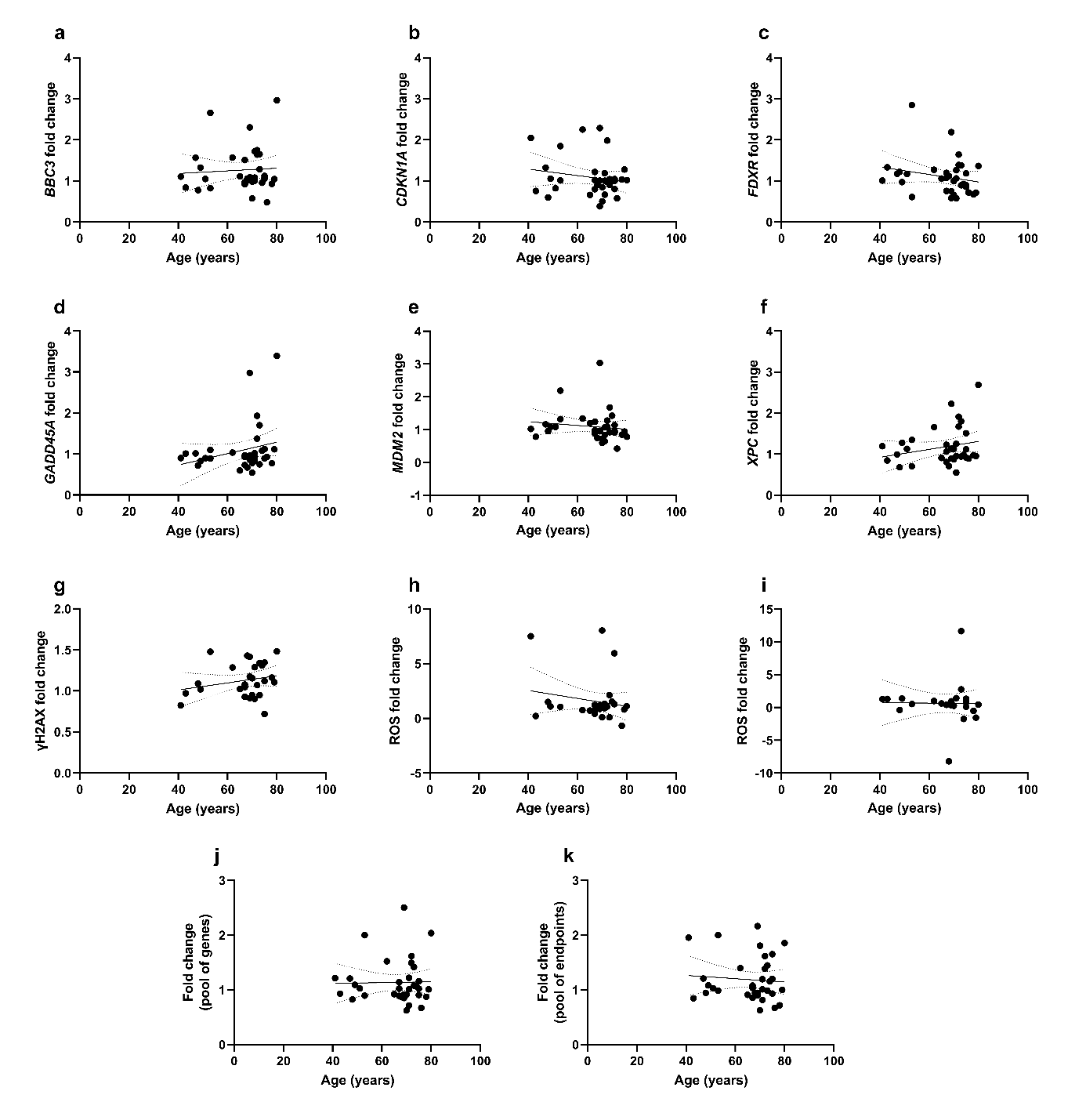


**Supplemental Fig. 7 Correlation of patients age (years) with fold change results from each endpoint considering the pool of all patients.** A-F: gene expression fold changes for *BBC3* (A), *CDKN1A* (B), *FDXR* (C), *GADD45A* (D), *MDM2* (E), and *XPC* (F). G: γH2AX fold change. H-I: ROS fold changes in blood samples 2 h after PET (P) or scintigraphy (S) procedure as compared to control samples at 0 h (panel H) ROS fold changes in blood samples 2 h after PET (P) or scintigraphy (S) procedure and additional UVA exposure as compared to control samples at 0 h exposed to UVA (panel I). J: average fold change per patient for the pool of genes. K: average fold change per patient for the pool of endpoints (excluding ROS UVA). Each symbol represents one individual. Linear regressions (Supplemental Table 7) are represented with a black solid bar and 95% confidence interval are represented with dotted black bands

**
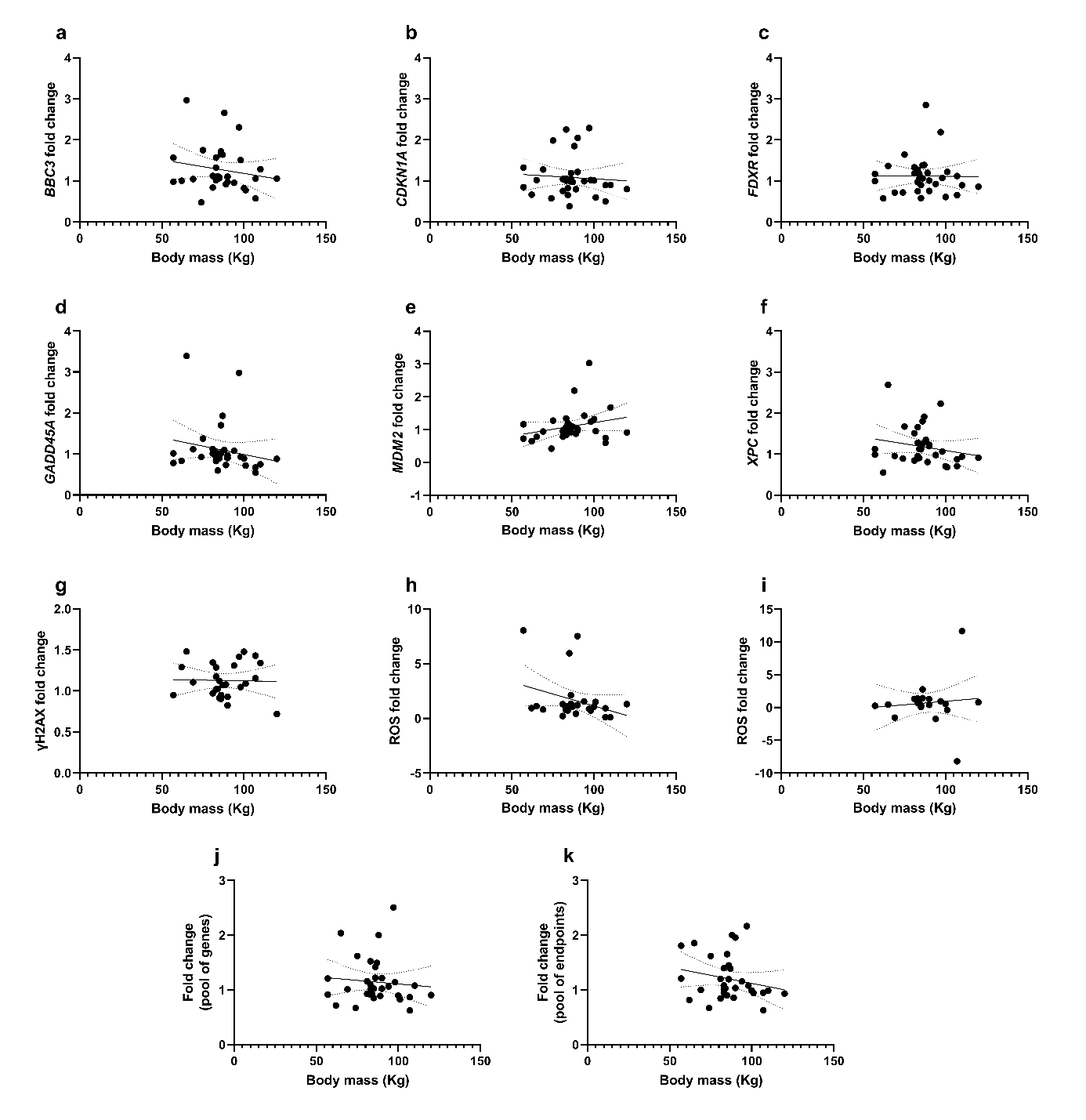
Supplemental Fig. 8 Correlation of patients body mass (Kg) with fold change results from each endpoint considering the pool of all patients.** A-F: gene expression fold changes for *BBC3* (A), *CDKN1A* (B), *FDXR* (C), *GADD45A* (D), *MDM2* (E), and *XPC* (F). G: γH2AX fold change. H-I: ROS fold changes in blood samples 2 h after PET (P) or scintigraphy (S) procedure as compared to control samples at 0 h (panel H) ROS fold changes in blood samples 2 h after PET (P) or scintigraphy (S) procedure and additional UVA exposure as compared to control samples at 0 h exposed to UVA (panel I). J: average fold change per patient for the pool of genes. K: average fold change per patient for the pool of endpoints (excluding ROS UVA). Each symbol represents one individual. Linear regressions (Supplemental Table 8) are represented with a black solid bar and 95% confidence interval are represented with dotted black bands

**
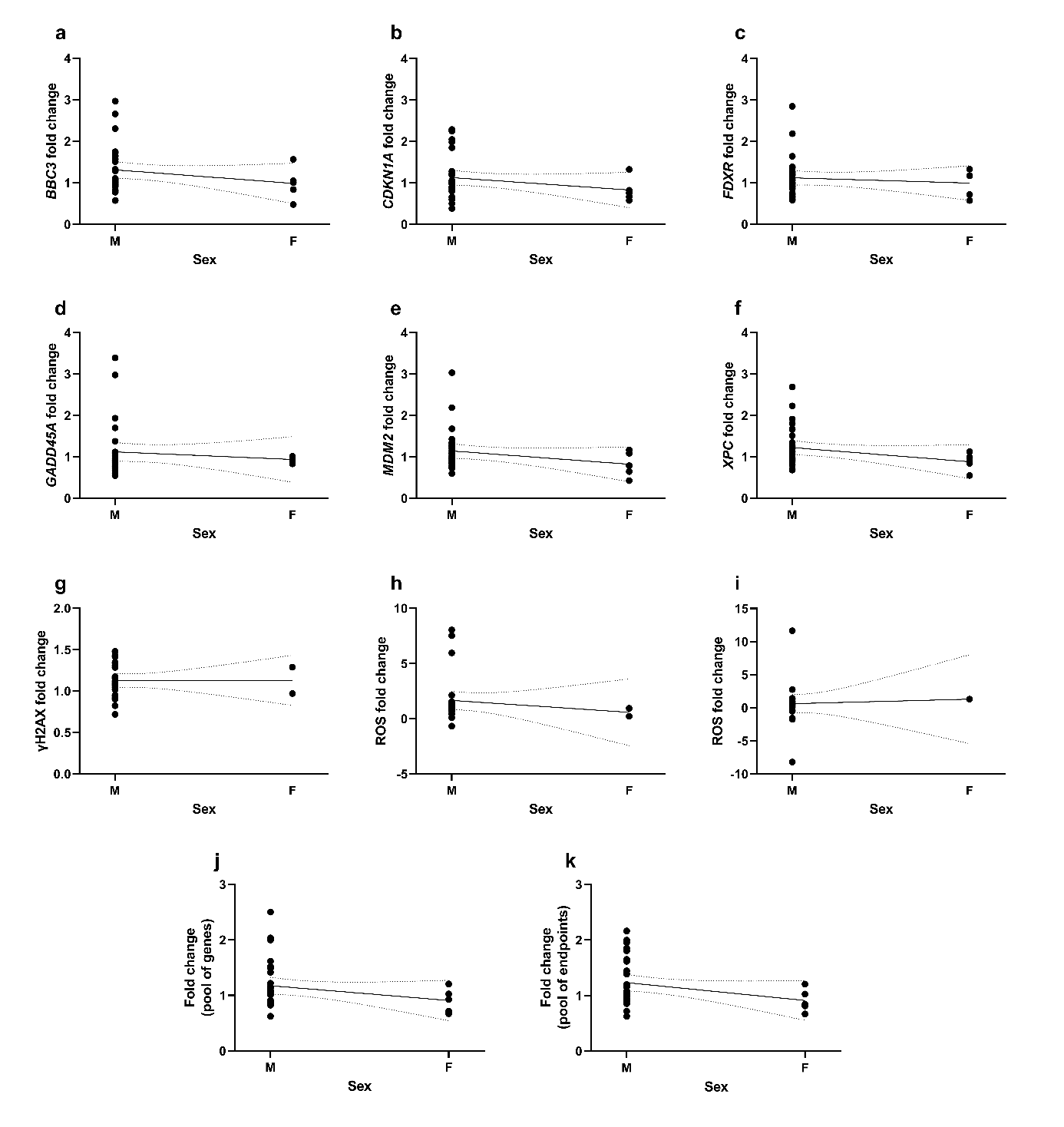
Supplemental Fig. 9 Correlation of patients sex with fold change results from each endpoint considering the pool of all patients.** M: males, F: females. A-F: gene expression fold changes for *BBC3* (A), *CDKN1A* (B), *FDXR* (C), *GADD45A* (D), *MDM2* (E), and *XPC* (F). G: γH2AX fold change. H-I: ROS fold changes in blood samples 2 h after PET (P) or scintigraphy (S) procedure as compared to control samples at 0 h (panel H) ROS fold changes in blood samples 2 h after PET (P) or scintigraphy (S) procedure and additional UVA exposure as compared to control samples at 0 h exposed to UVA (panel I). J: average fold change per patient for the pool of genes. K: average fold change per patient for the pool of endpoints (excluding ROS UVA). Each symbol represents one individual. Linear regressions (Supplemental Table 9) are represented with a black solid bar and 95% confidence interval are represented with dotted black bands


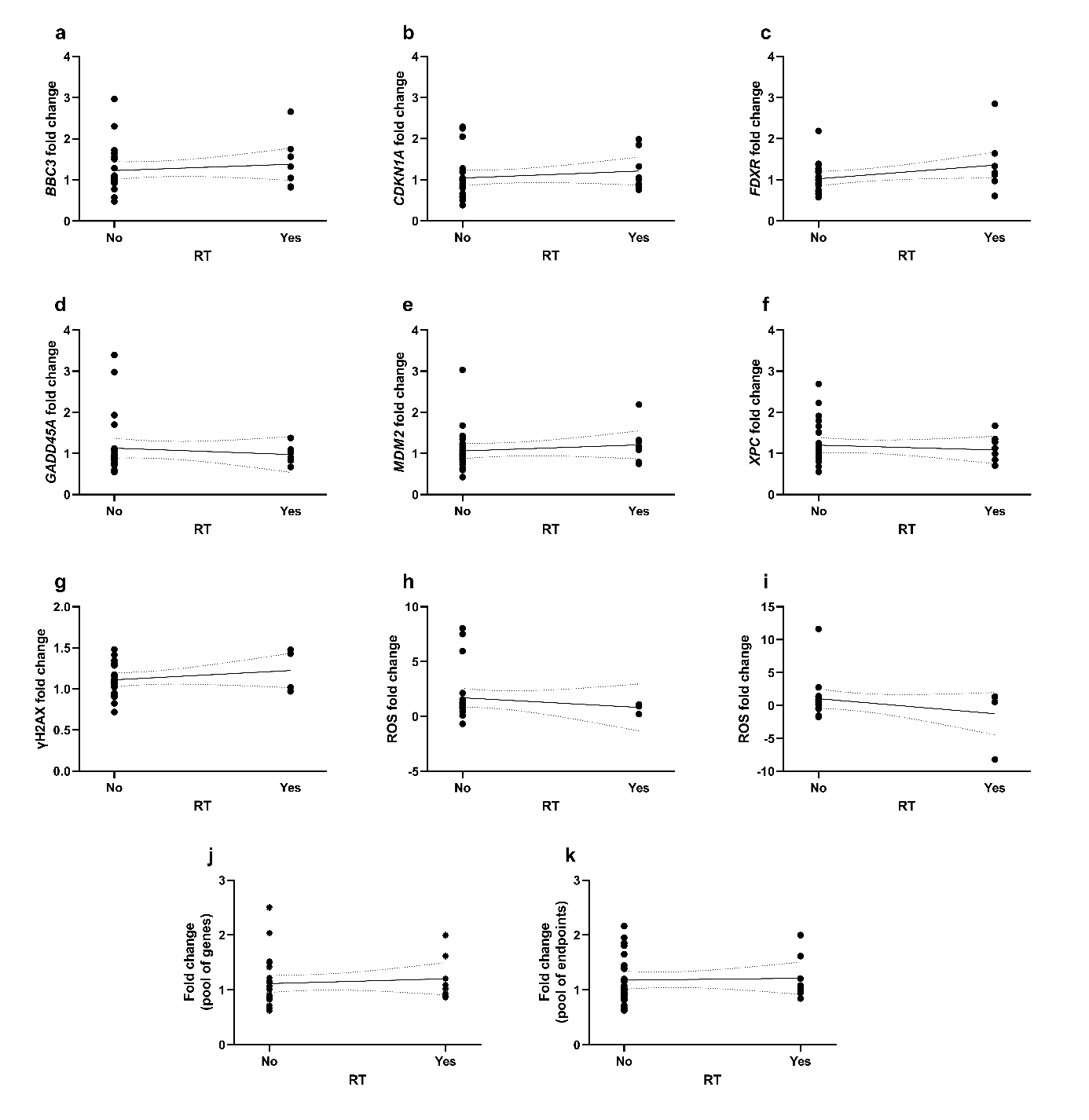


**Supplemental Fig. 10 Correlation of previous radiotherapy (RT) treatment record with fold change results from each endpoint considering the pool of all patients.** A-F: gene expression fold changes for *BBC3* (A), *CDKN1A* (B), *FDXR* (C), *GADD45A* (D), *MDM2* (E), and *XPC* (F). G: γH2AX fold change. H-I: ROS fold changes in blood samples 2 h after PET (P) or scintigraphy (S) procedure as compared to control samples at 0 h (panel H) ROS fold changes in blood samples 2 h after PET (P) or scintigraphy (S) procedure and additional UVA exposure as compared to control samples at 0 h exposed to UVA (panel I). Each symbol represents one individual. Linear regressions (Supplemental Table 10) are represented with a black solid bar and 95% confidence interval are represented with dotted black bands

**
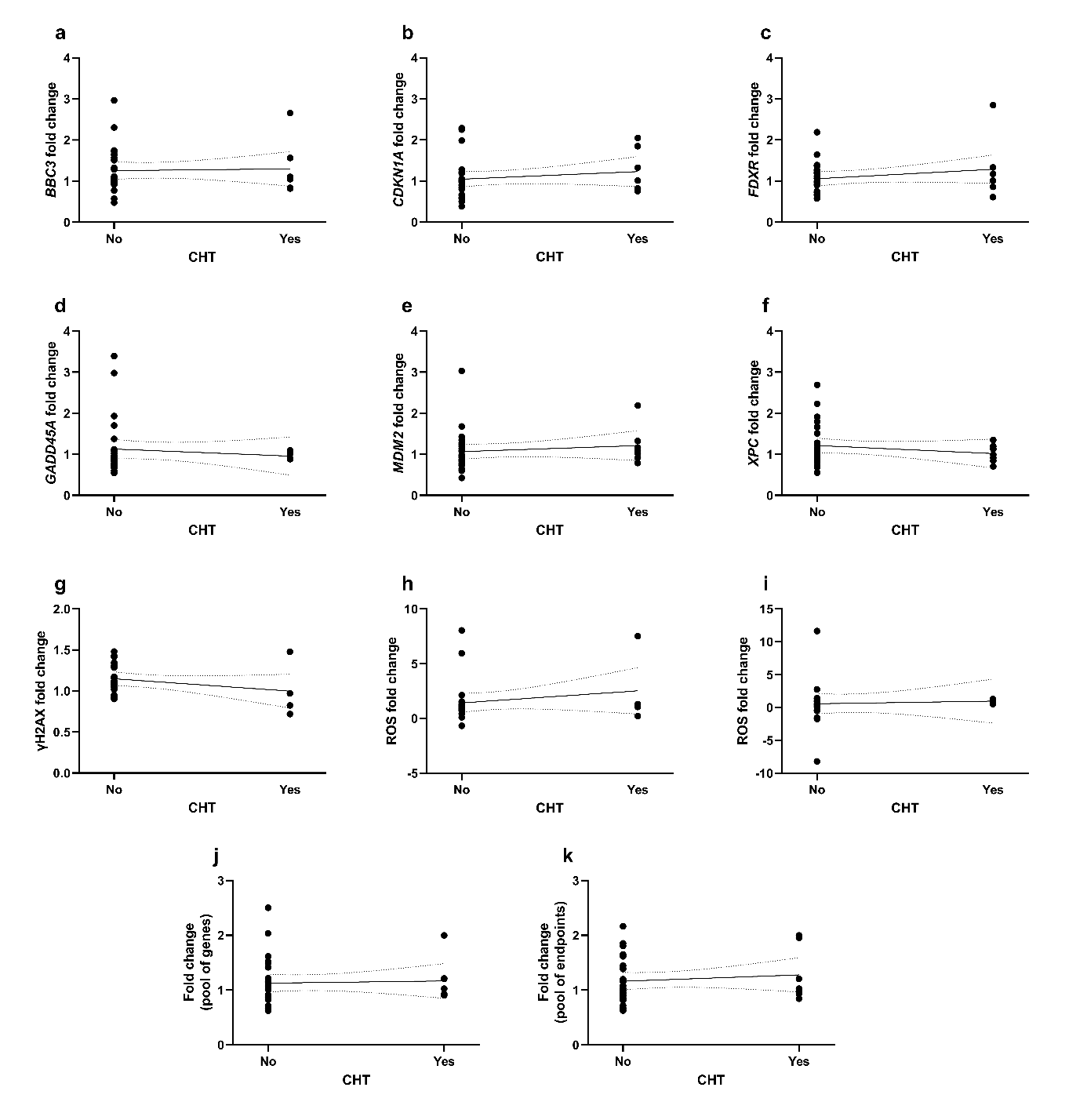
Supplemental Fig. 11 Correlation of previous chemotherapy (CHT) treatment record with fold change results from each endpoint considering the pool of all patients.** A-F: gene expression fold changes for *BBC3* (A), *CDKN1A* (B), *FDXR* (C), *GADD45A* (D), *MDM2* (E), and *XPC* (F). G: γH2AX fold change. H-I: ROS fold changes in blood samples 2 h after PET (P) or scintigraphy (S) procedure as compared to control samples at 0 h (panel H) ROS fold changes in blood samples 2 h after PET (P) or scintigraphy (S) procedure and additional UVA exposure as compared to control samples at 0 h exposed to UVA (panel I). Each symbol represents one individual. Linear regressions (Supplemental Table 11) are represented with a black solid bar and 95% confidence interval are represented with dotted black bands

**
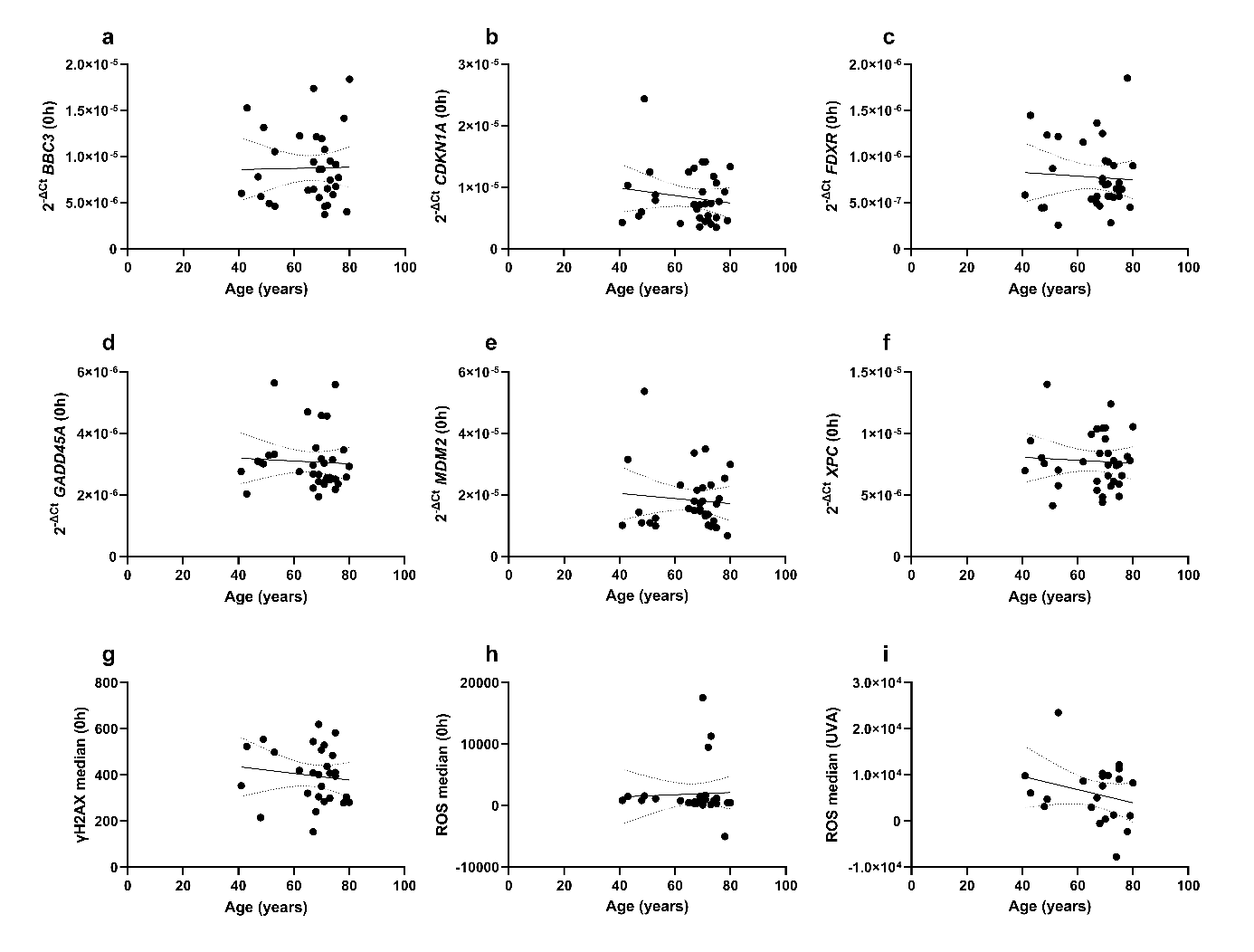
Supplemental Fig. 12 Correlation of patients age (years) with raw results from each endpoint at 0 h considering the pool of all patients.** A-F: 2^-ΔCt^ values for *BBC3* (A), *CDKN1A* (B), *FDXR* (C), *GADD45A* (D), *MDM2* (E), and *XPC* (F). G: γH2AX median. H-I: ROS median in blood samples at 0 h (panel H) or after UVA exposure (panel I). Each symbol represents one individual. Linear regressions (Supplemental Table 13) are represented with a black solid bar and 95% confidence interval are represented with dotted black bands


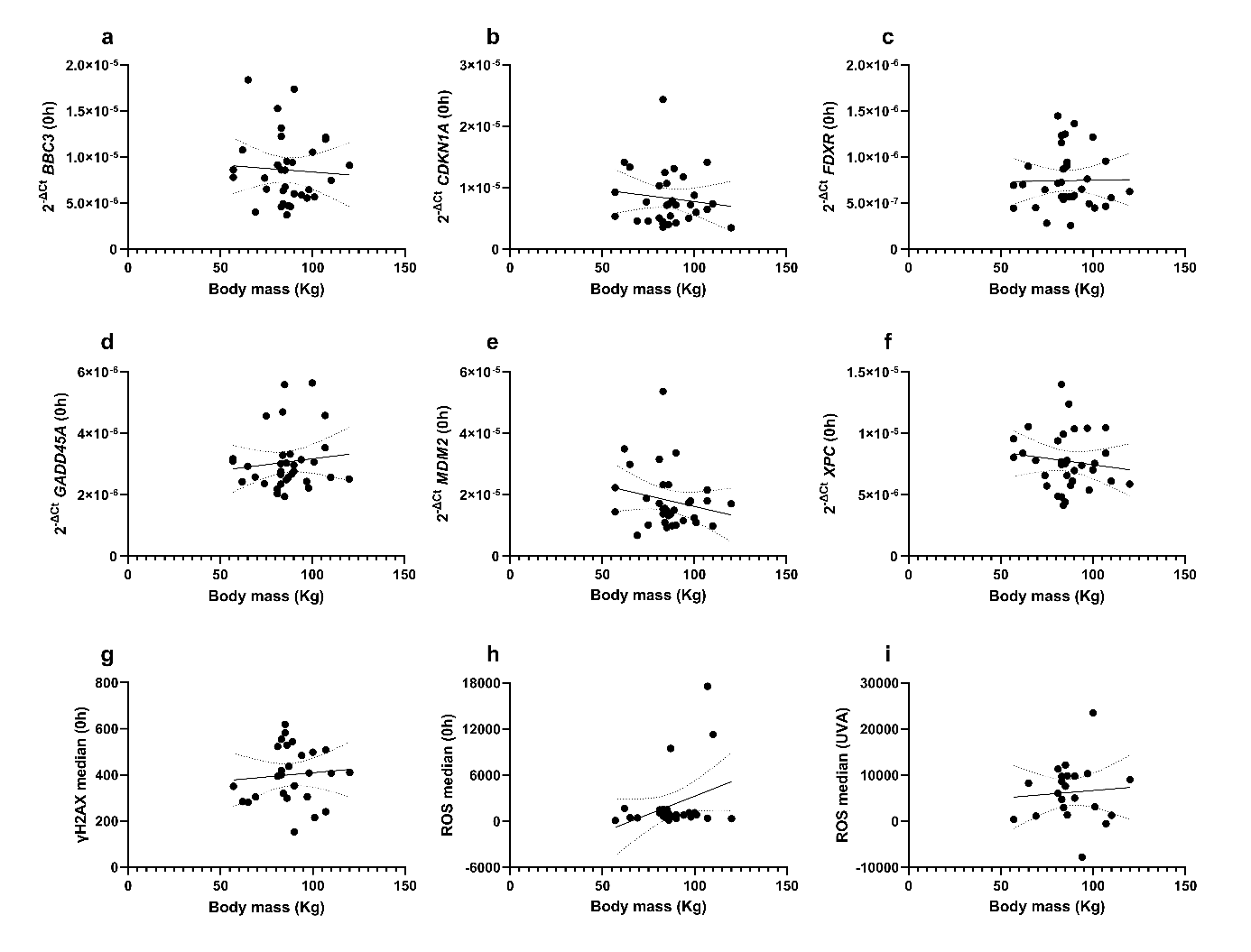


**Supplemental Fig. 13 Correlation of patients body mass (kg) with raw results from each endpoint at 0 h considering the pool of all patients.** A-F: 2^-ΔCt^ values for *BBC3* (A), *CDKN1A* (B), *FDXR* (C), *GADD45A* (D), *MDM2* (E), and *XPC* (F). G: γH2AX median. H-I: ROS median in blood samples at 0 h (panel H) or after UVA exposure (panel I). Each symbol represents one individual. Linear regressions (Supplemental Table 14) are represented with a black solid bar and 95% confidence interval are represented with dotted black bands


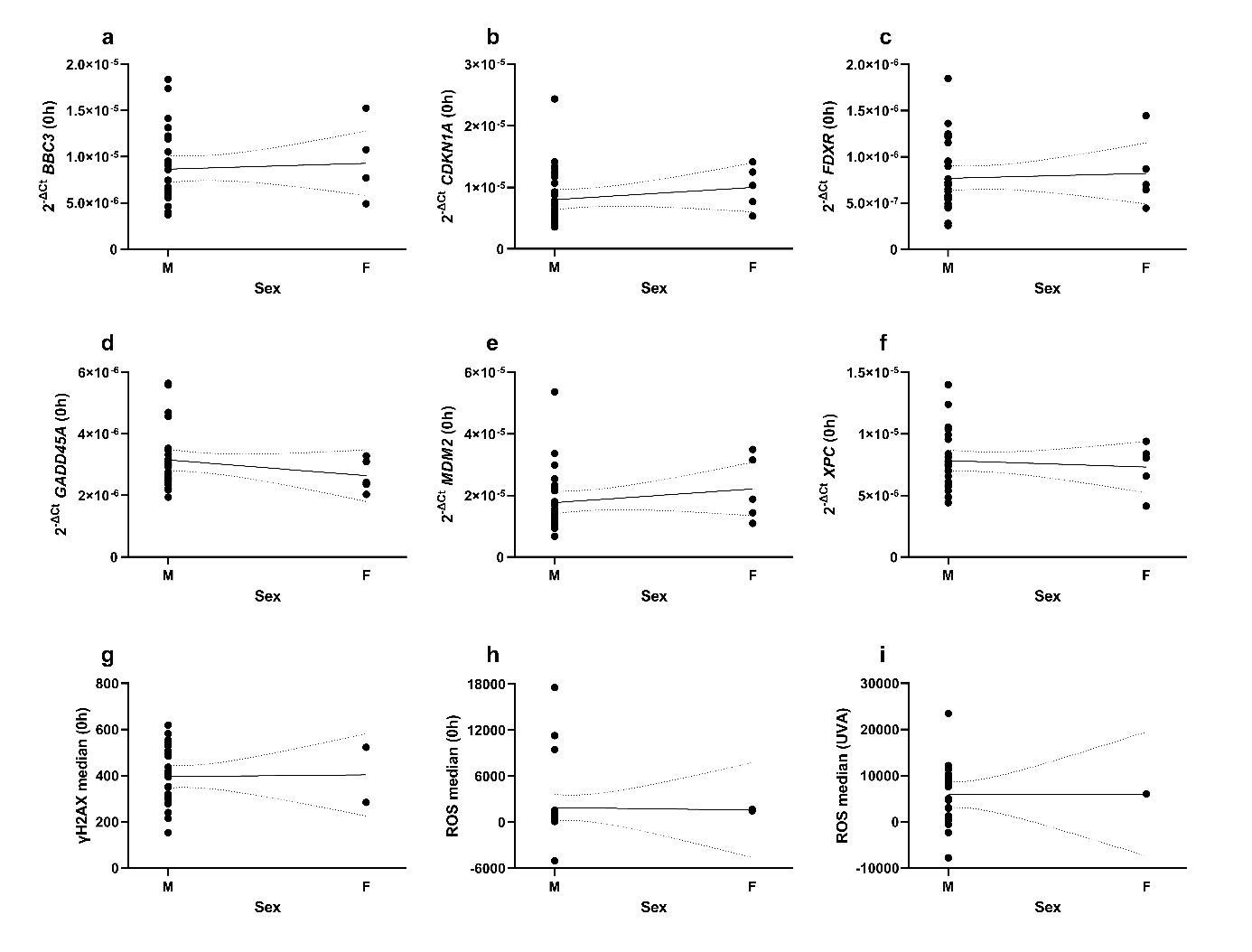


**Supplemental Fig. 14 Correlation of patients sex with raw results from each endpoint at 0 h considering the pool of all patients.** M: males, F: females. A-F: 2^-ΔCt^ values for *BBC3* (A), *CDKN1A* (B), *FDXR* (C), *GADD45A* (D), *MDM2* (E), and *XPC* (F). G: γH2AX median. H-I: ROS median in blood samples at 0 h (panel H) or after UVA exposure (panel I). Each symbol represents one individual. Linear regressions (Supplemental Table 15) are represented with a black solid bar and 95% confidence interval are represented with dotted black bands


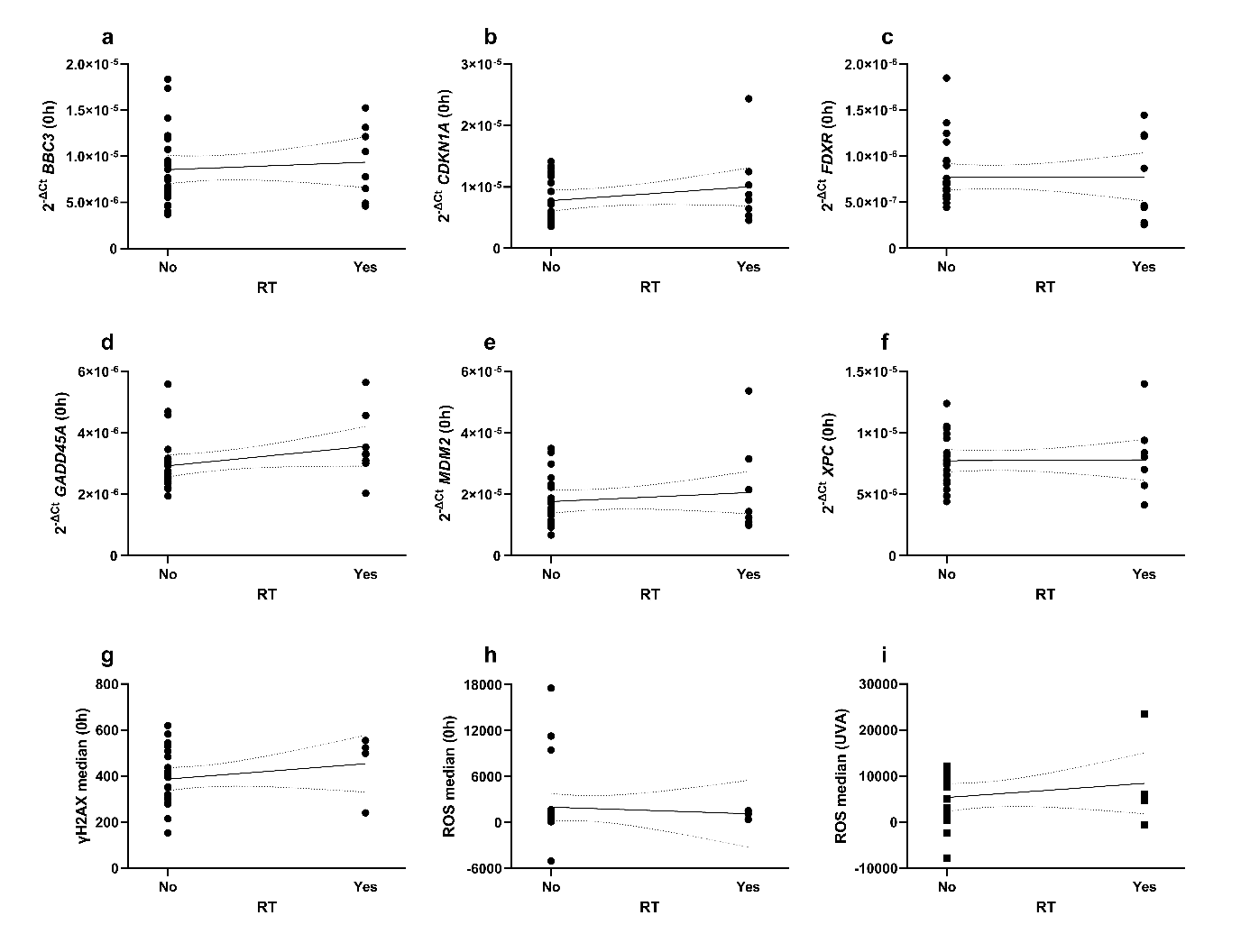


**Supplemental Fig. 15 Correlation of previous radiotherapy (RT) treatment record with raw results from each endpoint at 0 h considering the pool of all patients.** A-F: 2^-ΔCt^ values for *BBC3* (A), *CDKN1A* (B), *FDXR* (C), *GADD45A* (D), *MDM2* (E), and *XPC* (F). G: γH2AX median. H-I: ROS median in blood samples at 0 h (panel H) or after UVA exposure (panel I). Each symbol represents one individual. Linear regressions (Supplemental Table 16) are represented with a black solid bar and 95% confidence interval are represented with dotted black bands

**
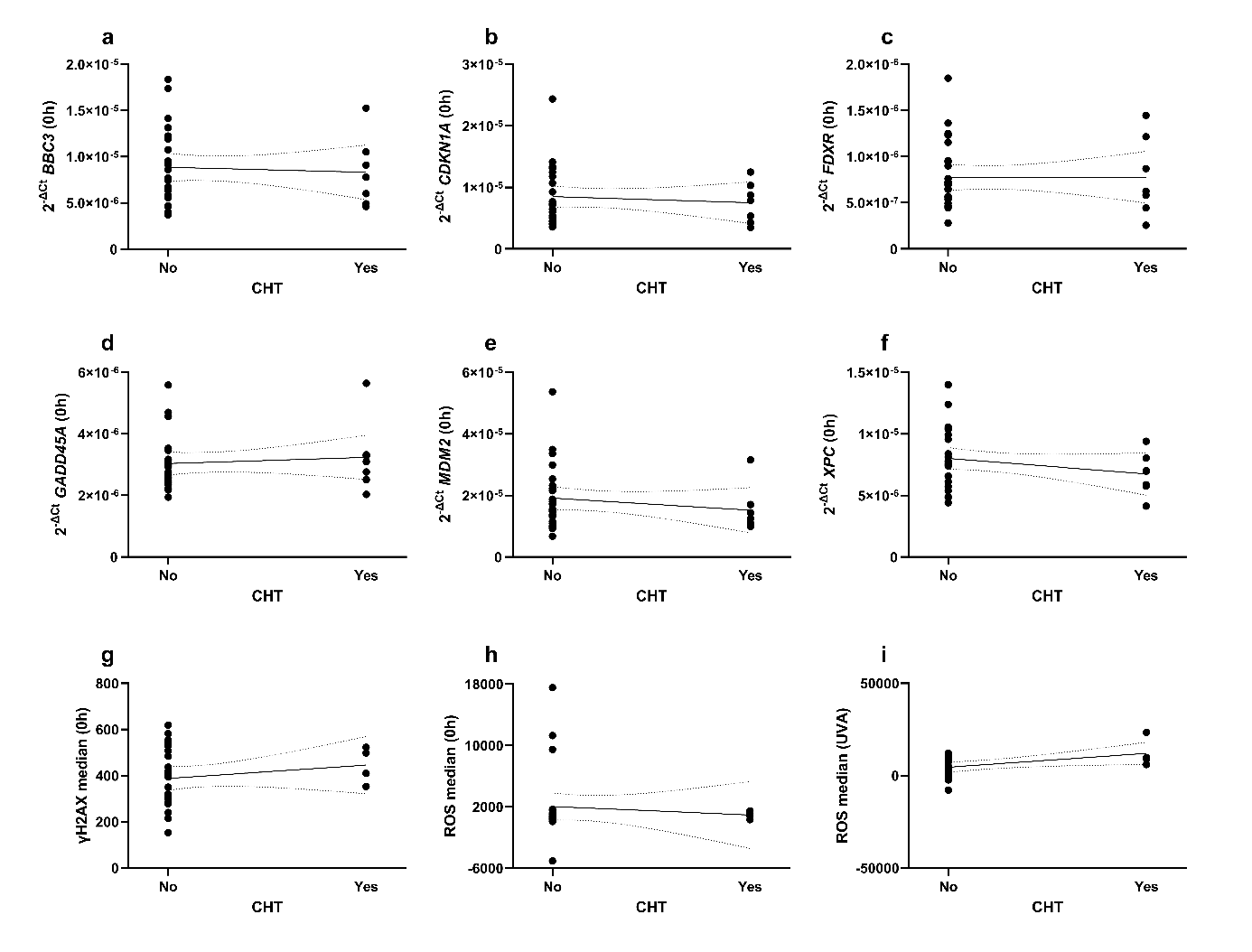
Supplemental Fig. 16 Correlation of previous chemotherapy (CHT) treatment record with raw results from each endpoint at 0 h considering the pool of all patients.** A-F: 2^-ΔCt^ values for *BBC3* (A), *CDKN1A* (B), *FDXR* (C), *GADD45A* (D), *MDM2* (E), and *XPC* (F). G: γH2AX median. H-I: ROS median in blood samples at 0 h (H) or after UVA exposure (I). Each symbol represents one individual. Linear regressions (Supplemental Table 17) are represented with a black solid bar and 95% confidence interval are represented with dotted black bands

**
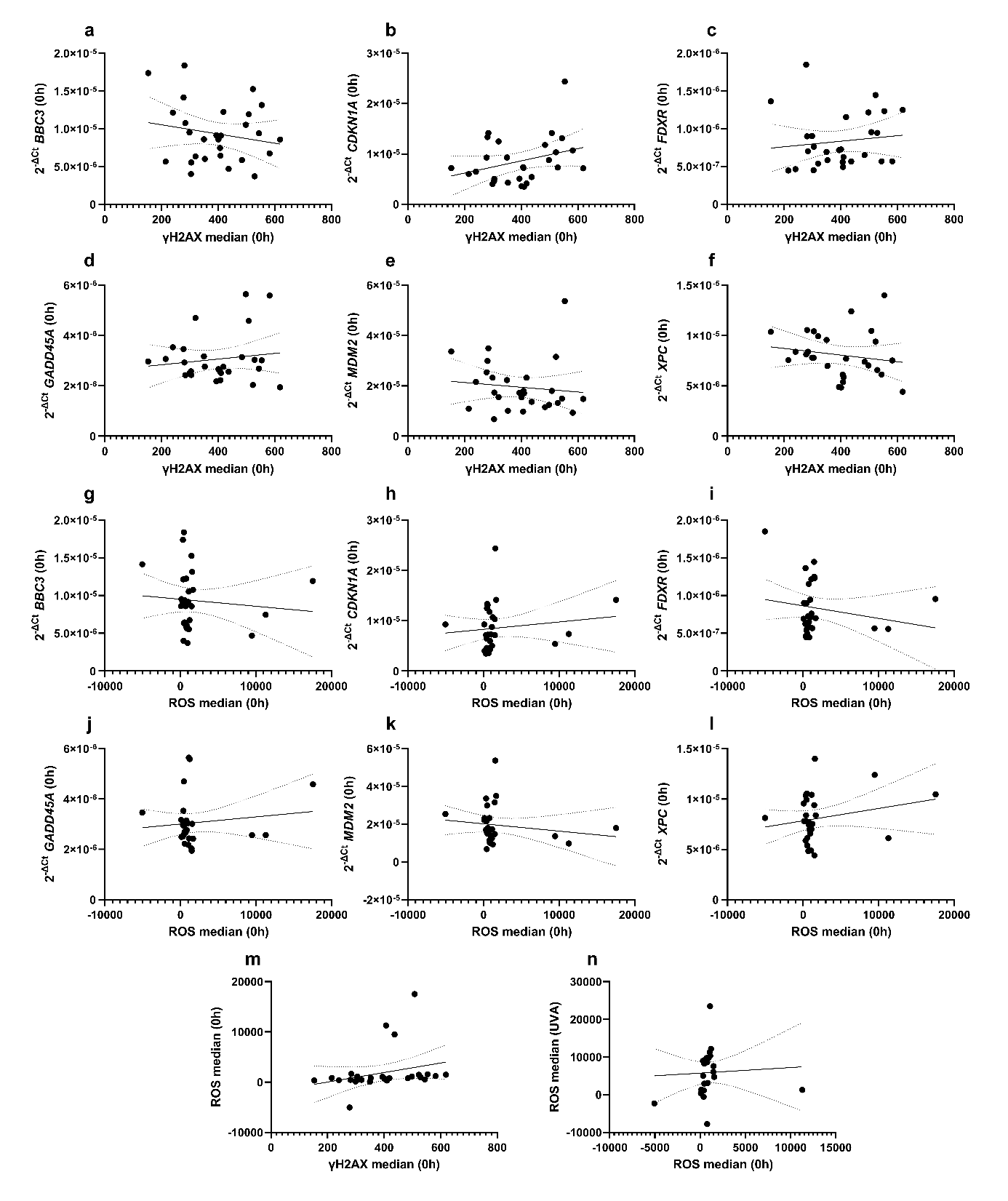
Supplemental Fig. 17 Correlations between endpoints for raw data at 0 h.** Gene expression 2^-ΔCt^ correlation with γH2AX median (A-F) or with ROS median (G-L) and ROS median correlation with γH2AX median (M) or with ROS median after UVA exposure (N). Each symbol represents one individual. Linear regressions (Supplemental Table 18) are represented with a black solid bar and 95% confidence interval are represented with dotted black bands

**
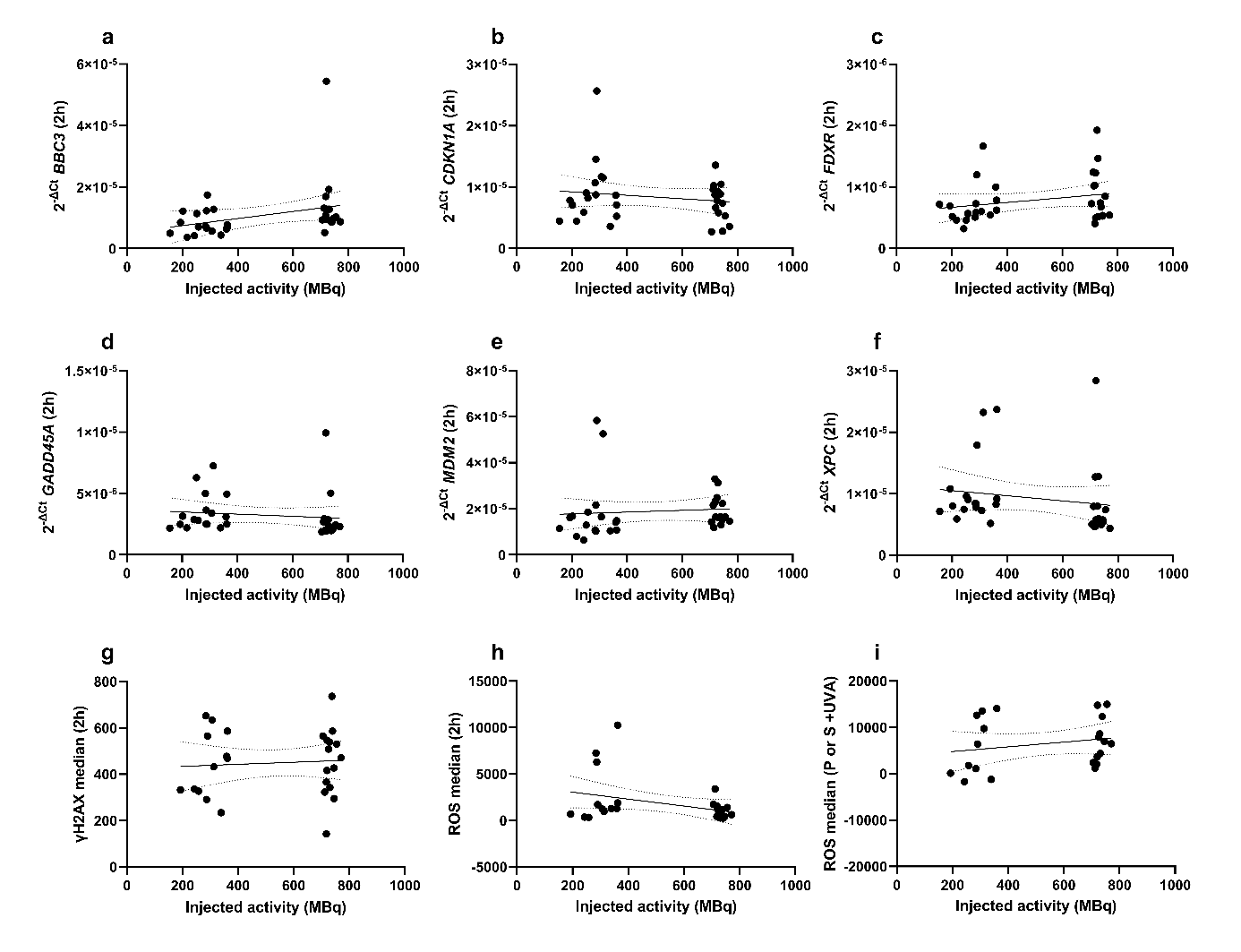
Supplemental Fig. 18 Correlation of injected activity (MBq) with raw results from each endpoint at 2 h considering the pool of all patients.** A-F: 2^-ΔCt^ values for *BBC3* (A), *CDKN1A* (B), *FDXR* (C), *GADD45A* (D), *MDM2* (E), and *XPC* (F). G: γH2AX median. H-I: ROS median in blood samples 2 h after PET (P) or scintigraphy (S) procedure (H) or after PET (P) or scintigraphy (S) procedure and additional UVA exposure (I). Each symbol represents one individual. Linear regressions (Supplemental Table 19) are represented with a black solid bar and 95% confidence interval are represented with dotted black bands

**
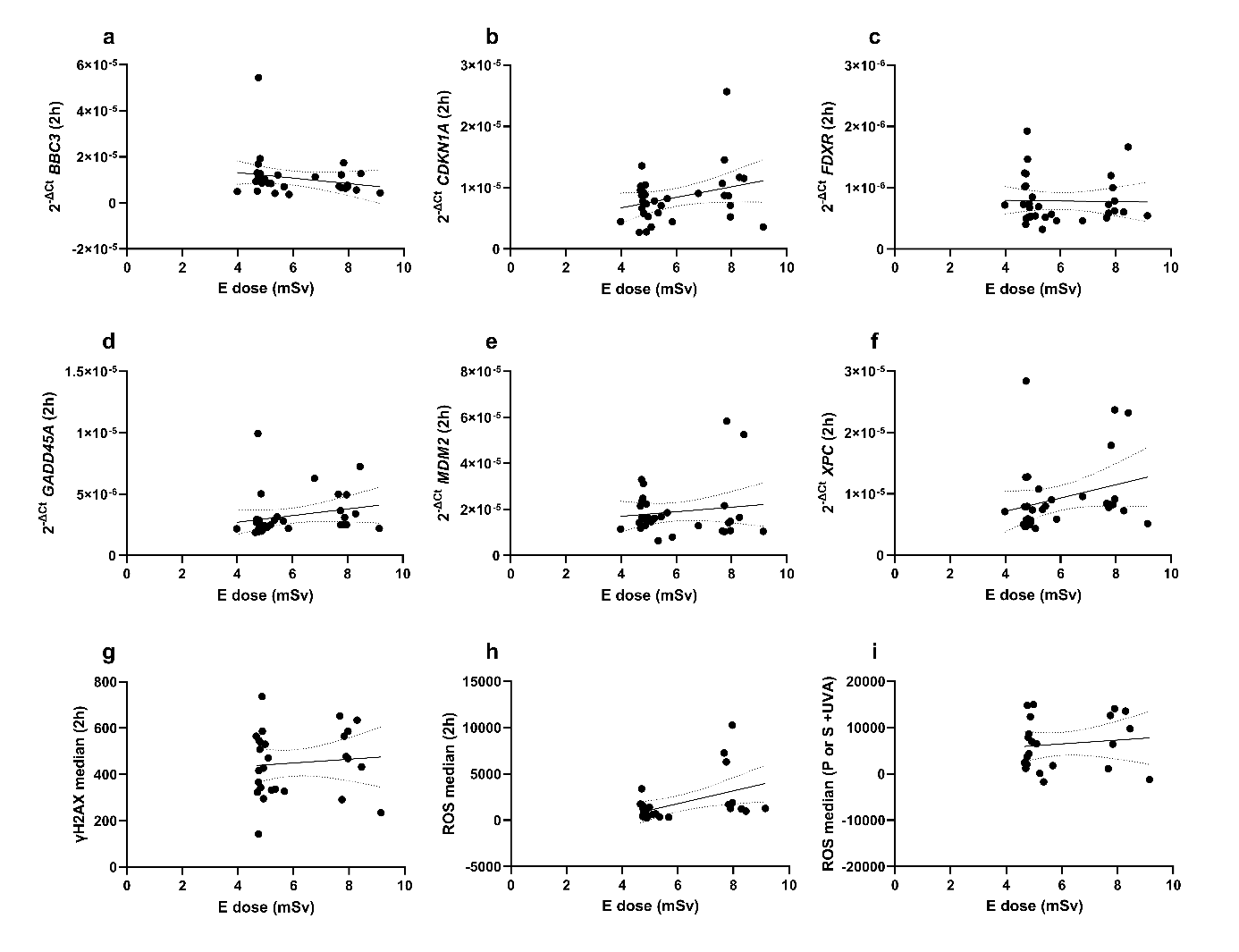
Supplemental Fig. 19 Correlation of effective (E) dose (mSv) with raw results from each endpoint at 2 h considering the pool of all patients.** A-F: 2^-ΔCt^ values for *BBC3* (A), *CDKN1A* (B), *FDXR* (C), *GADD45A* (D), *MDM2* (E), and *XPC* (F). G: γH2AX median. H-I: ROS median in blood samples 2 h after PET (P) or scintigraphy (S) procedure (H) or after PET (P) or scintigraphy (S) procedure and additional UVA exposure (I). Each symbol represents one individual. Linear regressions (Supplemental Table 20) are represented with a black solid bar and 95% confidence interval are represented with dotted black bands

**
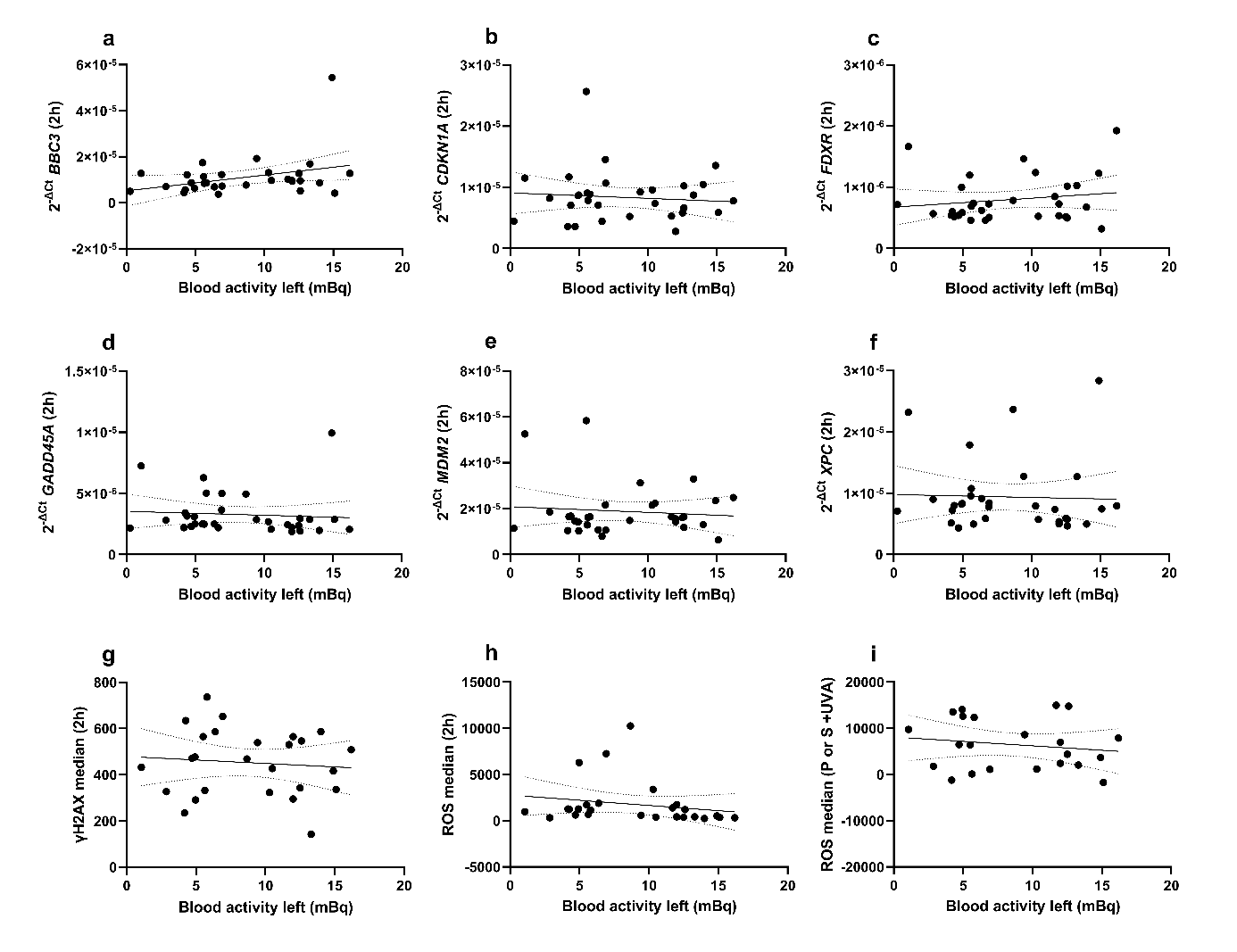
Supplemental Fig. 20 Correlation of blood activity left (mBq) with raw results from each endpoint at 2 h considering the pool of all patients.** A-F: 2^-ΔCt^ values for *BBC3* (A), *CDKN1A* (B), *FDXR* (C), *GADD45A* (D), *MDM2* (E), and *XPC* (F). G: γH2AX median. H-I: ROS median in blood samples 2 h after PET (P) or scintigraphy (S) procedure (H) or after PET (P) or scintigraphy (S) procedure and additional UVA exposure (I). Each symbol represents one individual. Linear regressions (Supplemental Table 21) are represented with a black solid bar and 95% confidence interval are represented with dotted black bands

**
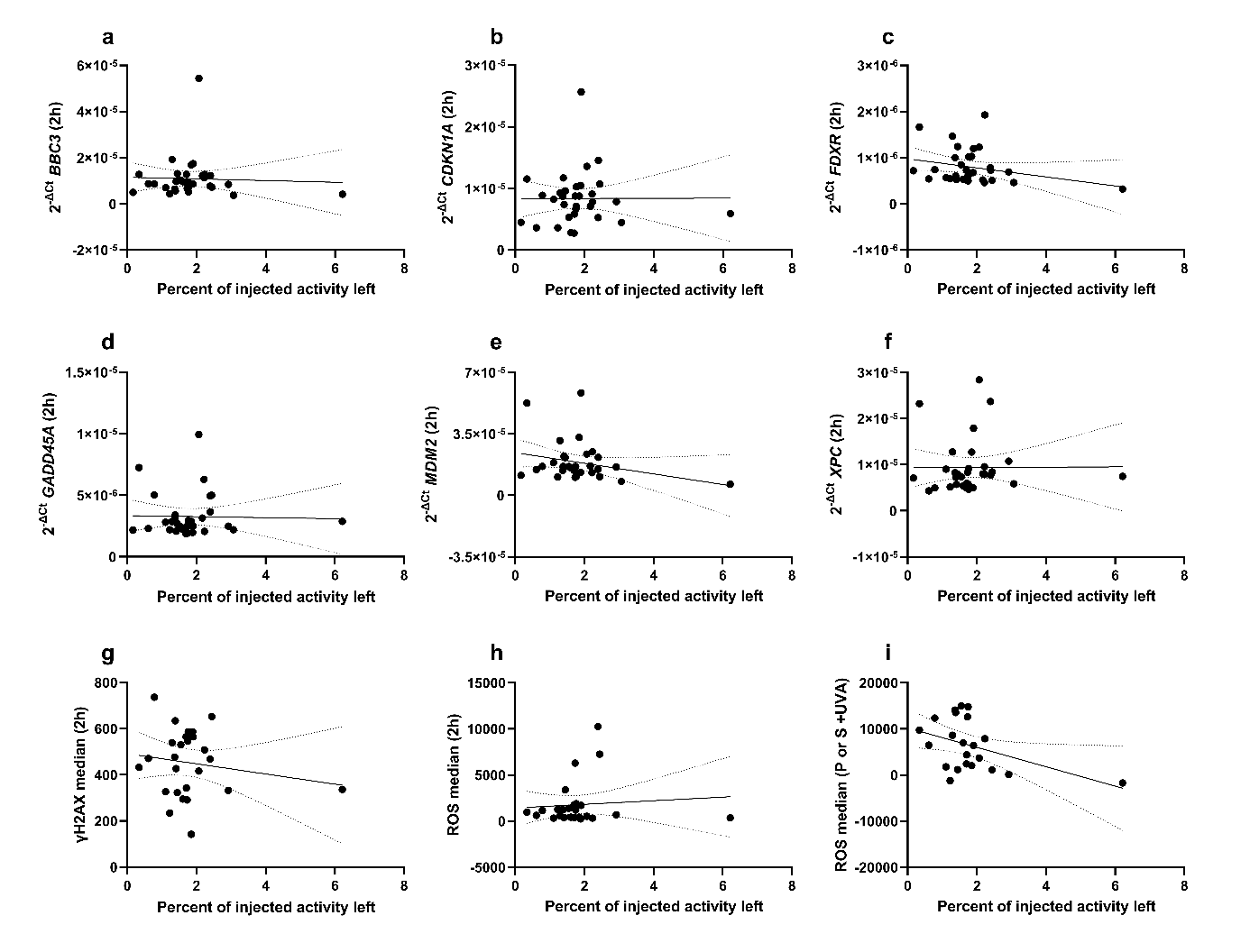
Supplemental Fig. 21 Correlation of percent of injected activity left with raw results from each endpoint at 2 h considering the pool of all patients.** A-F: 2^-ΔCt^ values for *BBC3* (A), *CDKN1A* (B), *FDXR* (C), *GADD45A* (D), *MDM2* (E), and *XPC* (F). G: γH2AX median. H-I: ROS median in blood samples 2 h after PET (P) or scintigraphy (S) procedure (panel H) or after PET (P) or scintigraphy (S) procedure and additional UVA exposure (I). Each symbol represents one individual. Linear regressions (Supplemental Table 22) are represented with a black solid bar and 95% confidence interval are represented with dotted black bands

**
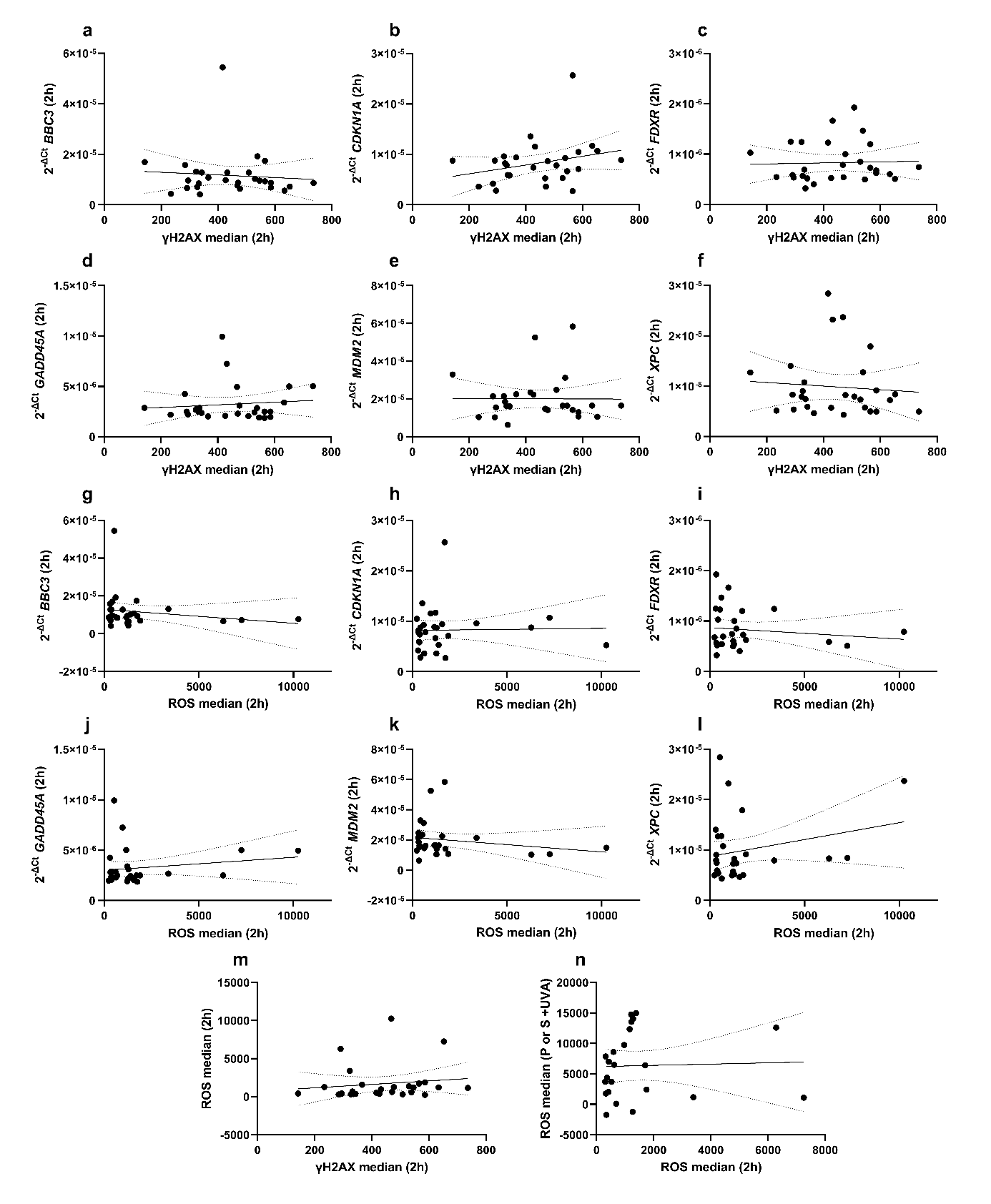
Supplemental Fig. 22 Correlations between endpoints for raw data at 2 h.** Gene expression 2^-ΔCt^ correlation with γH2AX median (A-F) or with ROS median (G-L) and ROS median correlation with γH2AX median (M) or with ROS median after additional UVA exposure (N). Each symbol represents one individual. Linear regressions (Supplemental Table 23) are represented with a black solid bar and 95% confidence interval are represented with dotted black bands

**Supplemental Table 1 Statistical analyses regarding Figure 2**

|  |  |  |  |  |  |  |
| --- | --- | --- | --- | --- | --- | --- |
|  | **Comparison** | | | **Method** | **P-value** | **d** |
| **2B** | *BBC3* (S) | *vs.* | *BBC3* (P) | Unpaired t test | 0.686 | 0.141 |
|  | *CDKN1A* (S) | *vs.* | *CDKN1A* (P) | Unpaired t test | 0.212 | 0.438 |
|  | *FDXR* (S) | *vs.* | *FDXR* (P) | Unpaired t test | 0.139 | **0.519** |
|  | *GADD45A* (S) | *vs.* | *GADD45A* (P) | Unpaired t test | 0.929 | 0.031 |
|  | *MDM2* (S) | *vs.* | *MDM2* (P) | Unpaired t test | 0.259 | 0.393 |
|  | *XPC* (S) | *vs.* | *XPC* (P) | Unpaired t test | 0.768 | 0.103 |
|  | BBC3 (P) | *vs.* | Fold change (0 h) | One-way ANOVA (Dunnett) | 0.410 | **0.741** |
|  | CDKN1A (P) | *vs.* | Fold change (0 h) | One-way ANOVA (Dunnett) | 0.901 | 0.482 |
|  | FDXR (P) | *vs.* | Fold change (0 h) | One-way ANOVA (Dunnett) | 0.781 | **0.553** |
|  | GADD45A (P) | *vs.* | Fold change (0 h) | One-way ANOVA (Dunnett) | 0.999 | 0.250 |
|  | MDM2 (P) | *vs.* | Fold change (0 h) | One-way ANOVA (Dunnett) | 0.898 | 0.435 |
|  | XPC (P) | *vs.* | Fold change (0 h) | One-way ANOVA (Dunnett) | 0.866 | **0.695** |
|  | BBC3 (S) | *vs.* | Fold change (0 h) | One-way ANOVA (Dunnett) | 0.751 | **0.626** |
|  | CDKN1A (S) | *vs.* | Fold change (0 h) | One-way ANOVA (Dunnett) | 1.000 | 0.073 |
|  | FDXR (S) | *vs.* | Fold change (0 h) | One-way ANOVA (Dunnett) | >0.9999 | 0.061 |
|  | GADD45A (S) | *vs.* | Fold change (0 h) | One-way ANOVA (Dunnett) | 0.999 | 0.184 |
|  | MDM2 (S) | *vs.* | Fold change (0 h) | One-way ANOVA (Dunnett) | >0.9999 | 0.016 |
|  | XPC (S) | *vs.* | Fold change (0 h) | One-way ANOVA (Dunnett) | 0.975 | 0.407 |
| **2C** | Scintigraphy | *vs.* | PET | Unpaired t test | 0.368 | **0.648** |
|  | All genes pooled (2 h, P) | *vs.* | All genes pooled (0 h) | One-way ANOVA (Dunnett) | **0.001** | **1.921** |
|  | All genes pooled (2 h, S) | *vs.* | All genes pooled (0 h) | One-way ANOVA (Dunnett) | 0.306 | 0.429 |
| **2D** | Scintigraphy | *vs.* | PET | Unpaired t test | 0.312 | 0.402 |
|  | PET | *vs.* | Fold change (0 h) | One-way ANOVA (Dunnett) | 0.327 | **0.708** |
|  | Scintigraphy | *vs.* | Fold change (0 h) | One-way ANOVA (Dunnett) | **0.014** | **1.002** |
| **2E** | Scintigraphy | *vs.* | PET | Unpaired t test | **0.028** | **0.822** |
|  | Scintigraphy (UVA) | *vs.* | PET (UVA) | Unpaired t test | 0.595 | 0.240 |
|  | PET (UVA) | *vs.* | PET | Paired t test | **0.019** | **1.066** |
|  | Scintigraphy (UVA) | *vs.* | Scintigraphy | Paired t test | 0.954 | 0.036 |
|  | PET | *vs.* | Fold change (0 h) | One-way ANOVA (Dunnett) | 0.231 | **0.768** |
|  | Scintigraphy | *vs.* | Fold change (0 h) | One-way ANOVA (Dunnett) | 0.999 | 0.334 |
|  | PET (UVA) | *vs.* | Fold change (0 h) | One-way ANOVA (Dunnett) | 0.823 | **0.945** |
|  | Scintigraphy (UVA) | *vs.* | Fold change (0 h) | One-way ANOVA (Dunnett) | >0.9999 | 0.014 |
| **2F** | BBC3 (2 h) | *vs.* | Fold change (0 h) | One-way ANOVA (Dunnett) | 0.924 | **0.696** |
|  | CDKN1A (2 h) | *vs.* | Fold change (0 h) | One-way ANOVA (Dunnett) | 1.000 | 0.250 |
|  | FDXR (2 h) | *vs.* | Fold change (0 h) | One-way ANOVA (Dunnett) | 0.999 | 0.320 |
|  | GADD45A (2 h) | *vs.* | Fold change (0 h) | One-way ANOVA (Dunnett) | 1.000 | 0.217 |
|  | MDM2 (2 h) | *vs.* | Fold change (0 h) | One-way ANOVA (Dunnett) | 1.000 | 0.286 |
|  | XPC (2 h) | *vs.* | Fold change (0 h) | One-way ANOVA (Dunnett) | 0.993 | **0.536** |
|  | Pool of genes (2 h) | *vs.* | Pool of genes (0 h) | One-way ANOVA (Dunnett) | 0.999 | 0.470 |
|  | γH2AX (2 h) | *vs.* | Fold change (0 h) | One-way ANOVA (Dunnett) | 0.999 | **0.887** |
|  | ROS (2 h) | *vs.* | Fold change (0 h) | One-way ANOVA (Dunnett) | 0.218 | 0.397 |
|  | ROS (UVA) | *vs.* | Fold change (0 h) | One-way ANOVA (Dunnett) | 0.805 | 0.160 |
| **2G** | Scintigraphy | *vs.* | PET | Unpaired t test | 0.059 | 0.460 |
|  | PET (2 h) | *vs.* | Fold change PET (0 h) | One-way ANOVA (Šídák) | **0.004** | **0.581** |
|  | Scintigraphy (2 h) | *vs.* | Fold change Scintigraphy (0 h) | One-way ANOVA (Šídák) | 0.916 | 0.365 |
|  | P+S (2 h) | *vs.* | Fold change P+S (0 h) | One-way ANOVA (Šídák) | **0.021** | 0.455 |
|  |  |  |  |  |  |  |

**Supplemental Table 2 Linear regression results of correlation analyses regarding Figure 3: correlation of injected activity (MBq) with fold change results from each endpoint considering the pool of all patients**

|  |  |  |  |  |  |  |
| --- | --- | --- | --- | --- | --- | --- |
|  | **Equation** | **95% Confidence Intervals** | | | **Goodness of Fit** | **Pearson** |
|  |  | **Slope** | **Y-intercept** | **X-intercept** | **R squared** | **r** |
| ***BBC3*** | Y = -0.0001763*X + 1.340 | -0.001021 to 0.0006685 | 0.8775 to 1.803 | 1706 to +infinity | 0.0058 | -0.0762 |
| ***CDKN1A*** | Y = -0.0004381*X + 1.302 | -0.001175 to 0.0002991 | 0.8984 to 1.706 | 1402 to +infinity | 0.0452 | -0.2127 |
| ***FDXR*** | Y = -0.0005138*X + 1.350 | -0.001206 to 0.0001783 | 0.9710 to 1.730 | 1388 to +infinity | 0.0689 | -0.2624 |
| ***GADD45A*** | Y = -6.664e-005*X + 1.107 | -0.001001 to 0.0008674 | 0.5951 to 1.619 | 1547 to +infinity | 0.0007 | -0.0261 |
| ***MDM2*** | Y = -0.0003027*X + 1.251 | -0.001038 to 0.0004327 | 0.8479 to 1.654 | 1539 to +infinity | 0.0222 | -0.1491 |
| ***XPC*** | Y = -0.0001342*X + 1.221 | -0.0008393 to 0.0005710 | 0.8350 to 1.608 | 1856 to +infinity | 0.0048 | -0.0695 |
| **γH2AX** | Y = 0.0002275*X + 1.011 | -0.0001414 to 0.0005963 | 0.7967 to 1.226 | -infinity to -1355 | 0.0606 | 0.2462 |
| **ROS (P or S)** | Y = -0.004692*X + 4.092 | -0.008085 to -0.001299 | 2.118 to 6.067 | 683.6 to 1789 | 0.2450 | -0.4950 |
| **ROS (P or S +UVA)** | Y = 0.001312*X - 0.1488 | -0.005179 to 0.007804 | -3.879 to 3.581 | -infinity to +infinity | 0.0088 | 0.0939 |
| **Pool of genes** | Y = -0.0002719*X + 1.262 | -0.0009036 to 0.0003598 | 0.9159 to 1.608 | 1729 to +infinity | 0.0243 | -0.1558 |
| **Pool of endpoints** | Y = -0.0005985*X + 1.475 | -0.001195 to -1.921e-006 | 1.148 to 1.802 | 1469 to 613740 | 0.1190 | -0.3449 |
|  |  |  |  |  |  |  |

**Supplemental Table 3 Linear regression results of correlation analyses regarding Figure 4: correlation of effective (E) dose (mSv) with fold change results from each endpoint considering the pool of all patients**

|  |  |  |  |  |  |  |
| --- | --- | --- | --- | --- | --- | --- |
|  | **Equation** | **95% Confidence Intervals** | | | **Goodness of Fit** | **Pearson** |
|  |  | **Slope** | **Y-intercept** | **X-intercept** | **R squared** | **r** |
| ***BBC3*** | Y = 0.06436*X + 0.8702 | -0.06487 to 0.1936 | 0.07824 to 1.662 | -infinity to -0.4119 | 0.0322 | 0.1795 |
| ***CDKN1A*** | Y = 0.08248*X + 0.5944 | -0.03052 to 0.1955 | -0.09820 to 1.287 | -infinity to 0.5120 | 0.0667 | 0.2583 |
| ***FDXR*** | Y = 0.1051*X + 0.4701 | 0.0007983 to 0.2094 | -0.1693 to 1.109 | -1366 to 0.8223 | 0.1199 | 0.3463 |
| ***GADD45A*** | Y = 0.06083*X + 0.7123 | -0.08232 to 0.2040 | -0.1650 to 1.590 | -infinity to 0.8291 | 0.0237 | 0.1538 |
| ***MDM2*** | Y = 0.1180*X + 0.3989 | 0.01112 to 0.2249 | -0.2562 to 1.054 | -93.11 to 1.159 | 0.1406 | 0.3749 |
| ***XPC*** | Y = 0.05937*X + 0.8018 | -0.04804 to 0.1668 | 0.1435 to 1.460 | -infinity to -0.8753 | 0.0394 | 0.1984 |
| **γH2AX** | Y = -0.01385*X + 1.217 | -0.06749 to 0.03979 | 0.8856 to 1.549 | 22.73 to +infinity | 0.0112 | -0.1057 |
| **ROS (P or S)** | Y = 0.3238*X - 0.3786 | -0.2134 to 0.8610 | -3.699 to 2.941 | -infinity to 4.600 | 0.0581 | 0.2410 |
| **ROS (P or S +UVA)** | Y = -0.1155*X + 1.234 | -1.063 to 0.8320 | -4.599 to 7.068 | -infinity to +infinity | 0.0032 | -0.0568 |
| **Pool of genes** | Y = 0.08169*X + 0.6413 | -0.01283 to 0.1762 | 0.06196 to 1.221 | -infinity to -0.3571 | 0.0911 | 0.3018 |
| **Pool of endpoints** | Y = 0.1037*X + 0.5609 | 0.01284 to 0.1947 | 0.003747 to 1.118 | -85.88 to -0.01952 | 0.1488 | 0.3857 |
|  |  |  |  |  |  |  |

**Supplemental Table 4 Linear regression results of correlation analyses regarding Figure 5: correlation of blood activity left (mBq) with fold change results from each endpoint considering the pool of all patients**

|  |  |  |  |  |  |  |
| --- | --- | --- | --- | --- | --- | --- |
|  | **Equation** | **95% Confidence Intervals** | | | **Goodness of Fit** | **Pearson** |
|  |  | **Slope** | **Y-intercept** | **X-intercept** | **R squared** | **r** |
| ***BBC3*** | Y = -0.003899*X + 1.293 | -0.05112 to 0.04332 | 0.8508 to 1.735 | 32.66 to +infinity | 0.0009 | -0.0308 |
| ***CDKN1A*** | Y = -0.02791*X + 1.331 | -0.06829 to 0.01247 | 0.9524 to 1.709 | 24.08 to +infinity | 0.0623 | -0.2496 |
| ***FDXR*** | Y = -0.01967*X + 1.275 | -0.05828 to 0.01894 | 0.9136 to 1.637 | 27.09 to +infinity | 0.0348 | -0.1866 |
| ***GADD45A*** | Y = 0.004954*X + 1.040 | -0.04716 to 0.05707 | 0.5520 to 1.528 | -infinity to -10.17 | 0.0013 | 0.0354 |
| ***MDM2*** | Y = -0.03459*X + 1.403 | -0.07350 to 0.004323 | 1.039 to 1.768 | 23.19 to +infinity | 0.0990 | -0.3146 |
| ***XPC*** | Y = 0.002552*X + 1.152 | -0.03584 to 0.04095 | 0.7926 to 1.512 | -infinity to -20.02 | 0.0006 | 0.0248 |
| **γH2AX** | Y = -0.0006707*X + 1.134 | -0.02086 to 0.01952 | 0.9360 to 1.332 | 62.79 to +infinity | 0.0002 | -0.0140 |
| **ROS (P or S)** | Y = -0.1534*X + 2.943 | -0.3524 to 0.04555 | 0.9897 to 4.897 | 12.20 to +infinity | 0.0955 | -0.3089 |
| **ROS (P or S +UVA)** | Y = 0.01306*X + 0.4326 | -0.3172 to 0.3433 | -2.781 to 3.647 | -infinity to +infinity | 0.0003 | 0.0185 |
| **Pool of genes** | Y = -0.01309*X + 1.249 | -0.04794 to 0.02175 | 0.9227 to 1.576 | 31.84 to +infinity | 0.0193 | -0.1387 |
| **Pool of endpoints** | Y = -0.02452*X + 1.394 | -0.05841 to 0.009368 | 1.076 to 1.711 | 28.44 to +infinity | 0.0679 | -0.2605 |
|  |  |  |  |  |  |  |

**Supplemental Table 5 Linear regression results of correlation analyses regarding Supplemental Figure 5: correlation of percent of activity left with fold change results from each endpoint considering the pool of all patients**

|  |  |  |  |  |  |  |
| --- | --- | --- | --- | --- | --- | --- |
|  | **Equation** | **95% Confidence Intervals** | | | **Goodness of Fit** | **Pearson** |
|  |  | **Slope** | **Y-intercept** | **X-intercept** | **R squared** | **r** |
| ***BBC3*** | Y = -0.02779*X + 1.311 | -0.2257 to 0.1701 | 0.8973 to 1.726 | 7.332 to +infinity | 0.0027 | -0.0523 |
| ***CDKN1A*** | Y = -0.02272*X + 1.139 | -0.1974 to 0.1520 | 0.7739 to 1.505 | 7.307 to +infinity | 0.0023 | -0.0484 |
| ***FDXR*** | Y = -0.04554*X + 1.195 | -0.2095 to 0.1184 | 0.8514 to 1.538 | 7.056 to +infinity | 0.0106 | -0.1030 |
| ***GADD45A*** | Y = -0.01608*X + 1.111 | -0.2347 to 0.2026 | 0.6533 to 1.568 | 6.330 to +infinity | 0.0008 | -0.0274 |
| ***MDM2*** | Y = -0.1202*X + 1.335 | -0.2863 to 0.04579 | 0.9872 to 1.682 | 5.645 to +infinity | 0.0680 | -0.2607 |
| ***XPC*** | Y = -0.01307*X + 1.197 | -0.1741 to 0.1480 | 0.8605 to 1.534 | 8.498 to +infinity | 0.0009 | -0.0303 |
| **γH2AX** | Y = -0.03859*X + 1.198 | -0.1189 to 0.04173 | 1.031 to 1.364 | 11.25 to +infinity | 0.0394 | -0.1984 |
| **ROS (P or S)** | Y = 0.3614*X + 0.9343 | -0.4739 to 1.197 | -0.8002 to 2.669 | -infinity to 0.7793 | 0.0322 | 0.1793 |
| **ROS (P or S +UVA)** | Y = -0.3274*X + 1.130 | -1.618 to 0.9630 | -1.589 to 3.850 | -infinity to +infinity | 0.0138 | -0.1175 |
| **Pool of genes** | Y = -0.04091*X + 1.215 | -0.1877 to 0.1059 | 0.9075 to 1.522 | 7.834 to +infinity | 0.0107 | -0.1033 |
| **Pool of endpoints** | Y = 0.004899*X + 1.180 | -0.1423 to 0.1521 | 0.8721 to 1.488 | -infinity to -5.924 | 0.0002 | 0.0124 |
|  |  |  |  |  |  |  |

**Supplemental Table 6 Linear regression results of correlation analyses regarding Supplemental Figure 6: correlations between endpoints for fold change values**

|  |  |  |  |  |  |  |  |
| --- | --- | --- | --- | --- | --- | --- | --- |
|  |  | **Equation** | **95% Confidence Intervals** | | | **Goodness of Fit** | **Pearson** |
|  |  |  | **Slope** | **Y-intercept** | **X-intercept** | **R squared** | **r** |
| **Fold γH2AX:GE/ROS** | ***BBC3*** | Y = 0.6666*X + 0.4776 | -0.2711 to 1.604 | -0.5963 to 1.551 | -infinity to 0.3767 | 0.0759 | 0.2755 |
|  | ***CDKN1A*** | Y = 0.3470*X + 0.6545 | -0.5456 to 1.240 | -0.3677 to 1.677 | -infinity to 0.3011 | 0.0240 | 0.1548 |
|  | ***FDXR*** | Y = 0.2997*X + 0.6871 | -0.3634 to 0.9628 | -0.07226 to 1.447 | -infinity to 0.07591 | 0.0321 | 0.1793 |
|  | ***GADD45A*** | Y = 1.116*X - 0.1550 | -0.1017 to 2.334 | -1.550 to 1.240 | -infinity to 0.6769 | 0.1201 | 0.3466 |
|  | ***MDM2*** | Y = 0.7250*X + 0.2634 | -0.1202 to 1.570 | -0.7046 to 1.231 | -infinity to 0.4549 | 0.1068 | 0.3268 |
|  | ***XPC*** | Y = 0.4694*X + 0.6466 | -0.4832 to 1.422 | -0.4443 to 1.738 | -infinity to 0.3170 | 0.0380 | 0.1948 |
|  | **ROS (P or S)** | Y = -3.320*X + 5.324 | -7.175 to 0.5353 | 0.9083 to 9.739 | 1.298 to +infinity | 0.1076 | -0.3279 |
|  |  |  |  |  |  |  |  |
| **FOLD ROS:GE/ROS (P or S +UVA)** | ***BBC3*** | Y = -0.01446*X + 1.252 | -0.1106 to 0.08171 | 1.005 to 1.499 | 12.53 to +infinity | 0.0037 | -0.0605 |
|  | ***CDKN1A*** | Y = 0.04518*X + 0.9744 | -0.04219 to 0.1325 | 0.7498 to 1.199 | -infinity to -6.137 | 0.0416 | 0.2041 |
|  | ***FDXR*** | Y = -0.0001170*X + 1.025 | -0.06670 to 0.06646 | 0.8541 to 1.196 | 16.82 to +infinity | 0.0000 | -0.0007 |
|  | ***GADD45A*** | Y = -0.02008*X + 1.135 | -0.1481 to 0.1079 | 0.8064 to 1.465 | 8.792 to +infinity | 0.0040 | -0.0631 |
|  | ***MDM2*** | Y = -0.02143*X + 1.115 | -0.1093 to 0.06649 | 0.8886 to 1.341 | 11.30 to +infinity | 0.0096 | -0.0978 |
|  | ***XPC*** | Y = 0.01189*X + 1.157 | -0.08392 to 0.1077 | 0.9108 to 1.403 | -infinity to -9.169 | 0.0025 | 0.0500 |
|  | **ROS (P or S +UVA)** | Y = -0.07120*X + 0.7688 | -0.7131 to 0.5707 | -1.039 to 2.576 | -infinity to +infinity | 0.0025 | -0.0503 |
|  |  |  |  |  |  |  |  |

**Supplemental Table 7 Linear regression results of correlation analyses regarding Supplemental Figure 7: correlation of patients age (years) with fold change results from each endpoint considering the pool of all patients**

|  |  |  |  |  |  |  |
| --- | --- | --- | --- | --- | --- | --- |
|  | **Equation** | **95% Confidence Intervals** | | | **Goodness of Fit** | **Pearson** |
|  |  | **Slope** | **Y-intercept** | **X-intercept** | **R squared** | **r** |
| ***BBC3*** | Y = 0.003223*X + 1.052 | -0.01450 to 0.02095 | -0.1328 to 2.237 | -infinity to 6.420 | 0.0043 | 0.0653 |
| ***CDKN1A*** | Y = -0.007858*X + 1.602 | -0.02329 to 0.007577 | 0.5702 to 2.634 | 111.7 to +infinity | 0.0325 | -0.1803 |
| ***FDXR*** | Y = -0.009055*X + 1.701 | -0.02370 to 0.005589 | 0.7220 to 2.680 | 111.8 to +infinity | 0.0472 | -0.2173 |
| ***GADD45A*** | Y = 0.01395*X + 0.1723 | -0.005159 to 0.03305 | -1.105 to 1.449 | -infinity to 33.94 | 0.0646 | 0.2542 |
| ***MDM2*** | Y = -0.005781*X + 1.477 | -0.02114 to 0.009577 | 0.4500 to 2.503 | 117.0 to +infinity | 0.0180 | -0.1343 |
| ***XPC*** | Y = 0.009668*X + 0.5359 | -0.005073 to 0.02441 | -0.4494 to 1.521 | -infinity to 18.61 | 0.0528 | 0.2298 |
| **γH2AX** | Y = 0.004332*X + 0.8378 | -0.003277 to 0.01194 | 0.3228 to 1.353 | -infinity to -27.18 | 0.0500 | 0.2237 |
| **ROS (P or S)** | Y = -0.03630*X + 4.008 | -0.1140 to 0.04136 | -1.249 to 9.265 | 77.98 to +infinity | 0.0343 | -0.1852 |
| **ROS (P or S +UVA)** | Y = -0.003528*X + 0.8761 | -0.1275 to 0.1205 | -7.470 to 9.223 | -infinity to +infinity | 0.0002 | -0.0129 |
| **Pool of genes** | Y = 0.0006908*X + 1.090 | -0.01271 to 0.01409 | 0.1944 to 1.985 | -infinity to -13.94 | 0.0003 | 0.0186 |
| **Pool of endpoints** | Y = -0.003010*X + 1.384 | -0.01627 to 0.01025 | 0.4979 to 2.271 | 138.2 to +infinity | 0.0066 | -0.0815 |
|  |  |  |  |  |  |  |

**Supplemental Table 8 Linear regression results of correlation analyses regarding Supplemental Figure 8: correlation of patients body mass (Kg) with fold change results from each endpoint considering the pool of all patients**

|  |  |  |  |  |  |  |
| --- | --- | --- | --- | --- | --- | --- |
|  | **Equation** | **95% Confidence Intervals** | | | **Goodness of Fit** | **Pearson** |
|  |  | **Slope** | **Y-intercept** | **X-intercept** | **R squared** | **r** |
| ***BBC3*** | Y = -0.006618*X + 1.845 | -0.02011 to 0.006869 | 0.6674 to 3.022 | 148.4 to +infinity | 0.0313 | -0.1769 |
| ***CDKN1A*** | Y = -0.002428*X + 1.294 | -0.01457 to 0.009718 | 0.2340 to 2.354 | 159.4 to +infinity | 0.0053 | -0.0730 |
| ***FDXR*** | Y = -0.0003112*X + 1.143 | -0.01179 to 0.01117 | 0.1415 to 2.145 | 179.7 to +infinity | 0.0001 | -0.0099 |
| ***GADD45A*** | Y = -0.008166*X + 1.805 | -0.02314 to 0.006805 | 0.4985 to 3.112 | 132.3 to +infinity | 0.0384 | -0.1959 |
| ***MDM2*** | Y = 0.008130*X + 0.4029 | -0.003472 to 0.01973 | -0.6096 to 1.415 | -infinity to 31.28 | 0.0618 | 0.2486 |
| ***XPC*** | Y = -0.006472*X + 1.737 | -0.01795 to 0.005008 | 0.7351 to 2.739 | 150.8 to +infinity | 0.0409 | -0.2022 |
| **γH2AX** | Y = -0.0003447*X + 1.157 | -0.006222 to 0.005532 | 0.6319 to 1.682 | 268.6 to +infinity | 0.0006 | -0.0242 |
| **ROS (P or S)** | Y = -0.04362*X + 5.508 | -0.09895 to 0.01171 | 0.5674 to 10.45 | 101.5 to +infinity | 0.0954 | -0.3088 |
| **ROS (P or S +UVA)** | Y = 0.02116*X - 1.169 | -0.08251 to 0.1248 | -10.41 to 8.076 | -infinity to +infinity | 0.0090 | 0.0948 |
| **Pool of genes** | Y = -0.002644*X + 1.371 | -0.01293 to 0.007643 | 0.4733 to 2.269 | 173.6 to +infinity | 0.0088 | -0.0937 |
| **Pool of endpoints** | Y = -0.005912*X + 1.709 | -0.01578 to 0.003959 | 0.8474 to 2.570 | 161.3 to +infinity | 0.0459 | -0.2143 |
|  |  |  |  |  |  |  |

**Supplemental Table 9 Linear regression results of correlation analyses regarding Supplemental Figure 9: correlation of patients sex with fold change results from each endpoint considering the pool of all patients**

|  |  |  |  |  |  |  |
| --- | --- | --- | --- | --- | --- | --- |
|  | **Equation** | **95% Confidence Intervals** | | | **Goodness of Fit** | **Pearson** |
|  |  | **Slope** | **Y-intercept** | **X-intercept** | **R squared** | **r** |
| ***BBC3*** | Y = -0.3262*X + 1.313 | -0.8528 to 0.2004 | 1.111 to 1.514 | 1.604 to +infinity | 0.0474 | -0.2177 |
| ***CDKN1A*** | Y = -0.2994*X + 1.128 | -0.7637 to 0.1649 | 0.9495 to 1.306 | 1.539 to +infinity | 0.0512 | -0.2262 |
| ***FDXR*** | Y = -0.1277*X + 1.122 | -0.5811 to 0.3256 | 0.9483 to 1.296 | 2.020 to +infinity | 0.0102 | -0.1009 |
| ***GADD45A*** | Y = -0.1826*X + 1.119 | -0.7791 to 0.4139 | 0.8906 to 1.348 | 1.515 to +infinity | 0.0120 | -0.1096 |
| ***MDM2*** | Y = -0.3212*X + 1.142 | -0.7776 to 0.1352 | 0.9675 to 1.317 | 1.529 to +infinity | 0.0604 | -0.2457 |
| ***XPC*** | Y = -0.3422*X + 1.224 | -0.7855 to 0.1011 | 1.054 to 1.394 | 1.618 to +infinity | 0.0717 | -0.2678 |
| **γH2AX** | Y = 0.002637*X + 1.127 | -0.3110 to 0.3162 | 1.044 to 1.211 | -infinity to -3.485 | 0.0000 | 0.0034 |
| **ROS (P or S)** | Y = -1.078*X + 1.657 | -4.223 to 2.067 | 0.8165 to 2.497 | 0.3752 to +infinity | 0.0187 | -0.1369 |
| **ROS (P or S +UVA)** | Y = 0.6931*X + 0.6119 | -6.172 to 7.559 | -0.8197 to 2.043 | -infinity to +infinity | 0.0021 | 0.0458 |
| **Pool of genes** | Y = -0.2666*X + 1.175 | -0.6621 to 0.1290 | 1.023 to 1.326 | 1.840 to +infinity | 0.0556 | -0.2358 |
| **Pool of endpoints** | Y = -0.3214*X + 1.233 | -0.7086 to 0.06586 | 1.084 to 1.381 | 1.800 to +infinity | 0.0820 | -0.2863 |
|  |  |  |  |  |  |  |

**Supplemental Table 10 Linear regression results of correlation analyses regarding Supplemental Figure 10: correlation of previous radiotherapy (RT) treatment record with fold change results from each endpoint considering the pool of all patients**

|  |  |  |  |  |  |  |
| --- | --- | --- | --- | --- | --- | --- |
|  | **Equation** | **95% Confidence Intervals** | | | **Goodness of Fit** | **Pearson** |
|  |  | **Slope** | **Y-intercept** | **X-intercept** | **R squared** | **r** |
| ***BBC3*** | Y = 0.1533*X + 1.228 | -0.2938 to 0.6004 | 1.012 to 1.445 | -infinity to -1.839 | 0.0150 | 0.1225 |
| ***CDKN1A*** | Y = 0.1679*X + 1.044 | -0.2255 to 0.5612 | 0.8532 to 1.235 | -infinity to -1.663 | 0.0231 | 0.1519 |
| ***FDXR*** | Y = 0.3335*X + 1.025 | -0.02752 to 0.6946 | 0.8498 to 1.200 | -infinity to -1.324 | 0.0996 | 0.3156 |
| ***GADD45A*** | Y = -0.1559*X + 1.129 | -0.6537 to 0.3420 | 0.8876 to 1.371 | 1.865 to +infinity | 0.0126 | -0.1120 |
| ***MDM2*** | Y = 0.1472*X + 1.061 | -0.2423 to 0.5367 | 0.8717 to 1.250 | -infinity to -1.773 | 0.0182 | 0.1349 |
| ***XPC*** | Y = -0.1171*X + 1.201 | -0.4989 to 0.2648 | 1.016 to 1.387 | 2.559 to +infinity | 0.0120 | -0.1097 |
| **γH2AX** | Y = 0.1132*X + 1.111 | -0.1131 to 0.3394 | 1.026 to 1.197 | -infinity to -3.167 | 0.0391 | 0.1976 |
| **ROS (P or S)** | Y = -0.8795*X + 1.706 | -3.189 to 1.430 | 0.8327 to 2.579 | 0.5432 to +infinity | 0.0230 | -0.1517 |
| **ROS (P or S +UVA)** | Y = -2.289*X + 1.040 | -5.838 to 1.259 | -0.4398 to 2.520 | -infinity to +infinity | 0.0790 | -0.2810 |
| **Pool of genes** | Y = 0.08817*X + 1.115 | -0.2502 to 0.4265 | 0.9506 to 1.279 | -infinity to -2.400 | 0.0087 | 0.0934 |
| **Pool of endpoints** | Y = 0.03576*X + 1.177 | -0.3014 to 0.3729 | 1.014 to 1.341 | -infinity to -2.918 | 0.0015 | 0.0382 |
|  |  |  |  |  |  |  |

**Supplemental Table 11 Linear regression results of correlation analyses regarding Supplemental Figure 11: correlation of previous chemotherapy (CHT) treatment record with fold change results from each endpoint considering the pool of all patients**

|  |  |  |  |  |  |  |
| --- | --- | --- | --- | --- | --- | --- |
|  | **Equation** | **95% Confidence Intervals** | | | **Goodness of Fit** | **Pearson** |
|  |  | **Slope** | **Y-intercept** | **X-intercept** | **R squared** | **r** |
| ***BBC3*** | Y = 0.04328*X + 1.256 | -0.4291 to 0.5156 | 1.041 to 1.470 | -infinity to -2.216 | 0.0011 | 0.0330 |
| ***CDKN1A*** | Y = 0.1840*X + 1.046 | -0.2282 to 0.5962 | 0.8586 to 1.233 | -infinity to -1.580 | 0.0252 | 0.1587 |
| ***FDXR*** | Y = 0.2299*X + 1.056 | -0.1606 to 0.6204 | 0.8789 to 1.233 | -infinity to -1.544 | 0.0430 | 0.2074 |
| ***GADD45A*** | Y = -0.1717*X + 1.128 | -0.6936 to 0.3503 | 0.8910 to 1.365 | 1.741 to +infinity | 0.0138 | -0.1176 |
| ***MDM2*** | Y = 0.1474*X + 1.065 | -0.2616 to 0.5563 | 0.8794 to 1.250 | -infinity to -1.732 | 0.0166 | 0.1287 |
| ***XPC*** | Y = -0.1964*X + 1.214 | -0.5931 to 0.2004 | 1.034 to 1.394 | 2.157 to +infinity | 0.0308 | -0.1754 |
| **γH2AX** | Y = -0.1509*X + 1.149 | -0.3735 to 0.07180 | 1.065 to 1.233 | 3.150 to +infinity | 0.0694 | -0.2635 |
| **ROS (P or S)** | Y = 1.101*X + 1.423 | -1.193 to 3.395 | 0.5557 to 2.290 | -infinity to -0.2287 | 0.0361 | 0.1900 |
| **ROS (P or S +UVA)** | Y = 0.4014*X + 0.5722 | -3.292 to 4.095 | -0.9680 to 2.112 | -infinity to +infinity | 0.0024 | 0.0493 |
| **Pool of genes** | Y = 0.03942*X + 1.127 | -0.3168 to 0.3957 | 0.9657 to 1.289 | -infinity to -2.638 | 0.0016 | 0.0398 |
| **Pool of endpoints** | Y = 0.1159*X + 1.162 | -0.2356 to 0.4674 | 1.002 to 1.321 | -infinity to -2.306 | 0.0139 | 0.1179 |
|  |  |  |  |  |  |  |

**Supplemental Table 12 Statistical analyses regarding Figure 6: raw data at 0 and 2 h for PET and scintigraphy patients**

|  | **Comparison** | | | **Method** | **P-value** | **d** |
| --- | --- | --- | --- | --- | --- | --- |
| **6A** | PET *BBC3* (2 h) | vs. | PET *BBC3* (0 h) | One-way ANOVA (Šidák) | 0.977 | 0.468 |
|  | PET *CDKN1A* (2 h) | vs. | PET *CDKN1A* (0 h) | One-way ANOVA (Šidák) | >0.9999 | 0.106 |
|  | PET *FDXR* (2 h) | vs. | PET *FDXR* (0 h) | One-way ANOVA (Šidák) | >0.9999 | 0.289 |
|  | PET *GADD45A* (2 h) | vs. | PET *GADD45A* (0 h) | One-way ANOVA (Šidák) | >0.9999 | 0.123 |
|  | PET *MDM2* (2 h) | vs. | PET *MDM2* (0 h) | One-way ANOVA (Šidák) | 0.811 | 0.191 |
|  | PET *XPC* (2 h) | vs. | PET *XPC* (0 h) | One-way ANOVA (Šidák) | 0.928 | 0.448 |
| **6B** | Scintigraphy BBC3 (2 h) | vs. | Scintigraphy BBC3 (0 h) | One-way ANOVA (Šidák) | 0.396 | 0.369 |
|  | Scintigraphy CDKN1A (2 h) | vs. | Scintigraphy CDKN1A (0 h) | One-way ANOVA (Šidák) | 1.000 | 0.190 |
|  | Scintigraphy FDXR (2 h) | vs. | Scintigraphy FDXR (0 h) | One-way ANOVA (Šidák) | >0.9999 | 0.090 |
|  | Scintigraphy GADD45A (2 h) | vs. | Scintigraphy GADD45A (0 h) | One-way ANOVA (Šidák) | >0.9999 | 0.135 |
|  | Scintigraphy MDM2 (2 h) | vs. | Scintigraphy MDM2 (0 h) | One-way ANOVA (Šidák) | 0.989 | 0.155 |
|  | Scintigraphy XPC (2 h) | vs. | Scintigraphy XPC (0 h) | One-way ANOVA (Šidák) | 0.968 | 0.300 |
| **6C** | *BBC3* (2 h) | vs. | *BBC3* (0 h) | One-way ANOVA (Šidák) | >0.9999 | 0.342 |
|  | *CDKN1A* (2 h) | vs. | *CDKN1A* (0 h) | One-way ANOVA (Šidák) | >0.9999 | 0.010 |
|  | *FDXR* (2 h) | vs. | *FDXR* (0 h) | One-way ANOVA (Šidák) | >0.9999 | 0.063 |
|  | *GADD45A* (2 h) | vs. | *GADD45A* (0 h) | One-way ANOVA (Šidák) | >0.9999 | 0.128 |
|  | *MDM2* (2 h) | vs. | *MDM2* (0 h) | One-way ANOVA (Šidák) | >0.9999 | 0.065 |
|  | *XPC* (2 h) | vs. | *XPC* (0 h) | One-way ANOVA (Šidák) | >0.9999 | 0.370 |
| **6D** | All genes (2 h) | vs. | All genes (0 h) | Paired t test | 0.148 | 0.201 |
| **6E** | PET γH2AX (2 h) | vs. | PET γH2AX (0 h) | Paired t test | 0.108 | 0.250 |
| **6F** | Scintigraphy γH2AX (2 h) | vs. | Scintigraphy γH2AX (0 h) | Paired t test | **0.020** | 0.423 |
| **6G** | γH2AX (2 h) | vs. | γH2AX (0 h) | Paired t test | **0.004** | 0.357 |
| **6H** | ROS (PET, 2 h) | vs. | ROS (0 h) | One-way ANOVA (Šidák) | 1.000 | 0.034 |
|  | ROS (UVA) | vs. | ROS (0 h) | One-way ANOVA (Šidák) | **0.012** | 0.303 |
|  | ROS (PET+UVA) | vs. | ROS (0 h) | One-way ANOVA (Šidák) | **0.004** | 0.462 |
|  | ROS (PET+UVA) | vs. | ROS (PET, 2 h) | One-way ANOVA (Šidák) | **0.003** | **0.562** |
| **6I** | ROS (Scintigraphy,2 h) | vs. | ROS (0 h) | One-way ANOVA (Šidák) | >0.9999 | 0.071 |
|  | ROS (UVA) | vs. | ROS (0 h) | One-way ANOVA (Šidák) | **0.002** | **1.116** |
|  | ROS (Scintigraphy+UVA) | vs. | ROS (0 h) | One-way ANOVA (Šidák) | **0.002** | **1.458** |
|  | ROS (Scintigraphy+UVA) | vs. | ROS (Scintigraphy, 2 h) | One-way ANOVA (Šidák) | **0.001** | **1.773** |
| **6J** | ROS (P/S, 2 h) | vs. | ROS (0 h) | One-way ANOVA (Šidák) | 1.000 | 0.047 |
|  | ROS (UVA) | vs. | ROS (0 h) | One-way ANOVA (Šidák) | **0.012** | **0.748** |
|  | ROS (P/S+UVA) | vs. | ROS (0 h) | One-way ANOVA (Šidák) | **0.004** | **0.926** |
|  | ROS (P/S+UVA) | vs. | ROS (P/S, 2 h) | One-way ANOVA (Šidák) | **0.003** | **1.111** |
| **6K** | PET (2 h) | vs. | PET (0 h) | Paired t test | 0.194 | 0.327 |
| **6L** | Scintigraphy (2 h) | vs. | Scintigraphy (0 h) | Paired t test | 0.427 | 0.184 |
| **6M** | All endpoints (2 h) | vs. | All endpoints (0 h) | Paired t test | 0.124 | 0.262 |

**Supplemental Table 13 Linear regression results of correlation analyses regarding Supplemental Figure 12: correlation of patient´s age (years) with raw results from each endpoint at 0 h considering the pool of all patients**

|  |  |  |  |  |  |  |
| --- | --- | --- | --- | --- | --- | --- |
|  | **Equation** | **95% Confidence Intervals** | | | **Goodness of Fit** | **Pearson** |
|  |  | **Slope** | **Y-intercept** | **X-intercept** | **R squared** | **r** |
| ***BBC3* (0 h)** | Y = 6.320e-009*X + 8.330e-006 | -1.185e-007 to 1.312e-007 | -1.443e-008 to 1.667e-005 | -infinity to 0.1114 | 0.0003 | 0.0182 |
| ***CDKN1A* (0 h)** | Y = -6.095e-008*X + 1.232e-005 | -2.030e-007 to 8.112e-008 | 2.821e-006 to 2.181e-005 | 105.8 to +infinity | 0.0233 | -0.1527 |
| ***FDXR* (0 h)** | Y = -1.986e-009*X + 9.066e-007 | -1.376e-008 to 9.787e-009 | 1.197e-007 to 1.694e-006 | 121.5 to +infinity | 0.0037 | -0.0606 |
| ***GADD45A* (0 h)** | Y = -4.586e-009*X + 3.379e-006 | -3.507e-008 to 2.590e-008 | 1.342e-006 to 5.417e-006 | 153.1 to +infinity | 0.0029 | -0.0541 |
| ***MDM2* (0 h)** | Y = -8.276e-008*X + 2.377e-005 | -3.962e-007 to 2.307e-007 | 2.820e-006 to 4.473e-005 | 111.2 to +infinity | 0.0090 | -0.0946 |
| ***XPC* (0 h)** | Y = -1.183e-008*X + 8.531e-006 | -8.640e-008 to 6.273e-008 | 3.547e-006 to 1.351e-005 | 155.1 to +infinity | 0.0033 | -0.0571 |
| **γH2AX (0 h)** | Y = -1.446*X + 493.1 | -6.008 to 3.117 | 184.3 to 801.9 | 132.2 to +infinity | 0.0161 | -0.1267 |
| **ROS (0 h)** | Y = 17.65*X + 698.8 | -142.2 to 177.5 | -10123 to 11520 | -infinity to 62.43 | 0.0020 | 0.0445 |
|  |  |  |  |  |  |  |

**Supplemental Table 14 Linear regression results of correlation analyses regarding Supplemental Figure 13: correlation of patient´s body mass (Kg) with raw results from each endpoint at 0 h considering the pool of all patients**

|  |  |  |  |  |  |  |
| --- | --- | --- | --- | --- | --- | --- |
|  | **Equation** | **95% Confidence Intervals** | | | **Goodness of Fit** | **Pearson** |
|  |  | **Slope** | **Y-intercept** | **X-intercept** | **R squared** | **r** |
| ***BBC3* (0 h)** | Y = -1.569e-008*X + 9.934e-006 | -1.093e-007 to 7.793e-008 | 1.764e-006 to 1.810e-005 | 163.5 to +infinity | 0.0038 | -0.0613 |
| ***CDKN1A* (0 h)** | Y = -3.844e-008*X + 1.158e-005 | -1.490e-007 to 7.217e-008 | 1.924e-006 to 2.123e-005 | 140.2 to +infinity | 0.0160 | -0.1263 |
| ***FDXR* (0 h)** | Y = 3.422e-010*X + 7.136e-007 | -7.420e-009 to 8.104e-009 | 3.624e-008 to 1.391e-006 | -infinity to -4.528 | 0.0003 | 0.0162 |
| ***GADD45A* (0 h)** | Y = 7.584e-009*X + 2.412e-006 | -1.588e-008 to 3.105e-008 | 3.641e-007 to 4.460e-006 | -infinity to -11.83 | 0.0138 | 0.1176 |
| ***MDM2* (0 h)** | Y = -1.393e-007*X + 3.009e-005 | -3.762e-007 to 9.762e-008 | 9.416e-006 to 5.077e-005 | 132.9 to +infinity | 0.0443 | -0.2105 |
| ***XPC* (0 h)** | Y = -2.077e-008*X + 9.527e-006 | -7.822e-008 to 3.667e-008 | 4.514e-006 to 1.454e-005 | 184.3 to +infinity | 0.0172 | -0.1313 |
| **γH2AX (0 h)** | Y = 0.7259*X + 336.8 | -2.661 to 4.113 | 34.31 to 639.3 | -infinity to -8.424 | 0.0077 | 0.0879 |
| **ROS (0 h)** | Y = 94.27*X - 6173 | -12.96 to 201.5 | -15748 to 3402 | -infinity to 82.83 | 0.1159 | 0.3405 |
|  |  |  |  |  |  |  |

**Supplemental Table 15 Linear regression results of correlation analyses regarding Supplemental Figure 14: correlation of patient´s sex with raw results from each endpoint at 0 h considering the pool of all patients**

|  |  |  |  |  |  |  |
| --- | --- | --- | --- | --- | --- | --- |
|  | **Equation** | **95% Confidence Intervals** | | | **Goodness of Fit** | **Pearson** |
|  |  | **Slope** | **Y-intercept** | **X-intercept** | **R squared** | **r** |
| ***BBC3* (0 h)** | Y = 6.306e-007*X + 8.654e-006 | -3.155e-006 to 4.416e-006 | 7.202e-006 to 1.011e-005 | -infinity to -1.806 | 0.0036 | 0.0599 |
| ***CDKN1A* (0 h)** | Y = 1.986e-006*X + 8.004e-006 | -2.321e-006 to 6.294e-006 | 6.353e-006 to 9.656e-006 | -infinity to -1.138 | 0.0268 | 0.1638 |
| ***FDXR* (0 h)** | Y = 5.263e-008*X + 7.679e-007 | -3.051e-007 to 4.104e-007 | 6.307e-007 to 9.051e-007 | -infinity to -1.714 | 0.0028 | 0.0529 |
| ***GADD45A* (0 h)** | Y = -5.116e-007*X + 3.152e-006 | -1.420e-006 to 3.972e-007 | 2.804e-006 to 3.501e-006 | 2.295 to +infinity | 0.0395 | -0.1987 |
| ***MDM2* (0 h)** | Y = 4.450e-006*X + 1.766e-005 | -4.980e-006 to 1.388e-005 | 1.404e-005 to 2.128e-005 | -infinity to -1.140 | 0.0281 | 0.1675 |
| ***XPC* (0 h)** | Y = -5.251e-007*X + 7.827e-006 | -2.786e-006 to 1.736e-006 | 6.960e-006 to 8.694e-006 | 2.911 to +infinity | 0.0069 | -0.0833 |
| **γH2AX (0 h)** | Y = 7.654*X + 395.8 | -177.1 to 192.4 | 346.5 to 445.2 | -infinity to -1.973 | 0.0003 | 0.0167 |
| **ROS (0 h)** | Y = -333.6*X + 1904 | -6760 to 6093 | 185.9 to 3621 | 0.1959 to +infinity | 0.0004 | -0.0209 |
|  |  |  |  |  |  |  |

**Supplemental Table 16 Linear regression results of correlation analyses regarding Supplemental Figure 15: correlation of previous radiotherapy (RT) treatment record with raw results from each endpoint at 0 h considering the pool of all patients**

|  |  |  |  |  |  |  |
| --- | --- | --- | --- | --- | --- | --- |
|  | **Equation** | **95% Confidence Intervals** | | | **Goodness of Fit** | **Pearson** |
|  |  | **Slope** | **Y-intercept** | **X-intercept** | **R squared** | **r** |
| ***BBC3* (0 h)** | Y = 8.050e-007*X + 8.557e-006 | -2.348e-006 to 3.958e-006 | 7.028e-006 to 1.009e-005 | -infinity to -1.942 | 0.0084 | 0.0915 |
| ***CDKN1A* (0 h)** | Y = 2.254e-006*X + 7.766e-006 | -1.301e-006 to 5.808e-006 | 6.042e-006 to 9.490e-006 | -infinity to -1.154 | 0.0495 | 0.2226 |
| ***FDXR* (0 h)** | Y = 2.112e-010*X + 7.756e-007 | -2.989e-007 to 2.993e-007 | 6.305e-007 to 9.207e-007 | -infinity to -2.321 | 0.0000 | 0.0003 |
| ***GADD45A* (0 h)** | Y = 6.344e-007*X + 2.928e-006 | -1.053e-007 to 1.374e-006 | 2.569e-006 to 3.286e-006 | -infinity to -1.982 | 0.0871 | 0.2951 |
| ***MDM2* (0 h)** | Y = 2.933e-006*X + 1.762e-005 | -4.983e-006 to 1.085e-005 | 1.378e-005 to 2.146e-005 | -infinity to -1.413 | 0.0175 | 0.1323 |
| ***XPC* (0 h)** | Y = 7.098e-008*X + 7.733e-006 | -1.823e-006 to 1.965e-006 | 6.815e-006 to 8.652e-006 | -infinity to -3.687 | 0.0002 | 0.0135 |
| **γH2AX (0 h)** | Y = 66.92*X + 386.8 | -66.36 to 200.2 | 336.5 to 437.2 | -infinity to -1.817 | 0.0394 | 0.1984 |
| **ROS (0 h)** | Y = -873.0*X + 2004 | -5591 to 3845 | 221.2 to 3788 | 0.2749 to +infinity | 0.0055 | -0.0744 |
|  |  |  |  |  |  |  |

**Supplemental Table 17 Linear regression results of correlation analyses regarding Supplemental Figure 16: correlation of previous chemotherapy (CHT) treatment record with raw results from each endpoint at 0 h considering the pool of all patients**

|  |  |  |  |  |  |  |
| --- | --- | --- | --- | --- | --- | --- |
|  | **Equation** | **95% Confidence Intervals** | | | **Goodness of Fit** | **Pearson** |
|  |  | **Slope** | **Y-intercept** | **X-intercept** | **R squared** | **r** |
| ***BBC3* (0 h)** | Y = -5.439e-007*X + 8.859e-006 | -3.860e-006 to 2.772e-006 | 7.354e-006 to 1.036e-005 | 2.441 to +infinity | 0.0035 | -0.0590 |
| ***CDKN1A* (0 h)** | Y = -1.002e-006*X + 8.503e-006 | -4.810e-006 to 2.805e-006 | 6.775e-006 to 1.023e-005 | 1.893 to +infinity | 0.0089 | -0.0944 |
| ***FDXR* (0 h)** | Y = 1.242e-009*X + 7.754e-007 | -3.126e-007 to 3.151e-007 | 6.330e-007 to 9.178e-007 | -infinity to -2.223 | 0.0000 | 0.0014 |
| ***GADD45A* (0 h)** | Y = 2.011e-007*X + 3.035e-006 | -6.079e-007 to 1.010e-006 | 2.668e-006 to 3.403e-006 | -infinity to -2.819 | 0.0079 | 0.0891 |
| ***MDM2* (0 h)** | Y = -3.949e-006*X + 1.913e-005 | -1.221e-005 to 4.308e-006 | 1.538e-005 to 2.287e-005 | 1.669 to +infinity | 0.0288 | -0.1697 |
| ***XPC* (0 h)** | Y = -1.271e-006*X + 8.012e-006 | -3.204e-006 to 6.632e-007 | 7.134e-006 to 8.889e-006 | 2.604 to +infinity | 0.0530 | -0.2302 |
| **γH2AX (0 h)** | Y = 57.88*X + 388.1 | -76.09 to 191.8 | 337.5 to 438.8 | -infinity to -1.903 | 0.0294 | 0.1716 |
| **ROS (0 h)** | Y = -1102*X + 2037 | -5812 to 3609 | 256.8 to 3817 | 0.2648 to +infinity | 0.0088 | -0.0939 |
|  |  |  |  |  |  |  |

**Supplemental Table 18 Linear regression results of correlation analyses regarding Supplemental Figure 17: correlations between endpoints for raw data at 0 h**

|  |  |  |  |  |  |  |  |
| --- | --- | --- | --- | --- | --- | --- | --- |
|  |  | **Equation** | **95% Confidence Intervals** | | | **Goodness of Fit** | **Pearson** |
|  |  |  | **Slope** | **Y-intercept** | **X-intercept** | **R squared** | **r** |
| **RAW (0 h) γH2AX:GE/ROS** | ***BBC3* (0 h)** | Y = -6.116e-009*X + 1.176e-005 | -1.891e-008 to 6.676e-009 | 6.465e-006 to 1.705e-005 | 880.3 to +infinity | 0.0358 | -0.1893 |
|  | ***CDKN1A* (0 h)** | Y = 1.213e-008*X + 3.751e-006 | -2.472e-009 to 2.674e-008 | -2.291e-006 to 9.792e-006 | -infinity to 88.19 | 0.1008 | 0.3176 |
|  | ***FDXR* (0 h)** | Y = 3.672e-010*X + 6.869e-007 | -8.204e-010 to 1.555e-009 | 1.957e-007 to 1.178e-006 | -infinity to -129.0 | 0.0153 | 0.1237 |
|  | ***GADD45A* (0 h)** | Y = 1.130e-009*X + 2.610e-006 | -2.058e-009 to 4.319e-009 | 1.291e-006 to 3.929e-006 | -infinity to -304.4 | 0.0200 | 0.1414 |
|  | ***MDM2* (0 h)** | Y = -9.440e-009*X + 2.320e-005 | -4.302e-008 to 2.414e-008 | 9.312e-006 to 3.709e-005 | 836.6 to +infinity | 0.0127 | -0.1126 |
|  | ***XPC* (0 h)** | Y = -3.303e-009*X + 9.375e-006 | -1.093e-008 to 4.321e-009 | 6.222e-006 to 1.253e-005 | 1128 to +infinity | 0.0296 | -0.1721 |
|  | **ROS (0 h)** | Y = 9.481*X - 1878 | -4.014 to 22.98 | -7460 to 3703 | -infinity to 370.2 | 0.0743 | 0.2725 |
|  |  |  |  |  |  |  |  |
| **RAW (0 h) ROS:GE/ROS (UVA)** | ***BBC3* (0 h)** | Y = -9.241e-011*X + 9.505e-006 | -4.650e-010 to 2.802e-010 | 7.822e-006 to 1.119e-005 | 21608 to +infinity | 0.0099 | -0.0995 |
|  | ***CDKN1A* (0 h)** | Y = 1.466e-010*X + 8.285e-006 | -2.922e-010 to 5.854e-010 | 6.303e-006 to 1.027e-005 | -infinity to -12308 | 0.0178 | 0.1335 |
|  | ***FDXR* (0 h)** | Y = -1.664e-011*X + 8.637e-007 | -5.037e-011 to 1.710e-011 | 7.113e-007 to 1.016e-006 | 18062 to +infinity | 0.0380 | -0.1950 |
|  | ***GADD45A* (0 h)** | Y = 2.849e-011*X + 3.004e-006 | -6.338e-011 to 1.204e-010 | 2.590e-006 to 3.419e-006 | -infinity to -23275 | 0.0154 | 0.1240 |
|  | ***MDM2* (0 h)** | Y = -3.878e-010*X + 2.019e-005 | -1.347e-009 to 5.709e-010 | 1.586e-005 to 2.452e-005 | 15912 to +infinity | 0.0259 | -0.1609 |
|  | ***XPC* (0 h)** | Y = 1.227e-010*X + 7.835e-006 | -9.417e-011 to 3.396e-010 | 6.855e-006 to 8.814e-006 | -infinity to -21644 | 0.0495 | 0.2224 |
|  | **ROS (UVA)** | Y = 0.1465*X + 5743 | -0.9597 to 1.253 | 2735 to 8752 | -infinity to -3068 | 0.0036 | 0.0600 |
|  |  |  |  |  |  |  |  |

**Supplemental Table 19 Linear regression results of correlation analyses regarding Supplemental Figure 18: correlation of injected activity (MBq) with raw results from each endpoint at 2 h considering the pool of all patients**

|  |  |  |  |  |  |  |
| --- | --- | --- | --- | --- | --- | --- |
|  | **Equation** | **95% Confidence Intervals** | | | **Goodness of Fit** | **Pearson** |
|  |  | **Slope** | **Y-intercept** | **X-intercept** | **R squared** | **r** |
| ***BBC3* (2 h)** | Y = 1.148e-008*X + 5.173e-006 | -1.436e-009 to 2.439e-008 | -1.903e-006 to 1.225e-005 | -infinity to 82.81 | 0.0958 | 0.3096 |
| ***CDKN1A* (2 h)** | Y = -2.812e-009*X + 9.774e-006 | -9.393e-009 to 3.769e-009 | 6.167e-006 to 1.338e-005 | 1368 to +infinity | 0.0239 | -0.1546 |
| ***FDXR* (2 h)** | Y = 3.874e-010*X + 5.930e-007 | -1.857e-010 to 9.605e-010 | 2.789e-007 to 9.071e-007 | -infinity to -301.3 | 0.0578 | 0.2403 |
| ***GADD45A* (2 h)** | Y = -8.930e-010*X + 3.670e-006 | -3.618e-009 to 1.832e-009 | 2.177e-006 to 5.164e-006 | 1366 to +infinity | 0.0142 | -0.1192 |
| ***MDM2* (2 h)** | Y = 3.833e-009*X + 1.700e-005 | -1.358e-008 to 2.124e-008 | 7.461e-006 to 2.654e-005 | -infinity to -368.2 | 0.0065 | 0.0804 |
| ***XPC* (2 h)** | Y = -4.183e-009*X + 1.132e-005 | -1.320e-008 to 4.831e-009 | 6.383e-006 to 1.626e-005 | 1171 to +infinity | 0.0281 | -0.1676 |
| **γH2AX (2 h)** | Y = 0.04437*X + 425.2 | -0.2172 to 0.3059 | 273.0 to 577.4 | -infinity to -914.8 | 0.0049 | 0.0697 |
| **ROS (P or S, 2 h)** | Y = -3.740*X + 3793 | -7.910 to 0.4305 | 1366 to 6220 | 707.7 to +infinity | 0.1201 | -0.3465 |
| **ROS (P or S +UVA)** | Y = 5.090*X + 3771 | -5.846 to 16.03 | -2513 to 10054 | -infinity to 169.4 | 0.0450 | 0.2121 |
|  |  |  |  |  |  |  |

**Supplemental Table 20 Linear regression results of correlation analyses regarding Supplemental Figure 19: correlation of effective (E) dose (mSv) with raw results from each endpoint at 2 h considering the pool of all patients**

|  |  |  |  |  |  |  |
| --- | --- | --- | --- | --- | --- | --- |
|  | **Equation** | **95% Confidence Intervals** | | | **Goodness of Fit** | **Pearson** |
|  |  | **Slope** | **Y-intercept** | **X-intercept** | **R squared** | **r** |
| ***BBC3* (2 h)** | Y = -1.194e-006*X + 1.798e-005 | -3.254e-006 to 8.648e-007 | 5.356e-006 to 3.060e-005 | 9.075 to +infinity | 0.0432 | -0.2078 |
| ***CDKN1A* (2 h)** | Y = 8.574e-007*X + 3.279e-006 | -1.264e-007 to 1.841e-006 | -2.750e-006 to 9.309e-006 | -infinity to 1.526 | 0.0925 | 0.3041 |
| ***FDXR* (2 h)** | Y = -5.411e-009*X + 8.177e-007 | -9.693e-008 to 8.611e-008 | 2.568e-007 to 1.379e-006 | 13.92 to +infinity | 0.0005 | -0.0217 |
| ***GADD45A* (2 h)** | Y = 2.706e-007*X + 1.618e-006 | -1.432e-007 to 6.844e-007 | -9.185e-007 to 4.154e-006 | -infinity to 1.374 | 0.0543 | 0.2330 |
| ***MDM2* (2 h)** | Y = 9.934e-007*X + 1.300e-005 | -1.690e-006 to 3.677e-006 | -3.445e-006 to 2.945e-005 | -infinity to 0.9620 | 0.0181 | 0.1344 |
| ***XPC* (2 h)** | Y = 1.086e-006*X + 2.786e-006 | -2.744e-007 to 2.447e-006 | -5.553e-006 to 1.113e-005 | -infinity to 2.331 | 0.0788 | 0.2807 |
| **γH2AX (2 h)** | Y = 8.187*X + 400.1 | -28.82 to 45.20 | 171.4 to 628.9 | -infinity to -3.854 | 0.0082 | 0.0907 |
| **ROS (P or S, 2 h)** | Y = 692.8*X - 2373 | 130.9 to 1255 | -5845 to 1100 | -7.936 to 4.934 | 0.2050 | 0.4528 |
| **ROS (P or S +UVA)** | Y = 410.4*X + 4018 | -1207 to 2028 | -5939 to 13976 | -infinity to 3.080 | 0.0138 | 0.1175 |
|  |  |  |  |  |  |  |

**Supplemental Table 21 Linear regression results of correlation analyses regarding Supplemental Figure 20: correlation of blood activity left (mBq) with raw results from each endpoint at 2 h considering the pool of all patients**

|  |  |  |  |  |  |  |
| --- | --- | --- | --- | --- | --- | --- |
|  | **Equation** | **95% Confidence Intervals** | | | **Goodness of Fit** | **Pearson** |
|  |  | **Slope** | **Y-intercept** | **X-intercept** | **R squared** | **r** |
| ***BBC3* (2 h)** | Y = 6.860e-007*X + 5.159e-006 | -2.946e-008 to 1.401e-006 | -1.543e-006 to 1.186e-005 | -infinity to 1.176 | 0.1133 | 0.3366 |
| ***CDKN1A* (2 h)** | Y = -8.980e-008*X + 9.093e-006 | -4.606e-007 to 2.810e-007 | 5.620e-006 to 1.257e-005 | 26.04 to +infinity | 0.0081 | -0.0899 |
| ***FDXR* (2 h)** | Y = 1.462e-008*X + 6.755e-007 | -1.740e-008 to 4.665e-008 | 3.755e-007 to 9.755e-007 | -infinity to -8.387 | 0.0282 | 0.1678 |
| ***GADD45A* (2 h)** | Y = -3.193e-008*X + 3.531e-006 | -1.839e-007 to 1.200e-007 | 2.107e-006 to 4.954e-006 | 25.67 to +infinity | 0.0061 | -0.0781 |
| ***MDM2* (2 h)** | Y = -2.481e-007*X + 2.086e-005 | -1.219e-006 to 7.231e-007 | 1.176e-005 to 2.996e-005 | 23.27 to +infinity | 0.0090 | -0.0948 |
| ***XPC* (2 h)** | Y = -4.718e-008*X + 9.782e-006 | -5.534e-007 to 4.590e-007 | 5.040e-006 to 1.452e-005 | 24.81 to +infinity | 0.0012 | -0.0347 |
| **γH2AX (2 h)** | Y = -3.016*X + 479.1 | -16.93 to 10.90 | 342.5 to 615.7 | 35.31 to +infinity | 0.0083 | -0.0909 |
| **ROS (P or S, 2 h)** | Y = -112.3*X + 2778 | -346.1 to 121.5 | 482.1 to 5074 | 12.76 to +infinity | 0.0393 | -0.1983 |
| **ROS (P or S +UVA)** | Y = -191.0*X + 8122 | -750.7 to 368.8 | 2674 to 13570 | 16.42 to +infinity | 0.0247 | -0.1572 |
|  |  |  |  |  |  |  |

**Supplemental Table 22 Linear regression results of correlation analyses regarding Supplemental Figure 21: correlation of percent of injected activity left with raw results from each endpoint at 2 h considering the pool of all patients**

|  |  |  |  |  |  |  |
| --- | --- | --- | --- | --- | --- | --- |
|  | **Equation** | **95% Confidence Intervals** | | | **Goodness of Fit** | **Pearson** |
|  |  | **Slope** | **Y-intercept** | **X-intercept** | **R squared** | **r** |
| ***BBC3* (2 h)** | Y = -3.631e-007*X + 1.154e-005 | -3.547e-006 to 2.821e-006 | 4.879e-006 to 1.821e-005 | 4.740 to +infinity | 0.0018 | -0.0425 |
| ***CDKN1A* (2 h)** | Y = 9.107e-009*X + 8.328e-006 | -1.553e-006 to 1.571e-006 | 5.059e-006 to 1.160e-005 | -infinity to -3.385 | 0.0000 | 0.0022 |
| ***FDXR* (2 h)** | Y = -9.813e-008*X + 9.769e-007 | -2.294e-007 to 3.315e-008 | 7.022e-007 to 1.252e-006 | 5.219 to +infinity | 0.0721 | -0.2685 |
| ***GADD45A* (2 h)** | Y = -4.209e-008*X + 3.341e-006 | -6.811e-007 to 5.970e-007 | 2.004e-006 to 4.678e-006 | 6.519 to +infinity | 0.0006 | -0.0246 |
| ***MDM2* (2 h)** | Y = -3.025e-006*X + 2.432e-005 | -6.959e-006 to 9.090e-007 | 1.609e-005 to 3.256e-005 | 4.420 to +infinity | 0.0760 | -0.2756 |
| ***XPC* (2 h)** | Y = 2.807e-008*X + 9.337e-006 | -2.097e-006 to 2.153e-006 | 4.891e-006 to 1.378e-005 | -infinity to -2.414 | 0.0000 | 0.0049 |
| **γH2AX (2 h)** | Y = -22.08*X + 492.0 | -78.02 to 33.86 | 375.9 to 608.2 | 7.528 to +infinity | 0.0269 | -0.1640 |
| **ROS (P or S, 2 h)** | Y = 195.9*X + 1431 | -768.8 to 1161 | -572.6 to 3434 | -infinity to 0.5806 | 0.0073 | 0.0853 |
| **ROS (P or S +UVA)** | Y = -2103*X + 10218 | -4106 to -100.5 | 5997 to 14438 | 3.165 to 66.32 | 0.1935 | -0.4399 |
|  |  |  |  |  |  |  |

**Supplemental Table 23 Linear regression results of correlation analyses regarding Supplemental Figure 22: correlations between endpoints for raw data at 2 h**

|  |  |  |  |  |  |  |  |
| --- | --- | --- | --- | --- | --- | --- | --- |
|  |  | **Equation** | **95% Confidence Intervals** | | | **Goodness of Fit** | **Pearson** |
|  |  |  | **Slope** | **Y-intercept** | **X-intercept** | **R squared** | **r** |
| **RAW (2 h) γH2AX:GE/ROS** | ***BBC3* (2 h)** | Y = -5.260e-009*X + 1.394e-005 | -3.141e-008 to 2.089e-008 | 1.786e-006 to 2.609e-005 | 790.3 to +infinity | 0.0065 | -0.0808 |
|  | ***CDKN1A* (2 h)** | Y = 8.702e-009*X + 4.385e-006 | -3.316e-009 to 2.072e-008 | -1.199e-006 to 9.970e-006 | -infinity to 59.69 | 0.0785 | 0.2802 |
|  | ***FDXR* (2 h)** | Y = 1.007e-010*X + 7.861e-007 | -1.043e-009 to 1.245e-009 | 2.544e-007 to 1.318e-006 | -infinity to -210.6 | 0.0013 | 0.0355 |
|  | ***GADD45A* (2 h)** | Y = 1.315e-009*X + 2.643e-006 | -3.788e-009 to 6.418e-009 | 2.723e-007 to 5.014e-006 | -infinity to -43.90 | 0.0107 | 0.1033 |
|  | ***MDM2* (2 h)** | Y = -3.791e-010*X + 2.027e-005 | -3.361e-008 to 3.285e-008 | 4.828e-006 to 3.571e-005 | 1025 to +infinity | 0.0000 | -0.0046 |
|  | ***XPC* (2 h)** | Y = -3.572e-009*X + 1.145e-005 | -2.138e-008 to 1.423e-008 | 3.172e-006 to 1.972e-005 | 886.8 to +infinity | 0.0065 | -0.0806 |
|  | **ROS (P or S, 2 h)** | Y = 2.276*X + 713.2 | -4.376 to 8.929 | -2378 to 3804 | -infinity to 290.7 | 0.0187 | 0.1366 |
|  |  |  |  |  |  |  |  |
| **RAW (2 h) ROS:GE/ROS (P/S+UVA)** | ***BBC3* (2 h)** | Y = -7.363e-010*X + 1.288e-005 | -2.283e-009 to 8.106e-010 | 8.403e-006 to 1.735e-005 | 6440 to +infinity | 0.0355 | -0.1884 |
|  | ***CDKN1A* (2 h)** | Y = 3.982e-011*X + 8.174e-006 | -7.115e-010 to 7.912e-010 | 6.002e-006 to 1.035e-005 | -infinity to -8447 | 0.0005 | 0.0214 |
|  | ***FDXR* (2 h)** | Y = -2.278e-011*X + 8.699e-007 | -9.088e-011 to 4.533e-011 | 6.730e-007 to 1.067e-006 | 10640 to +infinity | 0.0179 | -0.1336 |
|  | ***GADD45A* (2 h)** | Y = 1.285e-010*X + 3.005e-006 | -1.751e-010 to 4.320e-010 | 2.127e-006 to 3.882e-006 | -infinity to -5488 | 0.0283 | 0.1682 |
|  | ***MDM2* (2 h)** | Y = -9.630e-010*X + 2.176e-005 | -2.920e-009 to 9.936e-010 | 1.610e-005 to 2.742e-005 | 8342 to +infinity | 0.0379 | -0.1946 |
|  | ***XPC* (2 h)** | Y = 6.702e-010*X + 8.709e-006 | -3.676e-010 to 1.708e-009 | 5.708e-006 to 1.171e-005 | -infinity to -3766 | 0.0635 | 0.2519 |
|  | **ROS (P or S +UVA)** | Y = 0.1033*X + 6194 | -1.242 to 1.449 | 3088 to 9299 | -infinity to -2560 | 0.0012 | 0.0348 |
|  |  |  |  |  |  |  |  |
